# Supplementary material for: A multiscale geographically weighted regression analysis of teenage pregnancy and associated factors among adolescents aged 15 to 19 in Ethiopia using the 2019 mini-demographic and health survey
Source: PLoS One. 2024 Sep 6;19(9):e0310025. doi: 10.1371/journal.pone.0310025 (PMC11379268; doi:10.1371/journal.pone.0310025)

PLOS ONE

A multi-scale geographically weighted regression analysis of teenage pregnancy and associated factors among adolescents aged 15 to 19 in Ethiopia using the 2019 mini-demographic and health survey

--Manuscript Draft--

Manuscript Number:	PONE-D-23-27072
Article Type:	Research Article
Full Title:	A multi-scale geographically weighted regression analysis of teenage pregnancy and associated factors among adolescents aged 15 to 19 in Ethiopia using the 2019 mini-demographic and health survey
Short Title:	A multi-scale geographically weighted regression analysis of teenage pregnancy and associated factors
Corresponding Author:	Tsion Mulat Tebeje, MPH<br>Dilla University<br>Dilla, ETHIOPIA
Keywords:	Adolescents, Demographic and health survey, Ethiopia, Teenage pregnancy, MGWR
Abstract:	Background: Teenage pregnancy remains one of the major reproductive health problems, specifically in Sub-Sahara African countries. It leads to maternal complications, neonatal complications and social consequences. This study is aimed at determining the multi-scale geographically weighted regression (MGWR) analysis of teenage pregnancy among adolescents aged 15 to 19 in Ethiopia using the 2019 mini Demographic and Health Survey.<br>Methods: In this study, a total weighted sample of 2165 teenage girls aged 15 to 19 were included. Multilevel binary logistic regression model was employed using STATA version 17 and results were reported with odds ratio with 95% confidence interval and P-values below 0.05 was used to identify significant predictors. The spatial analysis was conducted by Arc-GIS version 10.7 and Python 3.To identify factors associated with the hotspots of teenage pregnancy, spatial regression was conducted and model comparison was based on adjusted R2, corrected Akaike Information Criteria (AICc) and Residual Sum of Squares (RSS).<br>Results: The prevalence of teenage pregnancy among adolescents aged 15 to 19 was 12.98% (95%CI: 11.6%, 14.5%). The significant hotspot areas were detected in central and southern Afar, northern, central and western Gambela, north eastern and south central Oromia and eastern Somali region. The MGWR analysis showed significant predictors of spatial variations of teenage pregnancy were illiteracy and being married. In multilevel analysis; teenagers aged 17 (AOR = 3.54; 95% CI: 1.60, 7.81), 18 (AOR = 8.21; 95% CI: 3.96, 17.0), and 19 (AOR = 15.0; 95% CI: 6.84, 32.9), being literate (AOR = 0.57; 95% CI: 0.35, 0.92), being married (AOR = 22.8; 95% CI: 14.1, 37.0), age of household head (AOR = 0.98; 95% CI: 0.98, 0.99) and being resident of Gambela region (AOR = 3.27; 95% CI: 1.21, 8.86) were significantly associated with teenage pregnancy among adolescents aged 15 to 19.<br>Conclusion: Teenage pregnancy is a public health problem in Ethiopia. Policymakers should make attention towards early marriage and teenage literacy. Policies to change the tradition of early marriage must be implemented. Measures must be made to improve literacy levels and reproductive health awareness.
Order of Authors:	Tsion Mulat Tebeje, MPH
	Beminate Lemma Seifu, MPH
	Mesfin Abebe, MSc
	Ever Siyoum Shewarega, MPH
	Gizaw Sisay, MSc
	Kusse Urmale Mare, MSc
	Fantu Mamo Aragaw, MPH
	Binyam Tariku Seboka, PHD fellow

Powered by Editorial Manager® and ProduXion Manager® from Aries Systems Corporation

Opposed Reviewers:	
Additional Information:	
Question	Response
**Financial Disclosure**<br><br>Enter a financial disclosure statement that describes the sources of funding for the work included in this submission. Review the submission guidelines for detailed requirements. View published research articles from *PLOS ONE* for specific examples.<br><br>This statement is required for submission and **will appear in the published article** if the submission is accepted. Please make sure it is accurate.<br><br>**Unfunded studies**<br>Enter: *The author(s) received no specific funding for this work.*<br><br>**Funded studies**<br>Enter a statement with the following details:<br>• Initials of the authors who received each award<br>• Grant numbers awarded to each author<br>• The full name of each funder<br>• URL of each funder website<br>• Did the sponsors or funders play any role in the study design, data collection and analysis, decision to publish, or preparation of the manuscript?<br>• **NO** - Include this sentence at the end of your statement: *The funders had no role in study design, data collection and analysis, decision to publish, or preparation of the manuscript.*<br>• **YES** - Specify the role(s) played.<br><br>* typeset	The author(s) received no specific funding for this work.
**Competing Interests**<br><br>Use the instructions below to enter a competing interest statement for this submission. On behalf of all authors, disclose any competing interests that could be perceived to bias this work—acknowledging all financial support and any other relevant financial or non-financial competing interests.	The authors have declared that no competing interests exist.

Powered by Editorial Manager® and ProduXion Manager® from Aries Systems Corporation

This statement is required for submission and **will appear in the published article** if the submission is accepted. Please make sure it is accurate and that any funding sources listed in your Funding Information later in the submission form are also declared in your Financial Disclosure statement.<br><br>View published research articles from *PLOS ONE* for specific examples.<br><br>**NO authors have competing interests**<br><br>Enter: *The authors have declared that no competing interests exist.*<br><br>**Authors with competing interests**<br><br>Enter competing interest details beginning with this statement:<br><br>*I have read the journal's policy and the authors of this manuscript have the following competing interests: [insert competing interests here]*<br><br>* typeset	
**Ethics Statement**<br><br>Enter an ethics statement for this submission. This statement is required if the study involved:<br><br>• Human participants<br>• Human specimens or tissue<br>• Vertebrate animals or cephalopods<br>• Vertebrate embryos or tissues<br>• Field research<br><br>Write "N/A" if the submission does not require an ethics statement.<br><br>General guidance is provided below. Consult the submission guidelines for detailed instructions. **Make sure that all**	Data access permission was received from the measure DHS program via an online request at http://www.dhsprogram.com. The data used in this research were publicly available and did not contain any personal identifiers

Powered by Editorial Manager® and ProduXion Manager® from Aries Systems Corporation

**information entered here is included in the Methods section of the manuscript.**<br><br>**Format for specific study types**<br><br>**Human Subject Research (involving human participants and/or tissue)**<br>• Give the name of the institutional review board or ethics committee that approved the study<br>• Include the approval number and/or a statement indicating approval of this research<br>• Indicate the form of consent obtained (written/oral) or the reason that consent was not obtained (e.g. the data were analyzed anonymously)<br><br>**Animal Research (involving vertebrate animals, embryos or tissues)**<br>• Provide the name of the Institutional Animal Care and Use Committee (IACUC) or other relevant ethics board that reviewed the study protocol, and indicate whether they approved this research or granted a formal waiver of ethical approval<br>• Include an approval number if one was obtained<br>• If the study involved *non-human primates*, add *additional details* about animal welfare and steps taken to ameliorate suffering<br>• If anesthesia, euthanasia, or any kind of animal sacrifice is part of the study, include briefly which substances and/or methods were applied<br><br>**Field Research**<br><br>Include the following details if this study involves the collection of plant, animal, or other materials from a natural setting:<br>• Field permit number<br>• Name of the institution or relevant body that granted permission	
**Data Availability**<br><br>Authors are required to make all data underlying the findings described fully available, without restriction, and from the time of publication. PLOS allows rare exceptions to address legal and ethical	Yes - all data are fully available without restriction

concerns. See the PLOS Data Policy and FAQ for detailed information.<br><br>A Data Availability Statement describing where the data can be found is required at submission. Your answers to this question constitute the Data Availability Statement and **will be published in the article**, if accepted.<br><br>**Important:** Stating 'data available on request from the author' is not sufficient. If your data are only available upon request, select 'No' for the first question and explain your exceptional situation in the text box.<br><br>Do the authors confirm that all data underlying the findings described in their manuscript are fully available without restriction?	
**Describe where the data may be found in full sentences. If you are copying our sample text, replace any instances of XXX with the appropriate details.**<br><br>• If the data are **held or will be held in a public repository**, include URLs, accession numbers or DOIs. If this information will only be available after acceptance, indicate this by ticking the box below. For example: *All XXX files are available from the XXX database (accession number(s) XXX, XXX.*).<br>• If the data are all contained **within the manuscript and/or Supporting Information files**, enter the following: *All relevant data are within the manuscript and its Supporting Information files.*<br>• If neither of these applies but you are able to provide **details of access elsewhere**, with or without limitations, please do so. For example:<br><br>*Data cannot be shared publicly because of [XXX]. Data are available from the XXX Institutional Data Access / Ethics Committee (contact via XXX) for researchers who meet the criteria for access to confidential data.*	The data used in this research are publicly available from the measure DHS program via an online request at http://www.dhsprogram.com

The data underlying the results presented in the study are available from (include the name of the third party and contact information or URL).<br>• This text is appropriate if the data are owned by a third party and authors do not have permission to share the data.<br><br>* typeset	
Additional data availability information:	

Editor in Chief

PloS One

Date: 08/23/2023

Dear editor,

I am writing to submit a manuscript entitled, "**A multi-scale geographically weighted regression analysis of teenage pregnancy and associated factors among adolescents aged 15 to 19 in Ethiopia using the 2019 mini- demographic and health survey"**. We believe that our manuscript has been conducted precisely and hope that it will be accepted for publication in the PloS One Journal.

The study is intended to model spatial determinants of teenage pregnancy through identification of geographic locations with higher magnitude of the outcome and predict teenage pregnancy. The study was conducted to overcome the limitation of previous studies which utilized Geographic Weighted Regression for the relationship between the dependent and independent variables. Multiscale Geographic Weighted Regression consider multiple scales and relaxes the assumption of GWR by determining bandwidths which are parameter specific. This allows the relationships between independent and dependent variables to vary at different spatial scales.

We would like to thank you once again for your consideration of our work. We look forward to hearing from you. With the submission, the authors confirm no conflict of interest and also confirm that the manuscript has not been published nor is under consideration else.

Thank you for your time and consideration.

Yours sincerely,

Tsion Mulat Tebeje

School of Public Health, College of Health Science and Medicine, Dilla University, Dilla, Ethiopia.

Email: yemarina12@gmail.com

Manuscript Click here to access/download;Manuscript;Manuscript.docx

A multi-scale geographically weighted regression analysis of teenage pregnancy and associated factors among adolescents aged 15 to 19 in Ethiopia using the 2019 mini-demographic and health survey

Tsion Mulat Tebeje[1], Beminate Lemma Seifu[2], Mesfin Abebe[3], Ever Siyoum Shewarega[4], Gizaw Sisay[1], Kusse Urmale Mare[5], Fantu Mamo Aragaw[6], Binyam Tariku Seboka[1,7]

1. School of Public Health, College of Health Science and Medicine, Dilla University, Dilla, Ethiopia
2. Department of public health, College of Medicine and Health Sciences, Samara 19 University, Afar, Ethiopia
3. Department of Midwifery, College of Health Science and Medicine, Dilla University, Dilla, Ethiopia
4. Department of Reproductive Health, School of Public Health, College of Health Science and Medicine, Dilla University, Dilla, Ethiopia
5. Department of Nursing, College of Medicine and Health Sciences, Samara University, 17 Afar, Ethiopia
6. Department of Epidemiology and Biostatistics, Institute of Public Health, College of Medicine and Health Sciences, University of Gondar, Gondar, Ethiopia
7. Health & Environment Research (HER) Lab, Azrieli Faculty of Medicine, Bar Ilan University, Safed, Israel

Abstract

Background: Teenage pregnancy remains one of the major reproductive health problems, specifically in Sub-Sahara African countries. It leads to maternal complications, neonatal complications and social consequences. This study is aimed at determining the multi-scale geographically weighted regression (MGWR) analysis of teenage pregnancy among adolescents aged 15 to 19 in Ethiopia using the 2019 mini Demographic and Health Survey.

Methods: In this study, a total weighted sample of 2165 teenage girls aged 15 to 19 were included. Multilevel binary logistic regression model was employed using STATA version 17 and results were reported with odds ratio with 95% confidence interval and P-values below 0.05 ~~was~~ used to identify significant predictors. The spatial analysis was conducted by Arc-GIS version 10.7 and Python 3.To identify factors associated with the hotspots of teenage pregnancy, spatial regression was conducted and model comparison was based on adjusted R2, corrected Akaike Information Criteria (AICc) and Residual Sum of Squares (RSS).

Results: The prevalence of teenage pregnancy among adolescents aged 15 to 19 was 12.98% (95%CI: 11.6%, 14.5%). The significant hotspot areas were detected in central and southern Afar, northern, central and western Gambela, north eastern and south central Oromia and eastern Somali region. The MGWR analysis showed significant predictors of spatial variations of teenage pregnancy were illiteracy and being married. In multilevel analysis; teenagers aged 17 (AOR = 3.54; 95% CI: 1.60, 7.81), 18 (AOR = 8.21; 95% CI: 3.96, 17.0), and 19 (AOR = 15.0; 95% CI: 6.84, 32.9), being literate (AOR = 0.57; 95% CI: 0.35, 0.92), being married (AOR = 22.8; 95% CI: 14.1, 37.0), age of household head (AOR = 0.98; 95% CI: 0.98, 0.99) and being resident of Gambela region (AOR = 3.27; 95% CI: 1.21, 8.86) were significantly associated with teenage pregnancy among adolescents aged 15 to 19.

Conclusion: Teenage pregnancy is a public health problem in Ethiopia. Policymakers should make attention towards early marriage and teenage literacy. Policies to change the tradition of early marriage must be implemented. Measures must be made to improve literacy levels and reproductive health awareness.

Key words: Adolescents, Demographic and health survey, Ethiopia, Teenage pregnancy, MGWR

Introduction

The largest proportion of teenage childbirths are occurred in developing countries, where most of the girls marry earlier [1]. About 20 countries from Sub-Saharan Africa (SSA) and Asia accounts for 82% of adolescent maternal mortality [2]. As compared to women aged 20 to 24 years, those aged 15 to 19 have 28% higher risk of maternal mortality ratio [3]. Among adolescent girls aged 15-19, pregnancy related conditions are major causes of death worldwide [4].

Globally 16 million girls aged 15 to 19 become pregnant annually, which comprises 11% of all births around the world [5]. While there has been a decrease in the rate of adolescent births, the total number of births has not decreased as much due to the increasing size of the adolescent population [6]. Each year adolescents from low and middle income countries (LMIC) have an estimated 21 million pregnancies which resulted in 12 million births [7]. Half of the pregnancies are unintended, and 55% of the unintended pregnancies often end up in abortion, which are usually unsafe [8]. Every year, 3.2 million unsafe abortions takes place among adolescents aged 15 to 19, which accounts 15% of the total global incidence [9].

Adolescents have a significant unmet need for sexual and reproductive health care. Girls aged 15 to 19 have 43% unmet need for modern contraception than reproductive age group women with 24% unmet need [7]. Adolescent pregnancies resulted in health consequences with increased risks

of eclampsia, puerperal endometritis, and systemic infections. Social consequences including stigma, rejection, violence by partners or parents, dropping out of school are also common [10]. Additionally it is related with ~~worse~~ child survival which is attributable to biological and social risks [11].

Teenage pregnancy is a public health concern in Ethiopia ~~with~~ limited knowledge and access to reproductive health services [12]. The country was found to be the 3rd country with highest number of adolescent maternal death [2]. According to the 2000, 2005, 2011 and 2016 ~~EDHS~~, teenage pregnancy rates in Ethiopia were 16%, 17%, 12% and 13%, respectively [13–16]. When stratified by region, it is highest in Afar (23%) and lowest in the capital, Addis Ababa (3%) [16].

According to previously conducted ~~researches~~, factors which were found to be related with adolescent pregnancy specifically in LMIC are: lower education and socio economic status, child marriage, not utilizing ~~contraceptive non-use and~~ child sexual abuses and early sex, age, religion, , lack of knowledge of reproductive and sexual health, gender inequity, physical or sexual violence, lower family income, being in the 18-19 year age group, maternal history of teenage pregnancy and not communicating with parents about reproductive health issues [17–22].

Fertility and childbearing among young people ~~is one of the neglected experiences~~ in Ethiopia. Despite similar studies conducted in the country, this study utilized the dataset from the most recent Ethiopian demographic and health survey (EDHS), mini-EDHS 2019. In addition to utilizing the most recent data, the study was conducted to overcome the limitation of previous studies which utilized Geographic Weighted Regression (GWR) for the relationship between the dependent and independent variables. GWR assumes the relationship between the outcome and predictor variables operate at the same spatial scale. This violates the laws of heterogeneity or non-stationarity of geospatial relationships by ignoring the truth that different relationships may occur

at various scales. MGWR consider multiple scales and relaxes the assumption of GWR by determining bandwidths which are parameter specific. This allows the relationships between independent and dependent variables to vary at different spatial scales [23–25]. The aim of this study is to better target the spatial context of determinants of teenage pregnancy using an explicitly multi-scale approach (MGWR). Therefore the study is intended to model spatial determinants of teenage pregnancy through identification of geographic locations with higher magnitude of the outcome and predict teenage pregnancy among adolescents aged 15 to 19 in Ethiopia.

Methods and Materials

A community based cross-sectional study was conducted in Ethiopia by using mini-EDHS 2019. Ethiopia is the second most populous country in Africa next to Nigeria with total population of almost 126.5 million people in 2023 and an area of 1.1 million square kilometers. Children under age of 15, individuals age 15 to 64 and those 65 or above represent 44%, 52% and 4% of the total population, respectively [26,27]. The country is subdivided into nine regions and two city administrations.

The 2019 mini-EDHS sample was stratified and selected in two stages. Each region was stratified into urban and rural areas, yielding 21 sampling strata. Samples of Enumeration Areas (EAs) were selected independently in each stratum in two stages. The first stage involves selection of 305 EAs, with 93 in urban areas and 212 in rural areas. In the second stage, a fixed number of 30 households per cluster were selected with an equal probability of systematic selection from the newly created household listing [26].

The outcome variable is teenage pregnancy which is defined as percentage of women aged 15-19 who have had a live birth, are pregnant with their first child and have begun childbearing [28]. It

is dichotomized as 0 = no (those who did not experience teenage pregnancy) and 1 = yes (those who experienced teenage pregnancy). The percentage of women who have begun childbearing was calculated by dividing the number of women who either have had a birth or who are pregnant at the time of interview by all women aged 15-19 [28]. The independent variables included were: age, marital status, religion, sex of household head, education level, wealth index, media exposure and contraceptive utilization. The community level variables were residence and region and aggregate community level variables, which were created by aggregating individual level factors at the cluster level, were community education, community poverty, community contraceptive utilization, community media exposure and community percentage of marriage.

Data processing and analysis

Factors associated with teenage pregnancy

Because of the hierarchical nature of the EDHS data, teenagers in the same cluster share similar characteristics than those in another cluster. Therefore; a multilevel binary logistic regression model which accounts the heterogeneity between clusters was conducted. To assess the clustering effect, Intra-class Correlation Coefficient (ICC) was estimated. The ICC showed a significant clustering effect (ICC = 20.9%). The median odds ratio (MOR), which is the median value of the odds ratio between the highest risk area and lowest risk area when randomly picking out two areas, is the increased risk that would have when moving to a different area with higher level of risk [29]. Proportional change in variance (PCV) is the variation in the dependent variable explained by all individual and community level factors [30].

Four models were constructed for the multilevel logistic regression analysis. The first model was a null model without explanatory variables to determine the degree of cluster variation in teenage pregnancy. The second model was fitted with individual-level variables; the third model was fitted

with community-level variables and the fourth was fitted with both individual and community level variables at the same time. Variables with p-value < 0.25 in bi-variable analysis were added in the multivariable model. The association between the dependent and independent variables was assessed and presented using the adjusted odds ratio (AOR) with 95% confidence interval (CI) at p-value < 0.05.

Spatial analysis

A spatial analysis was conducted by ArcGIS version 10.7 and python 3. To assess whether the spatial distribution of teenage pregnancy in Ethiopia is dispersed, clustered or randomly distributed, a global spatial autocorrelation (Global Moran's I) was utilized. A Moran's I value < 0.05 showed that the distribution of teenage pregnancy in Ethiopia is non-random [31]. Hotspot analysis was conducted to identify statistically significant clustering areas using the Getis-ordGi* statistics, which results in z-score and p-value to detect where high and low values of teenage pregnancy cluster spatially [32].

After identification of high risk areas (hotspot areas) of teenage pregnancy, factors which contribute to the spatial clustering of the outcome on these areas was determined by a global and local spatial regression model; Ordinary Least Square (OLS) and (GWR and MGWR), respectively. The spatial no stationary of proportion of teenage pregnancy was ascertained by using global spatial autocorrelation before fitting OLS, GWR and MGWR.

First OLS was fitted to choose variables which are appropriate for the spatial variation of teenage pregnancy. The six assumptions of OLS model was checked using exploratory regression before proceeding to the GWR model. Exploratory regression is a data mining tool which helps to see which combinations of explanatory variables pass all of the necessary OLS diagnostics [33]. The explanatory variables should have the expected relationship, statistical significance of each

explanatory variable, randomness of residuals which are normally distributed with no spatial patterns, non-significant Jarque-Bera statistics, explanatory variables must be free from multicollinearity (VIF < 7.5) and strength of the adjusted R^2 was checked [34]. The koenker Breusch-Pagan (BP) test was significant that shows non-statonarity or heterogeneity of the relationship between independent and dependent variables. Therefore, the local spatial regression model (GWR), which assumes the relationship between variables varies spatially, was applied [35].

A geographically weighted regression model was used to determine the predictor variables for teenage pregnancy among adolescents aged 15 to 19. Proportion of the outcome variable and all predictors was calculated for each cluster. GWR was conducted by using python 3 software. Classical GWR assumes that all of the processes being modeled operate at the same spatial scale. However, the MGWR reduces this assumption by allowing different processes to operate at different spatial scales by deriving an optimal bandwidth vector in which each element indicates the spatial scale at which a particular process takes place. Hence, MGWR is preferable as covariate-specific bandwidths are obtained than a single average bandwidth and provides valuable information on the scale at which different processes operate. Model calibration and bandwidth vector selection in MGWR were conducted using a back-fitting algorithm. Model fitness was compared among the global and local models using the Akaike information criterion (AIC), residual sum of squares (RSS), and adjusted R-squared values [36,37].

Result

Descriptive results

A total of weighted 2165 teenage girls aged 15 to 19 were included in the study. The median age of the respondent's age was 17. One-fifth (20.4%) of participants were married and only 9.5% of the girls utilized contraceptives. One-third of the girls are urban residents while a quarter of them (25.1%) are illiterate. Regarding regional states, the majority of the participants were from the Oromia region 851(39.3%) and Harari regional state had the minority of them 7 (0.3%). (Table 1)

Table 1: Descriptive characteristics of the study participants with prevalence of teenage pregnancy in Ethiopia, 2019 EDHS

Variables		Weighted frequency (%)	Teenage pregnancy	
			Yes (%)	No (%)
Age	15	506 (23.4)	6 (0.27)	500 (23.1)
	16	451 (20.8)	21 (0.97)	430 (19.9)
	17	328 (15.2)	37 (1.71)	291 (13.4)
	18	608 (28.1)	139 (6.42)	468 (21.6)
	19	272 (12.6)	77 (3.56)	195 (9.0)
Religion	Orthodox	928 (42.9)	86 (3.97)	842 (38.9)
	Muslim	589 (27.2)	79 (3.64)	510 (23.6)
	Protestant	614 (28.4)	114 (5.27)	499 (23.0)
	Others	34 (1.6)	2 (0.09)	33 (1.5)
Literacy	Illiterate	543 (25.1)	161 (7.4)	382 (17.6)
	Literate	1622 (74.9)	120 (5.5)	1502 (69.4)
Education level	No education	229 (10.6)	75 (3.5)	154 (7.1)
	Primary	1437 (66.4)	177 (8.2)	1259 (58.2)
	Secondary	432 (19.9)	19 (0.88)	413 (19.1)
	Higher	67 (3.1)	10 (0.46)	57 (2.6)

Marital status	Single	1725 (79.7)	50 (2.3)	1675 (77.4)
	Married	441 (20.4)	231 (10.7)	209 (9.7)
Contraceptive utilization	Yes	206 (9.5)	93 (4.3)	113 (5.2)
	No	1959 (90.5)	188 (8.7)	1772 (81.8)
Wealth index	Poor	720 (33.2)	124 (5.7)	596 (27.5)
	Middle	442 (20.4)	71 (3.3)	371 (17.1)
	Rich	1004 (46.4)	87 (4.0)	917 (42.4)
Media exposure	No	1361 (62.9)	193 (8.9)	1168 (53.9)
	Yes	804 (37.1)	88 (4.1)	716 (33.1)
Sex of house hold head	Male	1658 (76.6)	240 (11.1)	1418 (65.5)
	Female	507 (23.4)	41 (1.9)	466 (21.5)
**Community-level variables**				
Residence	Urban	720 (33.2)	71 (3.3)	648 (29.9)
	Rural	1445 (66.8)	210 (9.7)	1236 (57.1)
Region	Tigray	138 (6.4)	16 (0.74)	122 (5.63)
	Afar	16 (0.75)	6 (0.28)	12 (0.55)
	Amhara	498 (23.0)	35 (1.62)	464 (21.4)
	Oromia	851 (39.3)	129 (5.95)	722 (33.3)
	Somali	111 (5.1)	19 (0.89)	91 (4.2)
	Benishangul	25 (1.2)	5 (0.23)	20 (0.92)
	SNNPR	402 (18.6)	63 (2.91)	339 (15.7)
	Gambella	9 (0.42)	3 (0.14)	7 (0.32)
	Harari	6 (0.29)	1 (0.05)	5 (0.23)
	Addis Ababa	93 (4.3)	4 (0.18)	89 (4.1)
	Dire Dawa	15 (0.7)	2 (0.09)	13 (0.6)
Community media exposure	Low proportion	1116 (51.5)	141 (6.51)	974 (44.9)
	High proportion	1050 (48.5)	140 (6.47)	910 (42.0)
Community education level	Low proportion	869 (40.1)	174 (8.0)	695 (32.1)
	High proportion	1296 (59.9)	107 (4.9)	1189 (54.9)
	Low proportion	991 (45.8)	155 (7.2)	836 (38.6)

Community wealth index	High proportion	1174 (54.2)	126 (5.8)	1048 (48.4)
Community contraceptive use	Low proportion	1142 (52.7)	119 (5.5)	1023 (47.3)
	High proportion	1023 (47.3)	163 (7.5)	861 (39.8)
Community literacy	Low proportion	1014 (46.8)	203 (9.4)	811 (37.5)
	High proportion	1151 (53.2)	78 (3.6)	1073 (49.6)
Community marital status	Low proportion	1083 (50.0)	55 (2.5)	1028 (47.5)
	High proportion	1082 (50.0)	226 (10.4)	856 (39.5)

Prevalence of teenage pregnancy in Ethiopia

The prevalence of teenage pregnancy among adolescents aged 15 to 19 was 12.98% (95%CI: 11.6%, 14.5%). The highest and lowest prevalence was observed in Afar region (28.3%) and Addis Ababa (3.8%), respectively.

Spatial analysis

The global spatial autocorrelation analysis showed that the spatial distribution of teenage pregnancy in Ethiopia was significantly varied across the country with Global Moran's Index value of 0.04 with p-value < 0.001. The Z-score of 4.71 indicated that there is less than 1% likelihood that the clustered pattern could be a result of random chance. (Figure 1)

Figure 1: The global spatial autocorrelation analysis of teenage pregnancy among adolescents aged 15 to 19 in Ethiopia, 2019

The statistically significant hotspot areas (areas with high proportion of teenage pregnancy) of teenage pregnancy were observed in central and southern Afar, northern, central and western Gambela, north eastern and south central Oromia and eastern Somali region. Significant cod spot

areas (areas with low proportion of teenage pregnancy) were identified in central and southern Amhara, central Oromia, Addis Ababa and northern SNNPR. (Figure 2)

Figure 2: The Getis Ord Gi statistical analysis of hotspots of teenage pregnancy among adolescents aged 15 to 19 in Ethiopia, 2019

Spatial regression analysis

To explore assumptions of spatial regression and to estimate coefficients of the selected variables on teenage pregnancy, the OLS regression was conducted. The OLS model identified predictors of each hotspots of teenage pregnancy. The Joint F-statistics and Wald statistics were significant which shows the model was statistically significant. The Koenker statistics was statistically significant, which is indicative of non-stationary or heterogeneity of the relationship between the dependent and the independent variables across the study areas. Therefore, the local model GWR was applied as it assumes the relationship between the independent and the dependent variables has spatial heterogeneity (as confirmed by Koenker statistics). No multicollinearity was observed between the selected explanatory variables. (Table 2)

Table 2: The ordinary least square analysis (OLS) result, 2019 EDHS

Variable	Coefficient	Robust SE	Robust t-statistics	Robust probability	VIF
Intercept	0.015	0.01	1.48	0.14	---
Proportion of poor wealth index	0.016	0.03	0.47	0.64	3.30
Proportion of married teenagers	0.58	0.05	10.85	0.000000*	1.24
Proportion of Muslim teenagers	-0.026	0.017	-1.54	0.13	1.21
Proportion of illiterate teenagers	0.098	0.047	2.09	0.036839*	2.95
Proportion of community poverty	0.025	0.02	1.05	0.29	2.96
Proportion of community illiteracy	-0.040	0.02	-1.77	0.079	2.51

**Ordinary least square regression diagnostics**			
Number of observations	303	Adjusted R-squared	0.547
Joint F-statistics	61.7	Prob(>F), (6,296) degree of freedom	0.000000*
Joint Wald statistics	258.7	Prob (> chi-squared), (6) degree of freedom	0.000000*
Koenker (BP) statistics	49.9	Prob (> chi-squared), (6) degree of freedom	0.000000*
Jarque–Bera	60.1	Prob (> chi-squared), (2) degree of freedom	0.000000*

The local models GWR and MGWR were implemented to capture the spatial heterogeneity. Both of the local models had better model fitness than the global model. The adjusted R^2 improved from 0.547 (OLS) to 0.634 (GWR) and 0.691 (MGWR). The adjusted R^2 indicates the local models can explain more than % of the model variance. The residual sum of squares (RSS) which indicates unexplained variations, was very high in the OLS model (134.6) and It was 93.7 (GWR) and 78.4 (MGWR). The local regression shows an AICc of 630.4, while the GWR reduced the AICc to 618.6 and the MGWR further brings AICc down to 570.4. Relying on this information, it was confirmed that the local models has improved the model performance. (Table 3)

Table 3: Comparison of goodness of it measures between global and local models

Model comparison parameter	Adj-R^2	RSS	AICc
Model			
OLS	0.547	134.573	630.444
GWR	0.634	93.709	618.603
MGWR	0.691	78.424	570.350

The GWR model has a single bandwidth of 103 thus considering 103 nearest neighbors to inform the construction of parameter estimates in each local regression point. The use of the 103 bandwidth for each variable, correctly classifies 63.4% of the observations. In the case of MGWR, the optimum bandwidth for each variable is computed. By allowing multiple bandwidths in

MGWR, the model accounts for an optimal number of neighbors for each parameter estimate, thus allowing better predictions for the response variables, theoretically. The GWR model has a universal bandwidth of 103. In the case of MGWR, the bandwidth for each parameter are 111 for poor wealth index, 43 for being married, 301 for being Muslim, 301 for being illiterate, 292 for community poverty and 161 for community illiteracy. (Table 4)

Table 4: Summary of the GWR and MGWR model result among predictors of teenage pregnancy in Ethiopia, 2019 EDHS

Variable	Mean	STD	Min	Median	Max	Bandwidth
**GWR model**						
Intercept	-0.034	0.093	-0.231	-0.017	0.284	103
Proportion of poor wealth index	0.056	0.160	-0.249	0.051	0.399	103
Proportion of married teenagers	0.670	0.137	0.302	0.700	1.012	103
Proportion of Muslim teenagers	-0.071	0.074	-0.259	-0.062	0.120	103
Proportion of illiterate teenagers	0.135	0.106	-0.305	0.115	0.388	103
Proportion of community poverty	0.037	0.178	-0.328	0.077	0.348	103
Proportion of community illiteracy	-0.060	0.149	-0.330	-0.043	0.381	103
**MGWR model**						
Intercept	-0.052	0.197	-0.373	-0.079	0.662	50
Proportion of poor wealth index	-0.008	0.101	-0.222	-0.050	0.292	111
Proportion of married teenagers	0.640	0.250	-0.087	0.678	1.128	43
Proportion of Muslim teenagers	-0.067	0.014	-0.094	-0.065	-0.050	301
Proportion of illiterate teenagers	0.187	0.007	0.175	0.190	0.200	301
Proportion of community poverty	0.061	0.019	0.025	0.058	0.096	292
Proportion of community illiteracy	-0.051	0.054	-0.141	-0.065	0.064	161

The figures below show the mapped GWR and MGWR coefficient estimates for the intercept and the covariates. In figure 3, the local intercept in MGWR is interpreted as the value of the outcome

that would be expected if every location had exactly the same average value of each covariate. Based on the mapping of the intercept coefficients, the local intercept in the MGWR model can be interpreted as showing the level of teenage pregnancy that would be expected in each region if each of the region had an average population profile or holding all covariates constant. There is high rate of teenage pregnancy in Gambella, some parts of western and north eastern Oromia. On the contrary there is a lower rate of teenage pregnancy in central and western Amhara. The intercept in the GWR was not statistically different from zero, but there is an outlier with a positive relationship in the southern Afar at borderline with Oromia.

Figure 3: GWR (left) and MGWR (right) parameter estimates for the intercept to show local patterns of spatial heterogenity. Grey dots are not statistically different from zero

Parameter estimates for proportion of being married has a positive association with teenage pregnancy in Tigray, Amhara, Afar, Somali, eastern SNNPR, Benishangul, Gambela, Harari, Dire Dawa and eastern Oromia. In the GWR model, it showed a positive relationship with the outcome variable across the study area with no spatial heterogeneity. (Figure 4)

Figure 4: GWR (left) and MGWR (right) parameter estimates for the proportion of married to show local patterns of spatial heterogeneity. Grey dots are not statistically different from zero

The proportion of being illiterate does not show any spatial heterogeneity as it has a positive association with teenage pregnancy across the country. In the GWR model, it was not statistically different from zero as it has no statistically non-zero parameter estimate. (Figure 5)

Figure 5: GWR (left) and MGWR (right) parameter estimates for the proportion of illiterate to show local patterns of spatial heterogeneity. Grey dots are not statistically different from zero

Multi-level analysis of factors associated with teenage pregnancy

Multilevel mixed-effect logistic regression analysis was conducted to identify individual and community level variables which are significantly associated with the outcome teenage pregnancy. The value of ICC in the null model was 0.209, which shows 20.9% of variations of teenage pregnancy is explained by community level variables (between cluster variability). According to the value of the MOR in the null model (2.43), there was a variation in teenage pregnancy between clusters. When a teenage girl is switched from a low risk cluster to a high risk cluster, she would have 2.43 times higher odds of having teenage pregnancy. The final model (model 4) PCV value showed that 72% of the total variability in teenage pregnancy is explained by both the individual and community level factors. Model 4 was the best fitted model as it attains the highest value of log likelihood and the lowest value of deviance. (Table 5)

Table 5: Parameters and model fit statistics for multi-level models

Parameters	Null model	Model 2	Model 3	Model 4
Community level variance (SE)	0.87	0.46	0.015	0.24
ICC	0.21	0.12	0.0046	0.069
MOR	2.43	1.91	1.125	1.589
PCV	Ref	0.47	0.98	0.72
**Model fitness**				
Deviance	1715.1	946.82	1536.88	920.48
Log-likelihood	-857.55	-473.41	-768.44	-460.24

Based on findings of model 4, factors which were found to have a significant association with teenage pregnancy were: age of the girls, literacy, marital status, age of household head and being resident of Gambela region.

The odds of having teenage pregnancy among girls aged 17, 18 and 19 were increased by 3.5 times (AOR = 3.54; 95% CI: 1.60, 7.81), 8.2 times (AOR = 8.21; 95% CI: 3.96, 17.0) and 15 times (AOR = 15.0; 95% CI: 6.84, 32.9), respectively as compared to teenagers at 15 years. Literate teenagers have 43% (AOR = 0.57; 95% CI: 0.35, 0.92) lower risk of teenage pregnancy than those who are illiterate. Married teenagers had 22.8 (AOR = 22.8; 95% CI: 14.1, 37.0) times higher odds of teenage pregnancy compared to the single ones. For a one year increase in age of household head, the odds of experiencing teenage pregnancy is decreased by 2% (AOR = 0.98; 95% CI: 0.98, 0.99). Regarding regions, teenagers residing in Gambela region had 3.27 (AOR = 3.27; 95% CI: 1.21, 8.86) times higher odds of teenage pregnancy as compared to those residing in Tigray region. (Table 6)

Table 6: Multilevel analysis of factors associated with teenage pregnancy among adolescents aged 15 to 19 in Ethiopia, 2019 EDHS

Explanatory variable	Null model	Model 2 AOR (95%CI)	Model 3 AOR (95%CI)	Model 4 AOR (95%CI)
**Age**				
15		1		1
16		1.68 (0.70, 3.99)		1.77 (0.75, 4.18)
17		3.63 (1.64, 8.04)		3.54(1.60,7.81)**
18		8.38 (4.03, 17.4)		8.21(3.96,17.0)**
19		15.3 (7.02, 33.7)		15.0(6.84,32.9)**
**Religion**				

Orthodox		1		1
Muslim		1.13 (0.69, 1.81)		0.89 (0.47, 1.68)
Protestant		2.14 (1.26, 3.64)		1.62 (0.85, 3.10)
Others		1.46 (0.35, 6.07)		1.08 (0.24, 4.83)
**Literacy**				
Illiterate		1		1
Literate		0.65 (0.41, 1.02)		0.57 (0.35, 0.92)*
**Education level**				
No education		1		1
Primary		0.77 (0.46, 1.29)		0.84 (0.48, 1.45)
Secondary		0.59 (0.28, 1.26)		0.71 (0.32, 1.54)
Higher		0.37 (0.11, 1.30)		0.39 (0.11, 1.46)
**Marital status**				
Single		1		1
Married		24.03(15.3,37.6)		22.8(14.1,37.0)**
**Contraceptive utilization**				
Yes		1		1
No		0.97 (0.58, 1.63)		1.04 (0.59, 1.81)
**Wealth index**				
Poor		1		1
Middle		0.97 (0.57, 1.67)		1.11 (0.63, 1.93)
Rich		0.49 (0.29, 0.80)		0.59 (0.32, 1.11)
**Media exposure**				
No		1		1
Yes		1.25 (0.80, 1.93)		1.19 (0.74, 1.91)
**Age of HHH**		0.98 (0.97, 0.99)		0.98 (0.98, 0.99)*
**Residence**				
Urban			1	1
Rural			1.03 (0.68, 1.58)	0.92 (0.50, 1.68)
**Region**				

Tigray			1	1
Afar			1.31 (0.68, 2.51)	1.31 (0.44, 3.92)
Amhara			0.50 (0.25, 1.01)	0.45 (0.17, 1.18)
Oromia			1.07 (0.59, 1.95)	1.03 (0.39, 2.70)
Somali			1.16 (0.59, 2.28)	1.04 (0.34, 3.26)
Benishangul			1.36 (0.72, 2.59)	1.34 (0.48, 3.71)
SNNPR			1.04 (0.55, 1.95)	0.77 (0.28, 2.13)
Gambella			2.04 (1.09, 3.81)	3.27 (1.21, 8.86)*
Harari			1.42 (0.70, 2.86)	1.26 (0.39, 4.07)
Addis Ababa			0.80 (0.29, 2.25)	0.86 (0.23, 3.21)
Dire Dawa			1.02 (0.52, 1.99)	1.19 (0.39, 3.57)
**Community media exposure**				
Low proportion			1	1
High proportion			0.89 (0.65, 1.23)	0.93 (0.50, 1.68)
**Community education level**				
Low proportion			1	1
High proportion			0.81 (0.58, 1.12)	0.81 (0.49, 1.32)
**Community wealth index**				
Low proportion			1	1
High proportion			0.77 (0.53, 1.12)	0.83 (0.45, 1.54)
**Community contraceptive use**				
Low proportion			1	1
High proportion			1.25 (0.91, 1.71)	1.00 (0.61, 1.63)
**Community literacy**				
Low proportion			1	1
High proportion			0.89 (0.63, 1.25)	1.28 (0.76, 2.15)
**Community marital status**				
Low proportion			1	1
High proportion			4.84 (3.35, 7.00)	1.34 (0.79, 2.25)
Intercept	0.14 (0.11, 0.17)	0.03(0.009, 0.08)	0.07(0.03, 0.15)	0.02(0.005, 0.12)

*p-value < 0.05, ** p-value < 0.01, AOR: Adjusted Odds Ratio, HHH: Household Head

Discussion

The spatial statistics showed that the spatial distribution of teenage pregnancy among adolescents aged 15 to 19 had a significant variation in Ethiopia. The hotspot areas of teenage pregnancy were identified in central and southern Afar, northern, central and western Gambela, north eastern and south central Oromia and eastern Somali region. The possible explanation might be most of these areas are pastoralists and semi-pastoralists, characterized by seasonal mobility and lower utilization of contraceptives [38]. They also live in traditional settings and are strongly adhere to traditional cultural values and beliefs which leads to poor sexual and reproductive health outcomes [39].

In the spatial regression analysis of the global model the significant predictors of hotspot areas of teenage pregnancy among adolescents aged 15 to 19 were marital status and illiteracy. Local models were applied to the same predictors used in OLS to explore the local spatial variation in the relationship with the proportion of teenage pregnancy. As the MGWR model had the smallest AICc and RSS values and the highest adjusted R^2 value, it is the statistically preferable local model which is in line with previous studies [40].

The MGWR analysis revealed a significant positive relationship between the proportion of being married and teenage pregnancy in central and southern Afar, most parts of Amhara, Tigray and Benishangul, eastern SNNPR, Gambela, Harari, Dire Dawa and eastern Oromia. This is because adolescents who are married are exposed to early sexual debut which results in teenage pregnancy [41]. In most parts of Ethiopia, specifically rural areas, it is common that married girls need to get pregnant soon after their marriage to prove their fertility. Additionally, family planning utilizations

are usually disapproved by the partner, in-laws and the community as a whole as a result of social norms and little awareness and negative perception also have a role [42,43].

The proportion of being illiterate had a significant positive association with teenage pregnancy throughout the country in MGWR model. This is explained by literacy increases understanding of reproductive and sexual health through reading printed materials and contraceptive utilization [20]. Teenagers who are illiterate will have a lower self-esteem and want to be accepted and get validation by the society by early childbearing [44].

In the multivariable multilevel logistic regression; literacy, marital status, age of the girls, age of household head and being resident of Gambela region were significantly associated with teenage pregnancy among adolescents aged 15 to 19. Literate teenagers have lower risk of teenage pregnancy compared to the illiterate ones. This finding is in line with studies conducted in Ethiopia [20], Zambia [45], Philadelphia; USA [46] and Africa [47]. Giving an emphasis on female literacy will reduce teenage pregnancy. Teenagers who were married are more likely to experience teenage pregnancy than those who are single. This is supported by previously conducted similar literatures in Ethiopia [20,41,48], Zambia [45], Uganda [49] and Nigeria [50]. As the frequency of sexually activities are higher among married teenagers, there is greater likelihood of pregnancy in the absence of contraception than those who are not married [51].

Regards to age, teenagers aged 17, 18 and 19 had higher risk of experiencing teenage pregnancy as compared to those who are 15 years old. This is similar with studies conducted in Ethiopia [20] and Kenya [52]. This is because as age increases the probability of getting sexual intercourse and marriage, which are proximal determinants of fertility, increases. Consequently, the chance of pregnancy and childbearing will also be increased [53]. As the age of household head increases, the chance of teenage pregnancy will be reduced. This is consistent with a study from Rwanda

[54]. This is because when household heads are older and more mature; they support teens under their household by giving them good parental support including advising their teen girls on risky sex and fulfilling financial needs [55,56]. In terms of region, the odds of experiencing teenage pregnancy among adolescents aged 15 to 19, who lived in Gambela region was higher as compared to the Tigray region. This is attributable to the region's high unmet need for family planning, higher polygamy practice, early sexual initiation among adolescents which exacerbates the condition [57].

The study's key strength was utilization of the most recent weighted nationally representative data with relatively high sample size. To generalize findings at the national level by adjusting the data's hierarchical nature, a multilevel model analysis was fitted. The use of spatial and multi-scale geographically weighted regression analysis assists to identify factors which contribute to the spatial variation of teenage pregnancy. This research, however has certain limitations that must be considered when interpreting findings. Important variables such as age at first sex and marriage and demand to family planning were not included since the mini-EDHS 2019. In addition to this, because of the cross sectional nature of EDHS data, the cause-effect relationship between the dependent and independent variables cannot be shown.

Conclusion

There is a significant spatial variation of teenage pregnancy among adolescents aged 15 to 19. The proportion of being illiterate and the proportion of being married had a significant positive association with teenage pregnancy throughout the country in MGWR model. In the multivariable multilevel logistic regression; literacy, marital status, age of the girls, age of household head and being resident of Gambela region were significantly associated with the outcome variable. To

address this issue, stronger actions must be taken on implementation of the law raising the minimum age for marriage to 18 years. The main cause of teenage pregnancy in Ethiopia, teenage marriage should be changed by addressing cultural norms which prioritize maintaining family status in the community.

Priority should also be given on enhancing levels of literacy. In addition to increasing girls' enrollment in formal schools, the government of Ethiopia must increase adult education programs for out of school young women by incorporating reproductive health issues in the program. The population must also get an awareness on the consequences and adverse outcomes of teenage pregnancy. Generally policies and implementations which focus on preventing early marriage, educating teenage girls and creating an awareness on reproductive health issues needs to be strengthen.

List of Abbreviations

AICc: Corrected Akaike Information Criteria

AOR: Adjusted Odds Ratio

CI: Confidence Interval

EAs: Enumeration Areas

EDHS: Ethiopian Demographic and Health Survey

GWR: Geographic Weighted Regression

HHH: Household Head

ICC: Intra-class Correlation Coefficient

LMIC: Low and Middle Income Countries

MOR: Median Odds Ratio

MGWR: Multi-Scale Geographically Weighted Regression

PCV: Proportional Change in Variance

RSS: Residual Sum of Squares

OLS: Ordinary Least Square

SSA: Sub-Saharan Africa

SNNPR: Southern Nations Nationalities and Peoples Region

Declarations

Ethical approval and consent to participate

The data was obtained from the Demographic and Health Surveys Program and can be freely accessed from the program website (www.dhsprogram.com). As the study was a secondary data analysis of publicly available data from the MEASURE DHS program, ethical approval and participant consent were not necessary for this particular study.

Consent for publication

Not applicable

Availability of data and materials

Data is available from http://www.dhsprogram.com

Competing interests

The authors declare they have no competing interests

Funding

The authors received no funding

Authors' contributions

Conceptualization: Tsion Mulat Tebeje, Mesfin Abebe, Ever Siyoum Shewarega, Gizaw Sisay, Beminate Lemma Seifu, Kusse Urmale Mare, Fantu Mamo Aragaw, Binyam Tariku Seboka

Data curation: Tsion Mulat Tebeje, Mesfin Abebe, Ever Siyoum Shewarega, Gizaw Sisay, Beminate Lemma Seifu, Kusse Urmale Mare, Fantu Mamo Aragaw, Binyam Tariku Seboka

Formal analysis: Tsion Mulat Tebeje, Mesfin Abebe, Ever Siyoum Shewarega, Gizaw Sisay, Beminate Lemma Seifu, Kusse Urmale Mare, Fantu Mamo Aragaw, Binyam Tariku Seboka

Investigation: Tsion Mulat Tebeje, Mesfin Abebe, Ever Siyoum Shewarega, Gizaw Sisay, Beminate Lemma Seifu, Kusse Urmale Mare, Fantu Mamo Aragaw, Binyam Tariku Seboka

Methodology: Tsion Mulat Tebeje, Mesfin Abebe, Ever Siyoum Shewarega, Gizaw Sisay, Beminate Lemma Seifu, Kusse Urmale Mare, Fantu Mamo Aragaw, Binyam Tariku Seboka

Software: Tsion Mulat Tebeje, Mesfin Abebe, Ever Siyoum Shewarega, Gizaw Sisay, Beminate Lemma Seifu, Kusse Urmale Mare, Fantu Mamo Aragaw, Binyam Tariku Seboka

Validation: Tsion Mulat Tebeje, Mesfin Abebe, Ever Siyoum Shewarega, Gizaw Sisay, Beminate Lemma Seifu, Kusse Urmale Mare, Fantu Mamo Aragaw, Binyam Tariku Seboka

Visualization: Tsion Mulat Tebeje, Mesfin Abebe, Ever Siyoum Shewarega, Gizaw Sisay, Beminate Lemma Seifu, Kusse Urmale Mare, Fantu Mamo Aragaw, Binyam Tariku Seboka

Writing original draft: Tsion Mulat Tebeje, Mesfin Abebe, Ever Siyoum Shewarega, Gizaw Sisay, Beminate Lemma Seifu, Kusse Urmale Mare, Fantu Mamo Aragaw, Binyam Tariku Seboka

Writing – review & editing: Tsion Mulat Tebeje, Mesfin Abebe, Ever Siyoum Shewarega, Gizaw Sisay, Beminate Lemma Seifu, Kusse Urmale Mare, Fantu Mamo Aragaw, Binyam Tariku Seboka

Acknowledgements

We would like to acknowledge the measure DHS program for providing the dataset to use the mini-EDHS 2019.

References

1. Kothari MT, Wang S, Head SK, Abderrahim N. Trends in adolescent reproductive and sexual behaviors [Internet]. Calverton, Maryland: ICF International; 2012. Available from: https://www.dhsprogram.com/pubs/pdf/CR29/CR29.pdf

2. Nove A, Matthews Z, Neal S, Camacho AV. Maternal mortality in adolescents compared with women of other ages: evidence from 144 countries. Lancet Glob Heal [Internet]. 2014 Mar 1;2(3):e155–64. Available from: https://doi.org/10.1016/S2214-109X(13)70179-7

3. Blanc AK, Winfrey W, Ross J. New Findings for Maternal Mortality Age Patterns: Aggregated Results for 38 Countries. PLoS One [Internet]. 2013 Apr 16;8(4):e59864. Available from: https://doi.org/10.1371/journal.pone.0059864

4. World Health Organization. Trends in maternal mortality 2000 to 2020: estimates by

WHO, UNICEF, UNFPA, World Bank Group and UNDESA/Population Division. Geneva; 2023.

5. Council NR, Population C on. Growing up global: The changing transitions to adulthood in developing countries. National Academies Press; 2005.

6. UNICEF. Progress for children: A report card on adolescents. New York UNICEF. 2012;(10).

7. Darroch JE, Woog V, Bankole A, Ashford LS. Adding it up: costs and benefits of meeting the contraceptive needs of adolescents. 2016.

8. Sully EA, Biddlecom A, Darroch JE, Riley T, Ashford LS, Lince-Deroche N, et al. Adding it up: investing in sexual and reproductive health 2019. 2020.

9. Shah IH, Åhman E. Unsafe abortion differentials in 2008 by age and developing country region: high burden among young women. Reprod Health Matters. 2012;20(39):169–73.

10. World Health Organization. SDG Target 3.7 Sexual and reproductive health [Internet]. Available from: https://www.who.int/data/gho/data/themes/topics/sdg-target-3_7-sexual-and-reproductive-health

11. Noori N, Proctor JL, Efevbera Y, Oron AP. The Effect of Adolescent Pregnancy on Child Mortality in 46 Low- and Middle-Income Countries. BMJ Glob Heal [Internet]. 2022 May 1;7(5):e007681. Available from: http://gh.bmj.com/content/7/5/e007681.abstract

12. Erulkar A, Ferede A, Ambelu W, Girma W, Amdemikael H, GebreMedhin B, et al. Ethiopia young adult survey: A study in seven regions. 2010;

13. Central Statistical Authority/Ethiopia, ORC Macro. Ethiopia Demographic and Health

Survey 2000 [Internet]. Addis Ababa, Ethiopia: Central Statistical Authority/Ethiopia and ORC Macro; 2001. Available from: http://dhsprogram.com/pubs/pdf/FR118/FR118.pdf

14. Central Statistical Agency/Ethiopia, ORC Marco. Ethiopia Demographic and Health Survey 2005 [Internet]. Addis Ababa, Ethiopia: Central Statistical Agency/Ethiopia and ORC Macro; 2006. Available from: http://dhsprogram.com/pubs/pdf/FR179/FR179.pdf

15. Central Statistical Agency/Ethiopia, ICF International. Ethiopia Demographic and Health Survey 2011 [Internet]. Addis Ababa, Ethiopia: Central Statistical Agency/Ethiopia and ICF International; 2012. Available from: http://dhsprogram.com/pubs/pdf/FR255/FR255.pdf

16. Central Statistical Agency - CSA/Ethiopia, ICF. Ethiopia Demographic and Health Survey 2016 [Internet]. Addis Ababa, Ethiopia: CSA and ICF; 2017. Available from: http://dhsprogram.com/pubs/pdf/FR328/FR328.pdf

17. Kaphagawani NC, Kalipeni E. Sociocultural factors contributing to teenage pregnancy in Zomba district, Malawi. Glob Public Health [Internet]. 2017 Jun 3;12(6):694–710. Available from: https://doi.org/10.1080/17441692.2016.1229354

18. World Health Organization. Adolescent pregnancy [Internet]. 2023. Available from: https://www.who.int/news-room/fact-sheets/detail/adolescent-pregnancy

19. Nuru Nabwire O. The association between the level of education and teenage pregnancy in Kenya A secondary analysis of Kenya Demographic and Health Survey 2014. 2023.

20. Bitew DA, Akalu Y, Belsti Y, Diress M, Gela YY, Belay DG, et al. Predictors of underage pregnancy among women aged 15–19 in highly prevalent regions of Ethiopia: a

multilevel analysis based on EDHS, 2016. Sci Rep [Internet]. 2023;13(1):857. Available from: https://doi.org/10.1038/s41598-023-27805-y

21. Imamura M, Tucker J, Hannaford P, Da Silva MO, Astin M, Wyness L, et al. Factors associated with teenage pregnancy in the European Union countries: a systematic review. Eur J Public Health. 2007;17(6):630–6.

22. Ayele BG, Gebregzabher TG, Hailu TT, Assefa BA. Determinants of teenage pregnancy in Degua Tembien District, Tigray, Northern Ethiopia: A community-based case-control study. PLoS One [Internet]. 2018 Jul 25;13(7):e0200898. Available from: https://doi.org/10.1371/journal.pone.0200898

23. Chang Chien YM, Carver S, Comber A. Using geographically weighted models to explore how crowdsourced landscape perceptions relate to landscape physical characteristics. Landsc Urban Plan [Internet]. 2020;203:103904. Available from: https://www.sciencedirect.com/science/article/pii/S0169204620304667

24. Gao Y, Zhao J, Han L. Exploring the spatial heterogeneity of urban heat island effect and its relationship to block morphology with the geographically weighted regression model. Sustain Cities Soc [Internet]. 2022;76:103431. Available from: https://www.sciencedirect.com/science/article/pii/S2210670721007046

25. Fotheringham AS, Yu H, Wolf LJ, Oshan TM, Li Z. On the notion of 'bandwidth' in geographically weighted regression models of spatially varying processes. Int J Geogr Inf Sci [Internet]. 2022 Aug 3;36(8):1485–502. Available from: https://doi.org/10.1080/13658816.2022.2034829

26. Ethiopian Public Health Institute - EPHI, Federal Ministry of Health - FMoH, ICF.

Ethiopia Mini Demographic and Health Survey 2019 [Internet]. Addis Ababa, Ethiopia: EPHI/FMoH/ICF; 2021. Available from: https://www.dhsprogram.com/pubs/pdf/FR363/FR363.pdf

27. World Population Review. Countries in Africa [Internet]. 2023. Available from: https://worldpopulationreview.com/country-rankings/countries-in-africa

28. Croft TN, Marshall AMJ, Allen CK, Arnold F, Assaf S, Balian S. Guide to DHS statistics. Rockv ICF. 2018;645.

29. Merlo J, Chaix B, Ohlsson H, Beckman A, Johnell K, Hjerpe P, et al. A brief conceptual tutorial of multilevel analysis in social epidemiology: using measures of clustering in multilevel logistic regression to investigate contextual phenomena. J Epidemiol Community Health. 2006 Apr;60(4):290–7.

30. Merlo J, Yang M, Chaix B, Lynch J, Råstam L. A brief conceptual tutorial on multilevel analysis in social epidemiology: investigating contextual phenomena in different groups of people. J Epidemiol Community Health [Internet]. 2005 Sep 1;59(9):729 LP – 736. Available from: http://jech.bmj.com/content/59/9/729.abstract

31. Chen Y. New Approaches for Calculating Moran's Index of Spatial Autocorrelation. PLoS One [Internet]. 2013 Jul 12;8(7):e68336. Available from: https://doi.org/10.1371/journal.pone.0068336

32. Zulu LC, Kalipeni E, Johannes E. Analyzing spatial clustering and the spatiotemporal nature and trends of HIV/AIDS prevalence using GIS: the case of Malawi, 1994-2010. BMC Infect Dis [Internet]. 2014;14(1):285. Available from: https://doi.org/10.1186/1471-2334-14-285

33. Esri. How Exploratory Regression works [Internet]. Available from: https://pro.arcgis.com/en/pro-app/latest/tool-reference/spatial-statistics/how-exploratory-regression-works.htm

34. Tareke AA, Gashu KD, Endehabtu BF. Geographical weighted regression analysis of delayed antenatal care initiation and its factors among all reproductive-aged women in Ethiopia, 2016. Contracept Reprod Med [Internet]. 2022;7(1):23. Available from: https://doi.org/10.1186/s40834-022-00190-z

35. Charlton M, Fotheringham AS, Brunsdon C. Geographically weighted regression white paper. Kildare Natl Univ Irel Maynooth. 2009;1–14.

36. Fotheringham AS, Yang W, Kang W. Multiscale Geographically Weighted Regression (MGWR). Ann Am Assoc Geogr [Internet]. 2017 Nov 2;107(6):1247–65. Available from: https://doi.org/10.1080/24694452.2017.1352480

37. Li Z, Fotheringham AS, Oshan TM, Wolf LJ. Measuring Bandwidth Uncertainty in Multiscale Geographically Weighted Regression Using Akaike Weights. Ann Am Assoc Geogr [Internet]. 2020 Sep 2;110(5):1500–20. Available from: https://doi.org/10.1080/24694452.2019.1704680

38. Lakew Y, Reda AA, Tamene H, Benedict S, Deribe K. Geographical variation and factors influencing modern contraceptive use among married women in Ethiopia: evidence from a national population based survey. Reprod Health [Internet]. 2013;10(1):52. Available from: https://doi.org/10.1186/1742-4755-10-52

39. van der Kwaak A, Baltissen G, Plummer D, Ferris K, Nduba J. Understanding nomadic realities: case studies on sexual and reproductive health and rights in Eastern Africa. KIT

Publishers; 2012.

40. Amegbor PM, Zhang Z, Dalgaard R, Sabel CE. Multilevel and spatial analyses of childhood malnutrition in Uganda: examining individual and contextual factors. Sci Rep [Internet]. 2020;10(1):20019. Available from: https://doi.org/10.1038/s41598-020-76856-y

41. Kassa GM, Arowojolu AO, Odukogbe ATA, Yalew AW. Trends and determinants of teenage childbearing in Ethiopia: evidence from the 2000 to 2016 demographic and health surveys. Ital J Pediatr [Internet]. 2019;45(1):153. Available from: https://doi.org/10.1186/s13052-019-0745-4

42. Ketema H, Erulkar A. Married Adolescents and Family Planning in Rural Ethiopia: Understanding Barriers and Opportunities. Afr J Reprod Health. 2018 Dec;22(4):26–34.

43. Chuta N. Neglected experiences: Fertility and childbearing among young people in Ethiopia. 2021;

44. Reading Partners. Early Reading Deficiencies Create Conditions that Enhance Teen Pregnancy Risk [Internet]. 2013. Available from: https://readingpartners.org/blog/early-reading-deficiencies-creating-conditions-that-enhance-teen-pregnancy/

45. Phiri M, Kasonde ME, Moyo N, Sikaluzwe M, Simona S. A multilevel analysis of trends and predictors associated with teenage pregnancy in Zambia (2001–2018). Reprod Health [Internet]. 2023;20(1):16. Available from: https://doi.org/10.1186/s12978-023-01567-2

46. Bennett IM, Frasso R, Bellamy SL, Wortham S, Gross KS. Pre-teen literacy and subsequent teenage childbearing in a US population. Contraception [Internet]. 2013 Apr

1;87(4):459–64. Available from: https://doi.org/10.1016/j.contraception.2012.08.020

47. Odejimi O, Bellingham-Young D. Teenage pregnancy in Africa: trends and determinants in the 21st century. J Heal Soc Care Improv 2016 Vol 1 issue 1 [Internet]. 2016;1(1):12–20. Available from: https://wlv.openrepository.com/bitstream/2436/620300/1/Teenage Pregnancy in Africa- Trend and Determinants in the 21st Century.pdf

48. Kefale B, Yalew M, Damtie Y, Adane B. A Multilevel Analysis of Factors Associated with Teenage Pregnancy in Ethiopia. Int J Womens Health. 2020;12:785–93.

49. Ochen AM, Chi PC, Lawoko S. Predictors of teenage pregnancy among girls aged 13–19 years in Uganda: a community based case-control study. BMC Pregnancy Childbirth [Internet]. 2019;19(1):211. Available from: https://doi.org/10.1186/s12884-019-2347-y

50. Ayoade MA. Spatiotemporal Patterns of Teenage Pregnancy in Nigeria: Evidence from the 2008, 2013 and 2018 NDHS. Pap Appl Geogr [Internet]. 2021 Apr 3;7(2):161–82. Available from: https://doi.org/10.1080/23754931.2020.1852103

51. World Health Organization. Early marriages, adolescent and young pregnancies. Geneva WHO. 2012;1–4.

52. Were M. Determinants of teenage pregnancies: the case of Busia District in Kenya. Econ Hum Biol. 2007 Jul;5(2):322–39.

53. Alemayehu T, Haider J, Habte D. Determinants of adolescent fertility in Ethiopia. Ethiop J Heal Dev. 2010;24(1).

54. Uwizeye D, Muhayiteto R, Kantarama E, Wiehler S, Murangwa Y. Prevalence of teenage pregnancy and the associated contextual correlates in Rwanda. Heliyon. 2020

Oct;6(10):e05037.

55. Nwogwugwu NC. Socio-demographic determinants of adolescent fertility in Zambia. University of the Witwatersrand, Faculty of Humanities; 2013.

56. Domenico DM, Jones KH. Adolescent pregnancy in America: Causes and responses. J Vocat Spec Needs Educ. 2007;30(1):4–12.

57. Bol KN, Negera E, Gedefa AG. Pregnancy among adolescent girls in humanitarian settings: a case in refugee camp of Gambella regional state, community-based cross-sectional study, Southwest Ethiopia, 2021. BMJ Open [Internet]. 2022 Nov 1;12(11):e064732. Available from: http://bmjopen.bmj.com/content/12/11/e064732.abstract

Legend

List of Figures

Figure 6: The global spatial autocorrelation analysis of teenage pregnancy among adolescents aged 15 to 19 in Ethiopia, 2019

Figure 7: The Getis Ord Gi statistical analysis of hotspots of teenage pregnancy among adolescents aged 15 to 19 in Ethiopia, 2019

Figure 8: GWR (left) and MGWR (right) parameter estimates for the intercept to show local patterns of spatial heterogenity. Grey dots are not statistically different from zero

Figure 9: GWR (left) and MGWR (right) parameter estimates for the proportion of married to show local patterns of spatial heterogeneity. Grey dots are not statistically different from zero

Figure 10: GWR (left) and MGWR (right) parameter estimates for the proportion of illiterate to show local patterns of spatial heterogeneity. Grey dots are not statistically different from zero

Figure

Click here to access/download;Figure;Figure 1.JPG

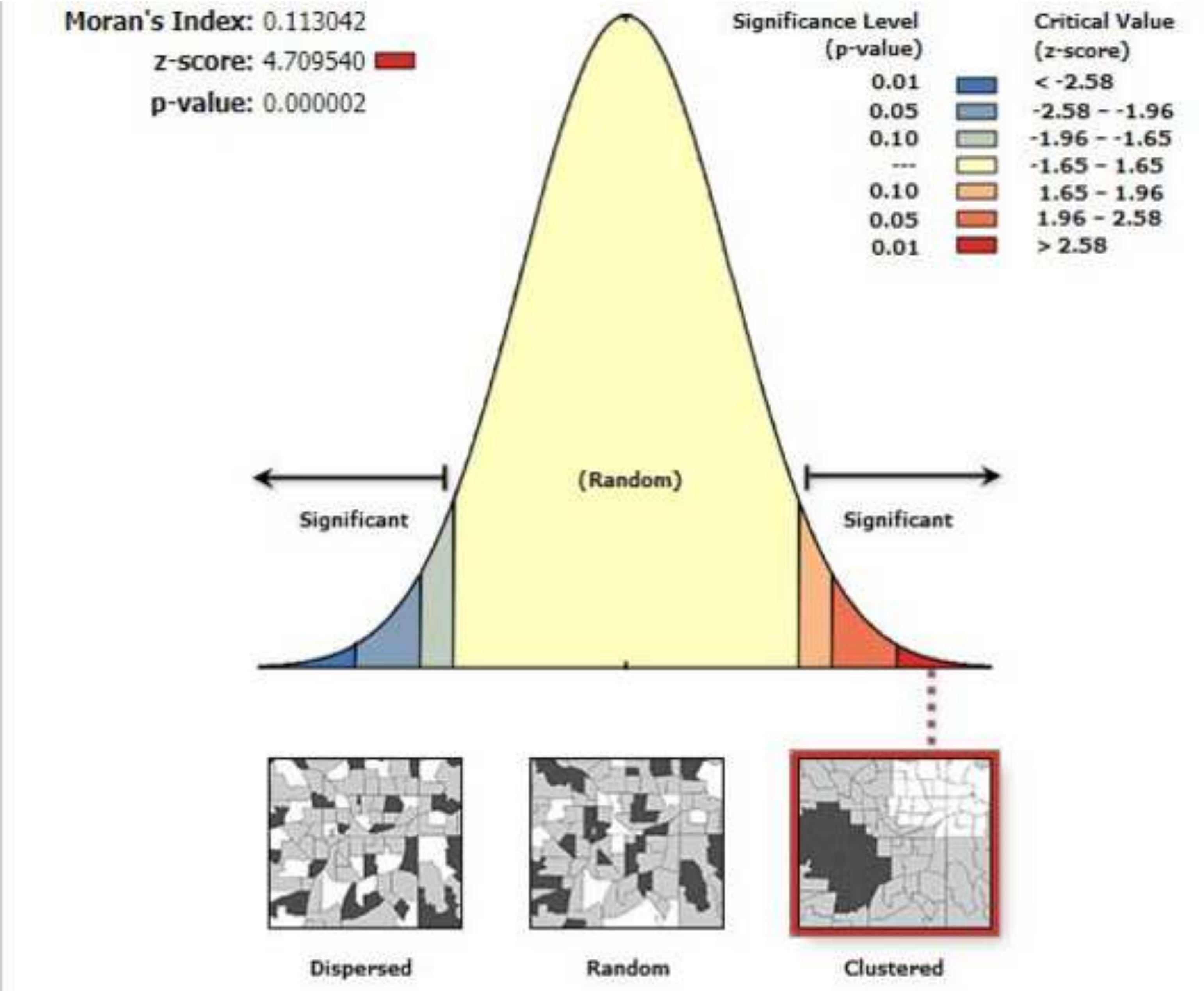

Given the z-score of 4.70954028255, there is a less than 1% likelihood that this clustered pattern could be the result of random chance.

Figure

Click here to access/download;Figure;Figure 2.pdf

Hotspot analysis of teenage pregnancy in Ethiopia, 2019

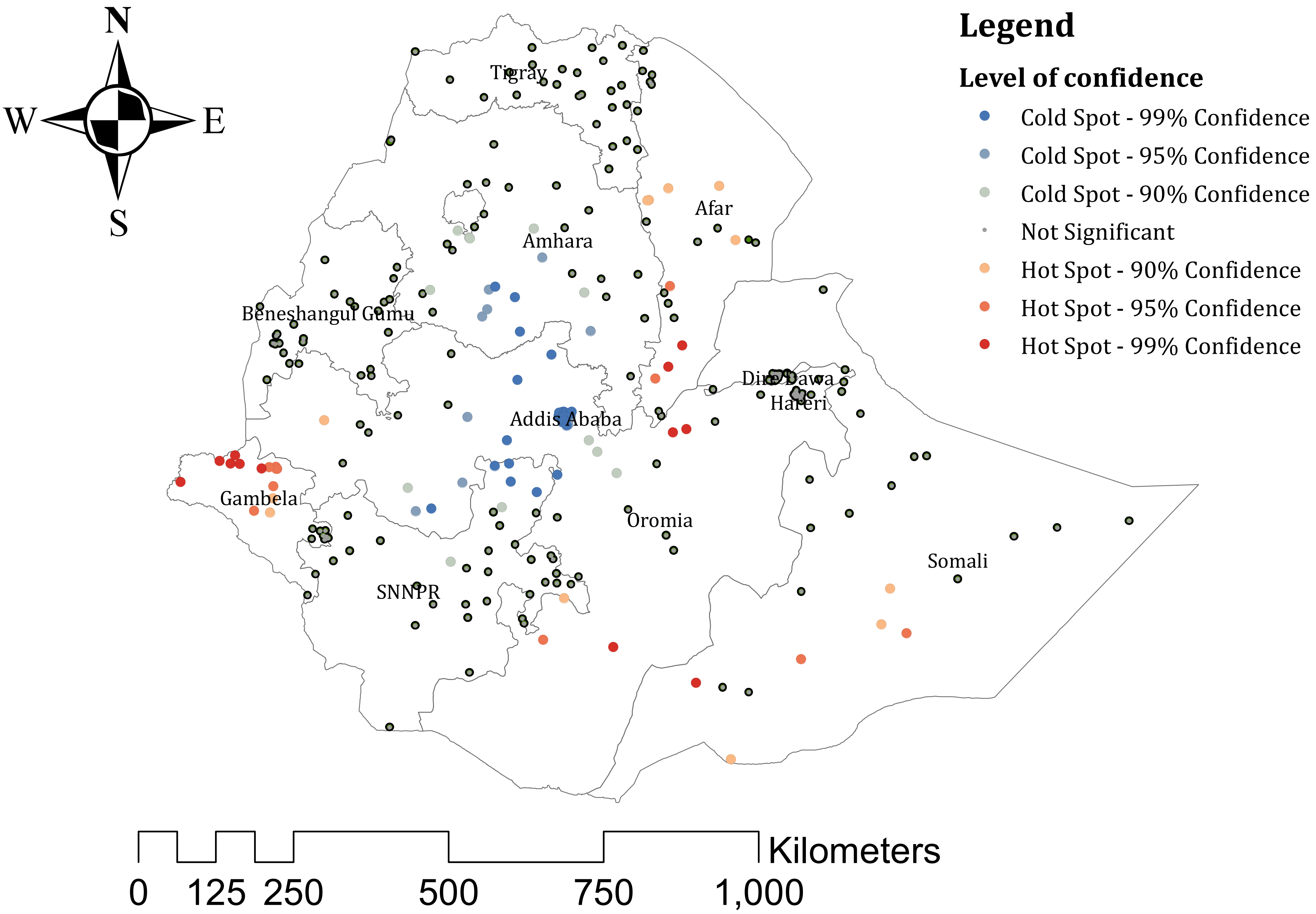

Figure

Click here to access/download;Figure;Figure 3.pdf

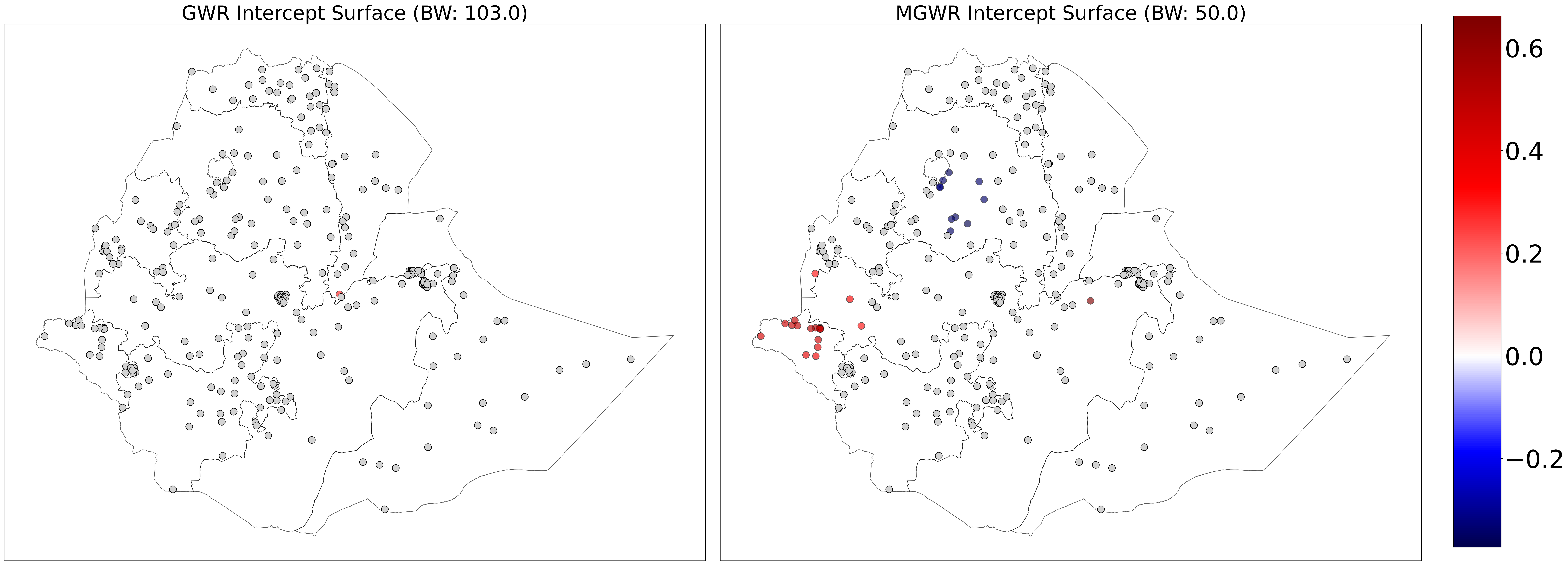

Click here to access/download;Figure;Figure 4.pdf

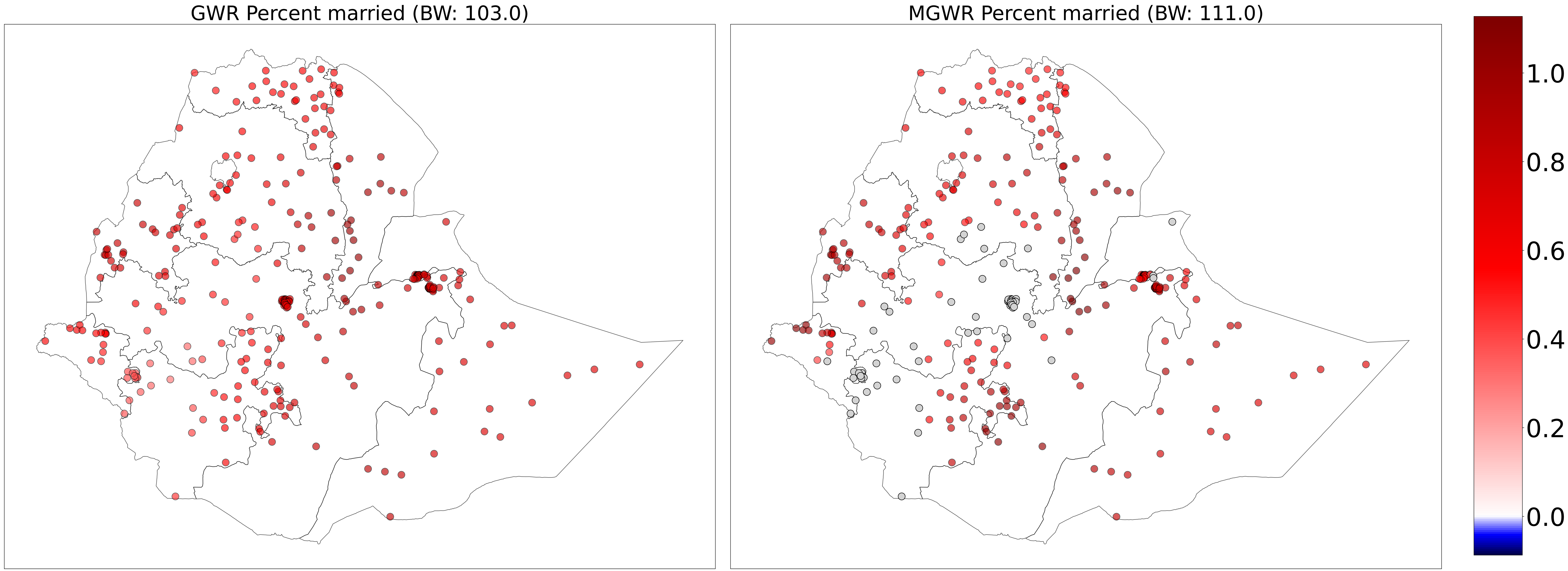

Click here to access/download;Figure;Figure 5.pdf

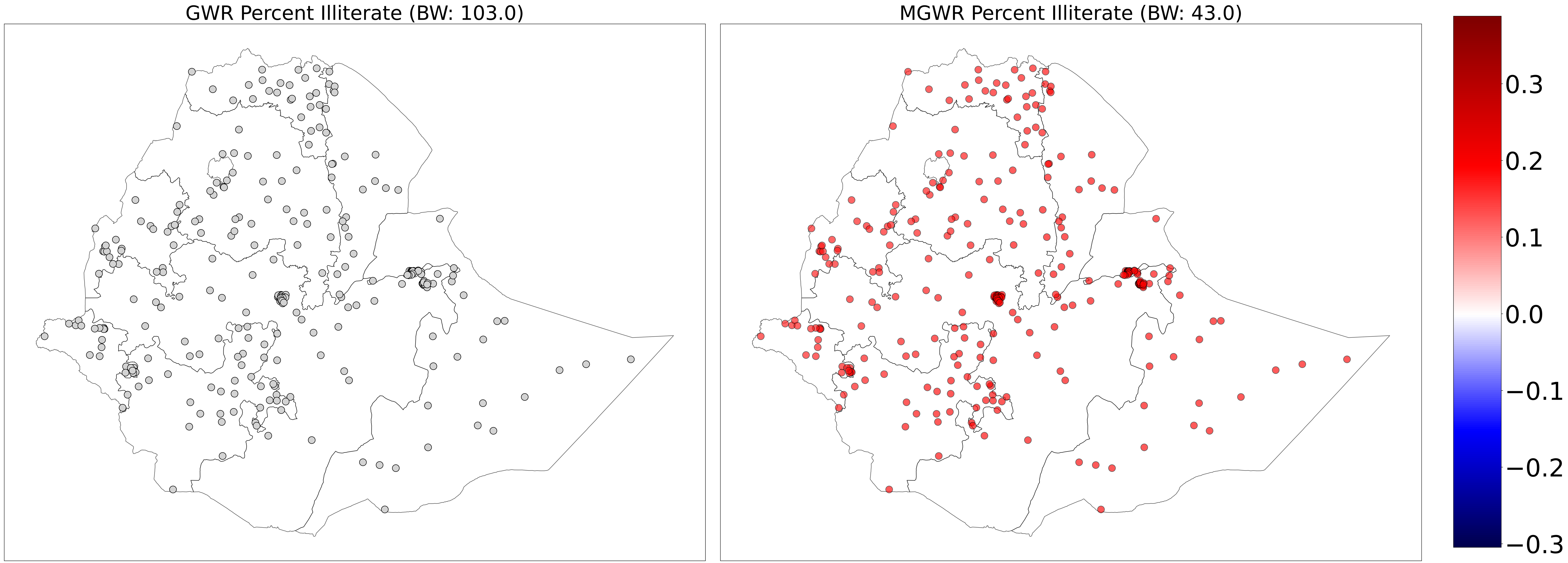

Supplement: Attachment — Submitted filename: PONE-D-23-27072-AF.pdf [file pone.0310025.s001.pdf]
