# Supplementary material for: A multiscale geographically weighted regression analysis of teenage pregnancy and associated factors among adolescents aged 15 to 19 in Ethiopia using the 2019 mini-demographic and health survey
Source: PLoS One. 2024 Sep 6;19(9):e0310025. doi: 10.1371/journal.pone.0310025 (PMC11379268; doi:10.1371/journal.pone.0310025)
Supplement: Attachment — Submitted filename: PONE-D-23-27072_R1-AF.pdf [file pone.0310025.s004.pdf]

PLOS ONE

# A multiscale geographically weighted regression analysis of teenage pregnancy and associated factors among adolescents aged 15 to 19 in Ethiopia using the 2019 mini-demographic and health survey

--Manuscript Draft--

| | |
|---|---|
| Manuscript Number: | PONE-D-23-27072R1 |
| Article Type: | Research Article |
| Full Title: | A multiscale geographically weighted regression analysis of teenage pregnancy and associated factors among adolescents aged 15 to 19 in Ethiopia using the 2019 mini-demographic and health survey |
| Short Title: | Determinants of teenage pregnancy, a multilevel and MGWR analysis |
| Corresponding Author: | Tsion Mulat Tebeje, MPH<br>Dilla University<br>Dilla, ETHIOPIA |
| Keywords: | Adolescents, Demographic and health survey, Ethiopia, Teenage pregnancy, MGWR |
| Abstract: | Background: Teenage pregnancy remains one of the major reproductive health problems, especially in sub-Saharan African countries. It can lead to maternal and neonatal complications and social consequences. The proportion of teenage pregnancy differs across regions of Ethiopia. Thus, this study aimed to determine the spatial variation in determinants of teenage pregnancy among adolescents aged 15-19 years in Ethiopia using the 2019 Demographic and Health Survey (DHS).<br>Methods: This study included a total weighted sample of 2165 teenage girls aged 15 to 19 years. A mixed-effect binary logistic regression model was employed to consider the hierarchical nature of the DHS data using STATA version 17. Adjusted odds ratios with 95% confidence intervals are reported, and a p-value less than 0.05 was used to identify significant predictors. The spatial analysis was conducted with ArcGIS version 10.7 and Python 3. To identify factors associated with the hotspots of teenage pregnancy, a multiscale geographically weighted regression was performed. Spatial regression models were compared using adjusted R2, the corrected Akaike information criterion (AICc), and the residual sum of squares (RSS).<br>Results: The prevalence of teenage pregnancy among adolescents aged 15 to 19 years was 12.98% (95% CI: 11.6%, 14.5%). It was spatially clustered throughout the country with a significant Moran's I value. Significant hotspot areas were detected in central and southern Afar; northern, central, and western Gambela; northeastern and southern central Oromia; and the eastern Somali region. The MGWR analysis revealed that the significant predictors of spatial variations in teenage pregnancy were being illiterate and being married. According to the multivariable multilevel analysis, age 17 (AOR = 3.54; 95% CI: 1.60, 7.81), 18 (AOR = 8.21; 95% CI: 3.96, 17.0), 19 (AOR = 15.0; 95% CI: 6.84, 32.9), being literate (AOR = 0.57; 95% CI: 0.35, 0.92), being married (AOR = 22.8; 95% CI: 14.1, 37.0), age of household head (AOR = 0.98; 95% CI: 0.98, 0.99) and residence in the Gambela region (AOR = 3.27; 95% CI: 1.21, 8.86) were significantly associated with teenage pregnancy among adolescents aged 15 to 19.<br>Conclusion: Teenage pregnancy is a public health problem in Ethiopia. Policymakers should prioritize addressing early marriage and improving teenage literacy rates, with a focus on the Gambela region and other hotspot areas. It is crucial to implement policies aimed at transforming the traditional practice of early marriage and to take measures to enhance literacy levels and promote awareness about reproductive health. This will help ensure that young people have the opportunity to pursue education and make informed decisions about their reproductive health. |
| Order of Authors: | Tsion Mulat Tebeje, MPH |
| | Mesfin Abebe, MSc |
| | Fantu Mamo Aragaw, MPH |
| | Beminate Lemma Seifu, MPH |
| | |

Powered by Editorial Manager® and ProduXion Manager® from Aries Systems Corporation

| | |
|---|---|
| | Kusse Urmale Mare, MSc |
| | Ever Siyoum Shewarega, MPH |
| | Gizaw Sisay, MSc |
| | Binyam Tariku Seboka, MPH |
| **Opposed Reviewers:** | |
| **Response to Reviewers:** | Subject: Responses to academic editor's comments<br>Dear Dr. Alfredo Luis Fort,<br>Thank you for taking the time to consider our manuscript titled “A multiscale geographically weighted regression analysis of teenage pregnancy and associated factors among adolescents aged 15 to 19 in Ethiopia using the 2019 mini-demographic and health survey” for the Plos One Journal original research article. We appreciate the time and effort you have dedicated to providing valuable feedback on our manuscript. We have taken the comments and concerns into account and made every effort to address them. We agree with all the comments and have incorporated the corresponding revisions into the revised manuscript. We believe that our manuscript has been significantly improved as a result of these revisions, and we hope that our revised manuscript is acceptable for publication in the PLOS One journal.<br>We would like to thank you once again for your consideration of our work and for inviting us to submit the revised manuscript. We look forward to hearing from you. Our detailed, point-by-point responses to the comments are given below.<br><br>Best regards,<br>Tsion Mulat Tebeje<br>School of Public Health, Dilla University, Dilla, Ethiopia.<br>Email: yemarina12@gmail.com (corresponding author)<br><br><br><br>Response to journal’s requirements<br>1.Please ensure that your manuscript meets PLOS ONE's style requirements, including those for file naming.<br>Response: We prepared the manuscript according to the journal's requirements and double-checked its compliance before submitting our revised manuscript.<br>2.Consider depositing your raw data in a repository to ensure your work is read, appreciated and cited by the largest possible audience. You’ll also earn an Accessible Data icon on your published paper if you deposit your data in any participating repository (https://plos.org/open-science/open-data/#accessible-data).<br>Response: Thank you for your suggestion. We have included it in the revised manuscript and highlighted it in the 'Data Availability' section, specifically on lines 417-418, page 25.<br>3.Please provide additional details regarding participant consent. In the ethics statement in the Methods and online submission information, please ensure that you have specified what type you obtained (for instance, written or verbal, and if verbal, how it was documented and witnessed). If your study included minors, state whether you obtained consent from parents or guardians. If the need for consent was waived by the ethics committee, please include this information.<br>Response: This study involved a secondary data analysis using publicly available data from the MEASURE DHS program and ethical approval and participant consent was not required. This is highlighted in the ethical approval and consent to participate section (lines 194-196, page 9). The data files do not contain any names of individuals or household addresses. We obtained approval from the DHS program to use the dataset and have attached the proof of granted permission as an “other” file with our submission.<br>4.Your ethics statement should only appear in the Methods section of your manuscript. If your ethics statement is written in any section besides the Methods, please move it to the Methods section and delete it from any other section. Please ensure that your ethics statement is included in your manuscript, as the ethics statement entered into the online submission form will not be published alongside your manuscript.<br>Response: Thank you for the suggestion. We have moved the ethics statement to the methods section in the revised manuscript.<br>5.We note that Figures 2,3,4 and 5 in your submission contain [map/satellite] images |

which may be copyrighted. All PLOS content is published under the Creative Commons Attribution License (CC BY 4.0), which means that the manuscript, images, and Supporting Information files will be freely available online, and any third party is permitted to access, download, copy, distribute, and use these materials in any way, even commercially, with proper attribution. For these reasons, we cannot publish previously copyrighted maps or satellite images created using proprietary data, such as Google software (Google Maps, Street View, and Earth).
We require you to either (1) present written permission from the copyright holder to publish these figures specifically under the CC BY 4.0 license, or (2) remove the figures from your submission:
You may seek permission from the original copyright holder of Figures 2,3,4 and 5 to publish the content specifically under the CC BY 4.0 license.
Response: We appreciate your concern regarding the ethical issues. However, the figures (2, 3, 4, and 5) mentioned in our manuscript are not copyrighted; rather, they are the results of spatial analysis conducted using ArcGIS and Python software. The DHS geographic information system, which contains shapefiles with coordinates (latitude and longitude), were obtained from the DHS office by explaining the objective of the study through online requests. To obtain these figures, we imported relevant data extracted from the 2019 Ethiopian mini Demographic and Health Survey. We have attached the permission letter obtained from MEASURE DHS to use the EDHS dataset and geographical location as an “other” file labeled 'AuthLetter_188507'. The Ethiopian administrative boundaries shapefile was obtained from the Ethiopian Central Statistical Agency (CSA). The shape file used to construct the figures can be accessed publicly at https://africaopendata.org/dataset/ethiopia-shapefiles. We incorporated and highlighted this information in captions of figures 2, 3, 4, and 5.
As a result, the maps presented in our study are not copyrighted. Instead, they are the findings of our spatial analysis, which we performed using ArcGIS and Python on shapefiles and projected CSV files of coordinates. This is the exact process that we followed when conducting our study. Therefore, we can confirm that the figures presented in our study represent our spatial analysis results and are not copyrighted.
Edits requested on the submission
1.We note your current data availability statement:
"The data used in this research are publicly available from the measure DHS program via an online request at http://www.dhsprogram.com"
As the URL presented in your Data Availability Statement and cover letter links to the DHS Program general website, please provide any direct URL(s), accession numbers, instructions, and additional information (DOI, data set title, etc.) required for researchers to access the specific data underlying the results presented in your study.
Response: Thank you for the suggestion. We have edited the data availability statement by providing the direct URL which links to the login or register for datasets. By registering and logging in, anyone can directly access the dataset.
2.Please clarify if the data used for the maps are copyrighted/what copyright information or restrictions are attached to it. If the data is copyrighted then please send the below permission form to the owners of the data.
Response: Thank you for asking for clarification. The maps included in our study are not copyrighted from somewhere. They were the output of our work created using ArcGIS version 10.7 and Python 3 software based on the maps on the Ethiopian shapefile obtained from the Ethiopian Central Statistical Agency (CSA), which is publicly available on Open-Africa https://africaopendata.org/dataset/ethiopia-shapefiles, and the GPS data obtained from the measure DHS program after submitting a request along with the study's rationale. Therefore, we assure you that the maps presented in this paper are the result of our work, utilizing appropriate analytical methods and procedures. Furthermore, we have acknowledged the source of the data, and we, as authors, have previously published studies containing similar maps.
Response to editor’s comments
1.I have seen a similar study like this, with the same problems. The topic and method plus results are of extreme importance. However, we cannot invite Reviewers because they will probably reject the manuscript because, like me, they will find lots of errors, misspellings, and inadequate descriptions. Please take the manuscript to someone who can write proper English, and use my first suggestions (in attached file) to continue improving the wording and descriptions for the rest of the file.
Response: Thank you for giving us your valuable time and for sharing your valuable input. We fully agree with your suggestion. We have thoroughly reviewed existing similar studies and have appropriately cited them. In the final paragraph of the

| | |
|---|---|
| | introduction section, we elucidated how our study differs. The revised manuscript has been meticulously edited and proofread to rectify quality issues, such as misspellings, grammatical errors, and unclear sentences. |
| **Additional Information:** | |
| **Question** | **Response** |
| **Financial Disclosure**<br><br>Enter a financial disclosure statement that describes the sources of funding for the work included in this submission. Review the submission guidelines for detailed requirements. View published research articles from *PLOS ONE* for specific examples.<br><br>This statement is required for submission and **will appear in the published article** if the submission is accepted. Please make sure it is accurate.<br><br>**Unfunded studies**<br>Enter: *The author(s) received no specific funding for this work.*<br><br>**Funded studies**<br>Enter a statement with the following details:<br>• Initials of the authors who received each award<br>• Grant numbers awarded to each author<br>• The full name of each funder<br>• URL of each funder website<br>• Did the sponsors or funders play any role in the study design, data collection and analysis, decision to publish, or preparation of the manuscript?<br>• **NO** - Include this sentence at the end of your statement: *The funders had no role in study design, data collection and analysis, decision to publish, or preparation of the manuscript.*<br>• **YES** - Specify the role(s) played.<br><br>* typeset | The author(s) received no specific funding for this work. |
| **Competing Interests**<br><br>Use the instructions below to enter a competing interest statement for this submission. On behalf of all authors, disclose any competing interests that could be perceived to bias this work—acknowledging all financial support | The authors have declared that no competing interests exist. |

| | |
|---|---|
| and any other relevant financial or non-financial competing interests.<br><br>This statement is required for submission and **will appear in the published article** if the submission is accepted. Please make sure it is accurate and that any funding sources listed in your Funding Information later in the submission form are also declared in your Financial Disclosure statement.<br><br>View published research articles from *PLOS ONE* for specific examples.<br><br>**NO authors have competing interests**<br><br>Enter: *The authors have declared that no competing interests exist.*<br><br>**Authors with competing interests**<br><br>Enter competing interest details beginning with this statement:<br><br>*I have read the journal's policy and the authors of this manuscript have the following competing interests: [insert competing interests here]*<br><br>* typeset | |
| **Ethics Statement**<br><br>Enter an ethics statement for this submission. This statement is required if the study involved:<br><br>• Human participants<br>• Human specimens or tissue<br>• Vertebrate animals or cephalopods<br>• Vertebrate embryos or tissues<br>• Field research<br><br>Write "N/A" if the submission does not require an ethics statement.<br><br>General guidance is provided below. | Data access permission was received from the measure DHS program via an online request at http://www.dhsprogram.com. The data used in this research were publicly available and did not contain any personal identifiers |

Powered by Editorial Manager® and ProduXion Manager® from Aries Systems Corporation

| | |
|---|---|
| Consult the submission guidelines for detailed instructions. **Make sure that all information entered here is included in the Methods section of the manuscript.**<br><br>**Format for specific study types**<br><br>**Human Subject Research (involving human participants and/or tissue)**<br>• Give the name of the institutional review board or ethics committee that approved the study<br>• Include the approval number and/or a statement indicating approval of this research<br>• Indicate the form of consent obtained (written/oral) or the reason that consent was not obtained (e.g. the data were analyzed anonymously)<br><br>**Animal Research (involving vertebrate animals, embryos or tissues)**<br>• Provide the name of the Institutional Animal Care and Use Committee (IACUC) or other relevant ethics board that reviewed the study protocol, and indicate whether they approved this research or granted a formal waiver of ethical approval<br>• Include an approval number if one was obtained<br>• If the study involved *non-human primates*, add *additional details* about animal welfare and steps taken to ameliorate suffering<br>• If anesthesia, euthanasia, or any kind of animal sacrifice is part of the study, include briefly which substances and/or methods were applied<br><br>**Field Research**<br><br>Include the following details if this study involves the collection of plant, animal, or other materials from a natural setting:<br>• Field permit number<br>• Name of the institution or relevant body that granted permission | |
| **Data Availability**<br><br>Authors are required to make all data underlying the findings described fully available, without restriction, and from the | Yes - all data are fully available without restriction |

Powered by Editorial Manager® and ProduXion Manager® from Aries Systems Corporation

| | |
|---|---|
| time of publication. PLOS allows rare exceptions to address legal and ethical concerns. See the PLOS Data Policy and FAQ for detailed information.<br><br>A Data Availability Statement describing where the data can be found is required at submission. Your answers to this question constitute the Data Availability Statement and **will be published in the article**, if accepted.<br><br>**Important:** Stating 'data available on request from the author' is not sufficient. If your data are only available upon request, select 'No' for the first question and explain your exceptional situation in the text box.<br><br>Do the authors confirm that all data underlying the findings described in their manuscript are fully available without restriction? | |
| **Describe where the data may be found in full sentences. If you are copying our sample text, replace any instances of XXX with the appropriate details.**<br><br>• If the data are **held or will be held in a public repository**, include URLs, accession numbers or DOIs. If this information will only be available after acceptance, indicate this by ticking the box below. For example: *All XXX files are available from the XXX database (accession number(s) XXX, XXX.*).<br>• If the data are all contained **within the manuscript and/or Supporting Information files**, enter the following: *All relevant data are within the manuscript and its Supporting Information files.*<br>• If neither of these applies but you are able to provide **details of access elsewhere**, with or without limitations, please do so. For example:<br><br>*Data cannot be shared publicly because of [XXX]. Data are available from the XXX Institutional Data Access / Ethics Committee (contact via XXX) for* | The data used in this research are publicly available from the measure DHS program via an online request at https://dhsprogram.com/data/dataset_admin/login_main.cfm?CFID=10818526&CFTOKEN=c131014a480fe56-4E0C6B7F-F551-E6B2-50 |

| | |
|---|---|
| *researchers who meet the criteria for access to confidential data.*<br><br>*The data underlying the results presented in the study are available from (include the name of the third party and contact information or URL).*<br>• This text is appropriate if the data are owned by a third party and authors do not have permission to share the data.<br><br>* typeset | |
| Additional data availability information: | |

Editor in Chief

PloS One

Date: 08/23/2023

Dear Editor,

I am writing to submit a manuscript entitled, "**A multiscale geographically weighted regression analysis of teenage pregnancy and associated factors among adolescents aged 15 to 19 in Ethiopia using the 2019 mini-demographic and health survey"**. We believe that our manuscript has been conducted precisely and hope that it will be accepted for publication in the PloS One Journal.

The study is intended to model spatial determinants of teenage pregnancy through the identification of geographic locations with a higher magnitude of the outcome and predict teenage pregnancy. The study was conducted to overcome the limitation of previous studies which utilized Geographic Weighted Regression for the relationship between the dependent and independent variables. Multiscale Geographic Weighted Regression considers multiple scales and relaxes the assumption of GWR by determining bandwidths that are parameter-specific. This allows the relationships between independent and dependent variables to vary at different spatial scales.

I would like to thank you once again for your consideration of our work. I look forward to hearing from you. With the submission, the authors confirm no conflict of interest and also confirm that the manuscript has not been published nor is under consideration else.

Thank you for your time and consideration.

Yours sincerely,

Tsion Mulat Tebeje

School of Public Health, College of Health Science and Medicine, Dilla University, Dilla, Ethiopia.

Email: yemarina12@gmail.com

Manuscript Click here to access/download;Manuscript;Manuscript.docx

# A multiscale geographically weighted regression analysis of teenage pregnancy and associated factors among adolescents aged 15 to 19 in Ethiopia using the 2019 mini-demographic and health survey

Tsion Mulat Tebeje[1*], Mesfin Abebe[2], Fantu Mamo Aragaw[3], Beminate Lemma Seifu[4], Kusse Urmale Mare[5], Ever Siyoum Shewarega[6], Gizaw Sisay[1], Binyam Tariku Seboka[1]

1. School of Public Health, College of Health Science and Medicine, Dilla University, Dilla, Ethiopia
2. Department of Midwifery, College of Health Science and Medicine, Dilla University, Dilla, Ethiopia
3. Department of Epidemiology and Biostatistics, Institute of Public Health, College of Medicine and Health Sciences, University of Gondar, Gondar, Ethiopia
4. Department of Public Health, College of Medicine and Health Sciences, Samara University, Afar, Ethiopia
5. Department of Nursing, College of Medicine and Health Sciences, Samara University, Afar, Ethiopia
6. Department of Reproductive Health, School of Public Health, College of Health Science and Medicine, Dilla University, Dilla, Ethiopia

*Corresponding author

Email: yemarina12@gmail.com (TMT)

# Abstract

**Background**: Teenage pregnancy remains one of the major reproductive health problems, especially in sub-Saharan African countries. It can lead to maternal and neonatal complications and social consequences. The proportion of teenage pregnancy differs across regions of Ethiopia. Thus, this study aimed to determine the spatial variation in determinants of teenage pregnancy among adolescents aged 15-19 years in Ethiopia using the 2019 Demographic and Health Survey (DHS).

**Methods**: This study included a total weighted sample of 2165 teenage girls aged 15 to 19 years. A mixed-effect binary logistic regression model was employed to consider the hierarchical nature of the DHS data using STATA version 17. Adjusted odds ratios with 95% confidence intervals are reported, and a p-value less than 0.05 was used to identify significant predictors. The spatial analysis was conducted with ArcGIS version 10.7 and Python 3. To identify factors associated with the hotspots of teenage pregnancy, a multiscale geographically weighted regression was performed. Spatial regression models were compared using adjusted R2, the corrected Akaike information criterion (AICc), and the residual sum of squares (RSS).

**Results**: The prevalence of teenage pregnancy among adolescents aged 15 to 19 years was 12.98% (95% CI: 11.6%, 14.5%). It was spatially clustered throughout the country with a significant Moran’s I value. Significant hotspot areas were detected in central and southern Afar; northern, central, and western Gambela; northeastern and southern central Oromia; and the eastern Somali region. The MGWR analysis revealed that the significant predictors of spatial variations in teenage pregnancy were being illiterate and being married. According to the multivariable multilevel analysis, age 17 (AOR = 3.54; 95% CI: 1.60, 7.81), 18 (AOR = 8.21; 95% CI: 3.96, 17.0), 19

(AOR = 15.0; 95% CI: 6.84, 32.9), being literate (AOR = 0.57; 95% CI: 0.35, 0.92), being married (AOR = 22.8; 95% CI: 14.1, 37.0), age of household head (AOR = 0.98; 95% CI: 0.98, 0.99) and residence in the Gambela region (AOR = 3.27; 95% CI: 1.21, 8.86) were significantly associated with teenage pregnancy among adolescents aged 15 to 19.

**Conclusion**: Teenage pregnancy is a public health problem in Ethiopia. Policymakers should prioritize addressing early marriage and improving teenage literacy rates, with a focus on the Gambela region and other hotspot areas. It is crucial to implement policies aimed at transforming the traditional practice of early marriage and to take measures to enhance literacy levels and promote awareness about reproductive health. This will help ensure that young people have the opportunity to pursue education and make informed decisions about their reproductive health.

**Keywords**: Adolescents, Demographic and health survey, Ethiopia, Teenage pregnancy, MGWR

# Introduction

In developing countries, the highest number of teenage childbirths occur, as most girls marry early in life [1]. Approximately 20 countries in sub-Saharan Africa and Asia are responsible for 82% of adolescent maternal mortality [2]. In comparison, girls aged 15 to 19 years have a 28% greater risk of maternal mortality than women aged 20 to 24 years [3]. Pregnancy-related conditions are a significant cause of death among adolescent girls aged 15-19 years worldwide [4]. Girls aged 15 to 19 years make up 11% of all births worldwide [5]. Although there has been a decline in the rate of adolescent births, the total number of births has not decreased as much due to the growing adolescent population [6]. Each year, adolescents from low- and middle-income countries experience an estimated 21 million pregnancies, resulting in 12 million births [7]. Half of these pregnancies are unintended, and 55% of the unintended pregnancies often end in unsafe abortions

[8]. Annually, 3.2 million unsafe abortions take place among adolescents aged 15 to 19, accounting for 15% of the total global incidence [9].

Adolescents have a significant unmet need for sexual and reproductive health care. A total of 43% of girls aged 15 to 19 years have an unmet need for modern contraception, which is higher than that of women of reproductive age, who have a 24% unmet need [7]. Adolescent pregnancies lead to health consequences such as increased risks of eclampsia, puerperal endometritis, and systemic infections. There are also social consequences, including stigma, rejection, violence by partners or parents, and dropping out of school, which are commonly observed [10]. Additionally, adolescent pregnancy is related to lower child survival, which is attributable to biological and social risks [11].

Teenage pregnancy is a significant public health issue in Ethiopia, where there is limited knowledge and access to reproductive health services [12]. The country ranks third in terms of adolescent maternal deaths [2]. Data from the 2000, 2005, 2011, and 2016 Ethiopian Demographic and Health Survey (EDHS) show that teenage pregnancy rates in Ethiopia were 16%, 17%, 12%, and 13%, respectively [13–16]. When broken down by region, the highest percentage was in the Afar region (23%), and the lowest percentage was in the capital, Addis Ababa (3%) [16].

According to previous research, factors related to adolescent pregnancy in LMICs include lower education and socioeconomic status, child marriage, not utilizing contraception, child sexual abuse and early sexual activity, age, religious beliefs, limited knowledge of reproductive and sexual health, gender inequality, physical or sexual violence, lower family income, being in the 18-19 age group, maternal history of teenage pregnancy, and inadequate communication with parents about reproductive health issues [17–22].

Fertility and childbearing among young people are among the most neglected issues in Ethiopia. Some similar studies have been conducted in the country [23–27], but they were based on the 2016 EDHS. Four of the aforementioned studies [23–26] did not take into account the geographical variation in teenage pregnancy in Ethiopia. However, one of them [27] examined regional disparities and studied the factors that influence regional disparities in teenage pregnancy using geographically weighted regression (GWR). Our study used the most recent data from the mini-EDHS 2019 to provide updated information. Additionally, this study was conducted to address the limitation of the previous study, which used GWR to assess the relationship between teenage pregnancy and its determinants. GWR assumes that the relationship between the outcome and its predictors operates at the same spatial scale. This disregards the fact that different relationships may occur at various scales due to heterogeneity or nonstationarity in geospatial relationships. To address this methodological gap, we used Multiscale Geographically Weighted Regression (MGWR), which considers multiple scales and relaxes the assumption of GWR by determining parameter-specific bandwidths. This allows the relationships between independent and dependent variables to vary at different spatial scales [28–30]. The purpose of this study was to better understand the spatial context of the determinants of teenage pregnancy using an explicit multiscale approach. Therefore, our study aimed to explore the spatial determinants of teenage pregnancy among adolescents aged 15 to 19 years using the most recent EDHS.

# Materials and Methods

A community-based cross-sectional study was conducted in Ethiopia by using mini-EDHS 2019. After Nigeria, Ethiopia is the second most populous country in Africa, with a total population of almost 126.5 million people in 2023 and an area of 1.1 million square kilometers. Children under the age of 15, individuals aged 15 to 64, and those aged 65 or above represent 44%, 52%, and 4%

of the total population, respectively [31,32]. The country is subdivided into nine regions and two city administrations.

The 2019 mini-EDHS sample was stratified and selected in two stages. Each region was stratified into urban and rural areas, yielding 21 sampling strata. Samples of enumeration areas (EAs) were selected independently in each stratum in the two stages. The first stage involved a selection of 305 EAs, with 93 in urban areas and 212 in rural areas. In the second stage, a fixed number of 30 households per cluster were selected with an equal probability of systematic selection from the newly created household listing [31]. We utilized the individual data (IR) set from the mini-EDHS 2019 for the analysis, and we obtained longitude and latitude coordinates at the cluster or EA level. The Ethiopian administrative boundaries shapefile was obtained from the Central Statistical Agency (CSA).

The outcome variable is teenage pregnancy, defined as women aged 15-19 years who had a live birth, who were pregnant with their first child at the time of the interview, or who had begun childbearing [33]. It is dichotomized as 0 = no (those who did not experience teenage pregnancy) and 1 = yes (those who experienced teenage pregnancy). The percentage of women who had begun childbearing was calculated by dividing the number of women who either had a birth or who were pregnant at the time of the interview by all women aged 15-19 years [33]. The independent variables included were age, marital status, religion, sex of the household head, education level, wealth index, media exposure, and contraceptive utilization. The community-level variables were residence and region; we also created aggregate community-level variables, which were generated by aggregating individual-level factors at the cluster level, which were community education, community poverty, community contraceptive utilization, community media exposure, and community percentage of marriage.

# Data processing and analysis

## Factors associated with teenage pregnancy

Due to the hierarchical nature of the EDHS data, teenagers within the same cluster exhibit similar characteristics compared to those in another cluster. Therefore, a multilevel binary logistic regression model that accounts for the heterogeneity between clusters was constructed. To assess the clustering effect, the intraclass correlation coefficient (ICC) was estimated. The ICC showed a significant clustering effect (ICC = 20.9%). The median odds ratio (MOR), which is the median value of the odds ratio between the highest risk area and lowest risk area when randomly picking out two areas, is the increased risk that would exist when moving to a different area with a higher level of risk [34]. The proportional change in variance (PCV) is the variation in the dependent variable explained by all individual- and community-level factors [35].

Four models were constructed for the multilevel logistic regression analysis. The first model was a null model without explanatory variables to determine the degree of cluster variation in teenage pregnancy. The second model was fitted with individual-level variables, the third model was fitted with community-level variables, and the fourth model was fitted with both individual- and community-level variables at the same time. Variables with p values $< 0.25$ in the bivariable analysis were added to the multivariable model. The associations between the dependent and independent variables were assessed and are presented as adjusted odds ratios (AORs) with 95% confidence intervals (CIs) at p values $< 0.05$.

## Spatial analysis

A spatial analysis was conducted by using ArcGIS version 10.7 and Python 3. To assess whether the spatial distribution of teenage pregnancy in Ethiopia is dispersed, clustered, or randomly

distributed, global spatial autocorrelation (global Moran's I) was utilized. A Moran's I value < 0.05 indicated that the distribution of teenage pregnancy in Ethiopia was nonrandom [36]. Hotspot analysis was conducted to identify statistically significant clustering areas using Getis-ordGi* statistics, which resulted in z scores and p-values indicating where high and low values of teenage pregnancy clustered spatially [37].

To comprehend the association between the density of a certain event and other various sociodemographic and environmental aspects of the population, spatial regression models are important [38]. After the identification of the high-risk areas (hotspot areas) of teenage pregnancy, factors that contributed to the spatial clustering of teenage pregnancy in these areas were determined at the EA level by a global and local spatial regression model: ordinary least squares (OLS) and GWR and MGWR, respectively. The spatial nonstationarity of the proportion of teenage pregnancy was ascertained by using global spatial autocorrelation before fitting the OLS, GWR, and MGWR models.

First, OLS was fitted to choose variables that are appropriate for the spatial variation of teenage pregnancy. Before proceeding to the GWR model, we first checked the six assumptions of the OLS model using exploratory regression. Exploratory regression is a data mining tool that helps us determine which combinations of explanatory variables meet all necessary OLS diagnostics [39]. We checked for the expected relationship of the explanatory variables, the statistical significance of each explanatory variable, the randomness of residuals (which should be normally distributed with no spatial patterns), the statistical nonsignificance of the Jarque-Bera statistics, freedom from multicollinearity in the explanatory variables (VIF < 7.5), and the model performance (strength of the adjusted R-squared) [40]. The Koenker Breusch–Pagan (BP) test was significant, indicating nonstationarity or heterogeneity in the relationship between the dependent and independent

variables. As a result, we utilized local spatial regression models (GWR and MGWR), which account for variations in the relationships between variables across different spatial locations, for this study [41].

A geographically weighted regression model was used to determine the predictor variables for teenage pregnancy among adolescents aged 15 to 19 years. The proportion of the outcome variable and all predictors was calculated for each cluster. Classical GWR assumes that all of the processes being modeled operate at the same spatial scale. However, the MGWR reduces this assumption by allowing different processes to operate at different spatial scales by deriving an optimal bandwidth vector in which each element indicates the spatial scale at which a particular process takes place. Hence, MGWR is preferable because covariate-specific bandwidths are obtained rather than a single average bandwidth and provides valuable information on the scale at which different processes operate. Model calibration and bandwidth vector selection in MGWR were conducted using a back-fitting algorithm. Model fitness was compared among the global and local models using the Akaike information criterion (AIC), residual sum of squares (RSS), and adjusted R-squared values [42,43].

## Ethical approval and consent to participate

The data were obtained from the Demographic and Health Surveys (DHS) Program and can be freely accessed from the program website (www.dhsprogram.com). As the study was a secondary data analysis of publicly available data from the MEASURE DHS program, ethical approval and participant consent were not necessary for this particular study. We requested the use of the DHS Program, and permission was granted to download and use the data for this study. Approval for the use of the data was obtained from the Measure DHS program, and the dataset was downloaded from https://dhsprogram.com/data/available-datasets.cfm. The IRB-approved procedures for DHS

public-use datasets do not allow the identification of respondents, households, or sample communities. Geographic identifiers only go down to regional levels, and each EA has a primary sampling unit (PSU) number without labels. Surveys collect GIS coordinates for the entire enumeration area, preventing specific enumeration areas from being identified.

# Results

## Descriptive results

A total of 2165 teenage girls aged 15 to 19 years were included in the study. The median age of the respondents was 17 years. One-fifth (20.4%) of the participants were married, and only 9.5% of the girls utilized contraceptives. One-third of the girls were urban residents, while a quarter of them (25.1%) were illiterate. Regarding regional states, the majority of the participants were from the Oromia region (851, 39.3%), and the minority of the participants were from the Harari region (7, 0.3%). (Table 1)

Table 1: Descriptive characteristics of the study participants with a prevalence of teenage pregnancy in Ethiopia, 2019 EDHS

| Variables | | Weighted frequency (percentage) | Teenage pregnancy | |
|---|---|---|---|---|
| | | | Yes (%) | No (%) |
| Total | | 2165 (100) | 281 (12.98) | 1884 (87.02) |
| Age | 15 | 506 (23.4) | 6 (1.07) | 500 (98.9) |
| | 16 | 451 (20.8) | 21 (4.67) | 430 (95.3) |
| | 17 | 328 (15.2) | 37 (11.4) | 291 (88.6) |
| | 18 | 608 (28.1) | 139 (23.0) | 468 (77.0) |
| | 19 | 272 (12.6) | 77 (28.5) | 195 (71.5) |
| Religion | Orthodox | 928 (42.9) | 86 (9.2) | 842 (90.8) |

| | | | | |
|---|---|---|---|---|
| | Muslim | 589 (27.2) | 79 (13.5) | 510 (86.5) |
| | Protestant | 614 (28.4) | 114 (18.7) | 499 (81.3) |
| | Others | 34 (1.6) | 2 (4.2) | 33 (95.8) |
| Literacy | Illiterate | 543 (25.1) | 161 (29.7) | 382 (70.3) |
| | Literate | 1622 (74.9) | 120 (7.4) | 1502 (92.6) |
| Education level | No education | 229 (10.6) | 75 (32.7) | 154 (67.3) |
| | Primary | 1437 (66.4) | 177 (12.3) | 1259 (87.7) |
| | Secondary | 432 (19.9) | 19 (4.3) | 413 (95.7) |
| | Higher | 67 (3.1) | 10 (14.9) | 57 (85.1) |
| Marital status | Single | 1725 (79.7) | 50 (2.9) | 1675 (97.1) |
| | Married | 441 (20.4) | 231 (52.5) | 209 (47.5) |
| Contraceptive utilization | Yes | 206 (9.5) | 93 (45.3) | 113 (54.7) |
| | No | 1959 (90.5) | 188 (9.6) | 1772 (90.4) |
| Wealth index | Poor | 720 (33.2) | 124 (17.2) | 596 (82.8) |
| | Middle | 442 (20.4) | 71 (16.1) | 371 (83.9) |
| | Rich | 1004 (46.4) | 87 (8.6) | 917 (91.4) |
| Media exposure | No | 1361 (62.9) | 193 (14.2) | 1168 (85.8) |
| | Yes | 804 (37.1) | 88 (11.0) | 716 (89.0) |
| Sex of household head | Male | 1658 (76.6) | 240 (14.5) | 1418 (85.5) |
| | Female | 507 (23.4) | 41 (8.1) | 466 (91.9) |
| **Community-level variables** | | | | |
| Residence | Urban | 720 (33.2) | 71 (9.9) | 648 (90.1) |
| | Rural | 1445 (66.8) | 210 (14.5) | 1236 (85.5) |
| Region | Tigray | 138 (6.4) | 16 (11.6) | 122 (88.4) |
| | Afar | 16 (0.75) | 5 (28.3) | 11 (71.7) |
| | Amhara | 498 (23.0) | 35 (7.0) | 464 (93.0) |
| | Oromia | 851 (39.3) | 129 (15.2) | 722 (84.8) |
| | Somali | 111 (5.1) | 20 (17.6) | 91 (82.4) |
| | Benishangul | 25 (1.2) | 5 (17.8) | 20 (82.2) |
| | SNNPR | 402 (18.6) | 63 (15.8) | 339 (84.2) |

| | | | | |
|---|---|---|---|---|
| | Gambella | 9 (0.42) | 2 (27.4) | 7 (72.6) |
| | Harari | 6 (0.29) | 1 (14.3) | 5 (85.7) |
| | Addis Ababa | 93 (4.3) | 4 (3.8) | 89 (96.2) |
| | Dire Dawa | 15 (0.7) | 2 (12.9) | 13 (87.1) |
| Community media exposure | Low proportion | 1116 (51.5) | 141 (12.7) | 974 (87.3) |
| | High proportion | 1050 (48.5) | 140 (13.3) | 910 (86.7) |
| Community education level | Low proportion | 869 (40.1) | 174 (20.0) | 695 (80.0) |
| | High proportion | 1296 (59.9) | 107 (8.3) | 1189 (91.7) |
| Community wealth index | Low proportion | 991 (45.8) | 155 (15.6) | 836 (84.4) |
| | High proportion | 1174 (54.2) | 126 (10.7) | 1048 (89.3) |
| Community contraceptive use | Low proportion | 1142 (52.7) | 119 (10.4) | 1023 (89.6) |
| | High proportion | 1023 (47.3) | 163 (15.9) | 861 (84.1) |
| Community literacy | Low proportion | 1014 (46.8) | 203 (20.0) | 811 (80.0) |
| | High proportion | 1151 (53.2) | 78 (6.8) | 1073 (93.2) |
| Community marital status | Low proportion | 1083 (50.0) | 55 (5.1) | 1028 (94.9) |
| | High proportion | 1082 (50.0) | 226 (20.9) | 856 (79.1) |

### Prevalence of teenage pregnancy in Ethiopia

The prevalence of teenage pregnancy among adolescents aged 15 to 19 years was 12.98% (95% CI: 11.63%, 14.46%). The highest percentage of teenage pregnancy was observed in the Afar region (28.3%), followed by the Gambella region (27.4%). The lowest prevalence was observed in Addis Ababa (3.8%) (Table 1).

## Spatial analysis

Global spatial autocorrelation analysis revealed that the spatial distribution of teenage pregnancy in Ethiopia significantly varied across the country, with a global Moran's index value of 0.04 and a p-value < 0.001. The Z score of 4.71 indicated that there was less than a 1% likelihood that the clustered pattern could be a result of random chance. (Fig 1)

**Fig 1: Global spatial autocorrelation analysis of teenage pregnancy among adolescents aged 15 to 19 years in Ethiopia, 2019**

Significant hotspot areas (areas with a high proportion of teenage pregnancy) of teenage pregnancy were observed in central and southern Afar; northern, central, and western Gambela; northeastern and southern central Oromia; and the eastern Somali region. Significant cod spot areas (areas with a low proportion of teenage pregnancy) were identified in central and southern Amhara, central Oromia, Addis Ababa, and northern SNNPR. (Fig 2)

**Fig 2: The Getis Ord Gi statistical analysis of hotspots of teenage pregnancy among adolescents aged 15 to 19 years in Ethiopia, 2019.** Shapefile source: (CSA, 2013; https://africaopendata.org/dataset/ethiopia-shapefiles); Map output: Own analysis using ArcGIS Software.

## Spatial regression analysis

To explore the assumptions of spatial regression and to estimate the coefficients of the selected variables on teenage pregnancy, OLS regression was conducted. The OLS model identified predictors of each hotspot of teenage pregnancy. The joint F-statistics and Wald statistics were significant, which shows that the model was statistically significant. The Koenker statistics was statistically significant, which is indicative of nonstationarity or heterogeneity of the relationship between the dependent and the independent variables across the study areas. Therefore, GWR was applied because it assumes that the relationship between the independent and dependent variables has spatial heterogeneity (as confirmed by Koenker statistics). No multicollinearity was observed between the selected explanatory variables. (Table 2)

Table 2: The ordinary least squares (OLS) analysis results for the 2019 EDHS

| Variable | Coefficient | Robust SE | Robust t-statistics | Robust probability | VIF |
|---|---|---|---|---|---|
| Intercept | 0.015 | 0.01 | 1.48 | 0.14 | --- |
| Proportion of poor wealth index | 0.016 | 0.03 | 0.47 | 0.64 | 3.30 |
| Proportion of married teenagers | 0.58 | 0.05 | 10.85 | 0.000000* | 1.24 |
| Proportion of Muslim teenagers | -0.026 | 0.017 | -1.54 | 0.13 | 1.21 |
| Proportion of illiterate teenagers | 0.098 | 0.047 | 2.09 | 0.036839* | 2.95 |
| Proportion of community poverty | 0.025 | 0.02 | 1.05 | 0.29 | 2.96 |
| Proportion of community illiteracy | -0.040 | 0.02 | -1.77 | 0.079 | 2.51 |
| **Ordinary least square regression diagnostics** | | | | | |
| Number of observations | 303 | Adjusted R-squared | 0.547 | | |
| Joint F-statistics | 61.7 | Prob(>F), (6,296) degree of freedom | 0.000000* | | |
| Joint Wald statistics | 258.7 | Prob (> chi-squared), (6) degree of freedom | 0.000000* | | |
| Koenker (BP) statistics | 49.9 | Prob (> chi-squared), (6) degree of freedom | 0.000000* | | |
| Jarque–Bera | 60.1 | Prob (> chi-squared), (2) degree of freedom | 0.000000* | | |

The local models, GWR and MGWR, were implemented to capture spatial heterogeneity. Both of the local models had better model fitness than the global model. The adjusted $R^2$ improved from 0.547 (OLS) to 0.634 (GWR) and 0.691 (MGWR). The adjusted $R^2$ indicates that the local models can explain more than % of the model variance. The residual sum of squares (RSS), which indicates unexplained variations, was very high in the OLS model (134.6) and was 93.7 (GWR) and 78.4 (MGWR). The local regression showed an AICc of 630.4, while the GWR reduced the AICc to 618.6, and the MGWR further decreased the AICc to 570.4. Relying on this information, it was confirmed that the local models improved the model performance. (Table 3)

Table 3: Comparison of the goodness-of-fit measures between the global and local models

| Model comparison parameter | Adj-$R^2$ | RSS | AICc |
|---|---|---|---|
| Model | | | |
| OLS | 0.547 | 134.573 | 630.444 |
| GWR | 0.634 | 93.709 | 618.603 |
| MGWR | 0.691 | 78.424 | 570.350 |

The GWR model has a single bandwidth of 103; thus, 103 nearest neighbors are considered to inform the construction of parameter estimates at each local regression point. Using the 103 bandwidths for each variable, 63.4% of the observations were correctly classified. In the case of MGWR, the optimum bandwidth for each variable is computed. By allowing multiple bandwidths in MGWR, the model theoretically accounts for an optimal number of neighbors for each parameter estimate, thus allowing better predictions for the response variables. The GWR model has a universal bandwidth of 103. In the case of MGWR, the bandwidths for each parameter are 111 for poor wealth index, 43 for being married, 301 for being Muslim, 301 for being illiterate, 292 for community poverty and 161 for community illiteracy. (Table 4)

Table 4: Summary of the GWR and MGWR model results for the predictors of teenage pregnancy in Ethiopia in the 2019 EDHS

| Variable | Mean | STD | Min | Median | Max | Bandwidth |
|---|---|---|---|---|---|---|
| **GWR model** | | | | | | |
| Intercept | -0.034 | 0.093 | -0.231 | -0.017 | 0.284 | 103 |
| Proportion of poor wealth index | 0.056 | 0.160 | -0.249 | 0.051 | 0.399 | 103 |
| Proportion of married teenagers | 0.670 | 0.137 | 0.302 | 0.700 | 1.012 | 103 |
| Proportion of Muslim teenagers | -0.071 | 0.074 | -0.259 | -0.062 | 0.120 | 103 |
| Proportion of illiterate teenagers | 0.135 | 0.106 | -0.305 | 0.115 | 0.388 | 103 |

| | | | | | | |
|---|---|---|---|---|---|---|
| Proportion of community poverty | 0.037 | 0.178 | -0.328 | 0.077 | 0.348 | 103 |
| Proportion of community illiteracy | -0.060 | 0.149 | -0.330 | -0.043 | 0.381 | 103 |
| **MGWR model** | | | | | | |
| Intercept | -0.052 | 0.197 | -0.373 | -0.079 | 0.662 | 50 |
| Proportion of poor wealth index | -0.008 | 0.101 | -0.222 | -0.050 | 0.292 | 111 |
| Proportion of married teenagers | 0.640 | 0.250 | -0.087 | 0.678 | 1.128 | 43 |
| Proportion of Muslim teenagers | -0.067 | 0.014 | -0.094 | -0.065 | -0.050 | 301 |
| Proportion of illiterate teenagers | 0.187 | 0.007 | 0.175 | 0.190 | 0.200 | 301 |
| Proportion of community poverty | 0.061 | 0.019 | 0.025 | 0.058 | 0.096 | 292 |
| Proportion of community illiteracy | -0.051 | 0.054 | -0.141 | -0.065 | 0.064 | 161 |

The figures below show the mapped GWR and MGWR coefficient estimates for the intercept and the covariates. In Fig 3, the local intercept in the MGWR is interpreted as the value of the outcome that would be expected if every location had exactly the same average value of each covariate. Based on the mapping of the intercept coefficients, the local intercept in the MGWR model can be interpreted as showing the level of teenage pregnancy that would be expected in each region if each of the regions had an average population profile or held all covariates constant. There is a high rate of teenage pregnancy in Gambella and some parts of western and northeastern Oromia. In contrast, there was a lower rate of teenage pregnancy in central and western Amhara. The intercept in the GWR was not significantly different from zero, but there was an outlier with a positive relationship in southern Afar at the border with Oromia.

**Fig 3: GWR (left) and MGWR (right) parameter estimates for the intercept showing local patterns of spatial heterogeneity. The gray dots are not significantly different from zero.** Shapefile source: (CSA, 2013; https://africaopendata.org/dataset/ethiopia-shapefiles); Map output: Own analysis using Python Software.

The parameter estimates for the proportion of being married were positively associated with teenage pregnancy in Tigray, Amhara, Afar, Somali, eastern SNNPR, Benishangul, Gambela, Harari, Dire Dawa and eastern Oromia. The GWR model showed a positive relationship with the outcome variable across the study area with no spatial heterogeneity. (Fig 4)

**Fig 4: GWR (left) and MGWR (right) parameter estimates for the proportion of married women showing local patterns of spatial heterogeneity. The gray dots are not significantly different from zero.** Shapefile source: (CSA, 2013; https://africaopendata.org/dataset/ethiopia-shapefiles); Map output: Own analysis using Python Software.

The proportion of being illiterate does not show any spatial heterogeneity, as it has a positive association with teenage pregnancy across the country. In the GWR model, it was not significantly different from zero because it has no statistically nonzero parameter estimate. (Fig 5)

**Fig 5: GWR (left) and MGWR (right) parameter estimates for the proportion of illiterate women showing local patterns of spatial heterogeneity. The gray dots are not significantly different from zero.** Shapefile source: (CSA, 2013; https://africaopendata.org/dataset/ethiopia-shapefiles); Map output: Own analysis using Python Software.

## Multilevel analysis of factors associated with teenage pregnancy

Multilevel mixed-effect logistic regression analysis was conducted to identify individual- and community-level variables that were significantly associated with teenage pregnancy. The ICC in the null model was 0.209, which shows that 20.9% of the variation in teenage pregnancy is explained by community-level variables (between-cluster variability). According to the MOR in the null model (2.43), there was a variation in teenage pregnancy between the clusters. When a teenage girl is switched from a low-risk cluster to a high-risk cluster, she is 2.43 times more likely

to have a teenage pregnancy. According to the final model (model 4), the PCV showed that 72% of the total variability in teenage pregnancy was explained by both individual- and community-level factors. Model 4 was the best-fitting model because it attained the highest log likelihood and the lowest deviance. (Table 5)

Table 5: Parameters and model fit statistics for multilevel models

| Parameters | Null model | Model 2 | Model 3 | Model 4 |
|---|---|---|---|---|
| Community level variance (SE) | 0.87 | 0.46 | 0.015 | 0.24 |
| ICC | 0.21 | 0.12 | 0.0046 | 0.069 |
| MOR | 2.43 | 1.91 | 1.125 | 1.589 |
| PCV | Ref | 0.47 | 0.98 | 0.72 |
| **Model fitness** | | | | |
| Deviance | 1715.1 | 946.82 | 1536.88 | 920.48 |
| Log-likelihood | -857.55 | -473.41 | -768.44 | -460.24 |

Based on the findings of Model 4, the factors that were found to have a significant association with teenage pregnancy were the age of the girls, literacy status, marital status, age of the household head and residence in the Gambela region.

The odds of having teenage pregnancy among girls aged 17, 18 and 19 years were 3.5 times greater (AOR = 3.54; 95% CI: 1.60, 7.81), 8.2 times greater (AOR = 8.21; 95% CI: 3.96, 17.0) and 15 times greater (AOR = 15.0; 95% CI: 6.84, 32.9), respectively, than teenagers aged 15 years. Literate teenagers had a 43% (AOR = 0.57; 95% CI: 0.35, 0.92) lower risk of teenage pregnancy than did those who were illiterate. Compared with single teenagers, married teenagers had 22.8 (AOR = 22.8; 95% CI: 14.1, 37.0) times greater odds of having a teenage pregnancy. With a one-year increase in the age of the household head, the odds of experiencing teenage pregnancy

decreased by 2% (AOR = 0.98; 95% CI: 0.98, 0.99). Teenagers residing in the Gambela region had 3.27 (AOR = 3.27; 95% CI: 1.21, 8.86) times greater odds of having a teenage pregnancy than did those residing in the Tigray region. (Table 6)

Table 6: Multilevel analysis of factors associated with teenage pregnancy among adolescents aged 15 to 19 years in Ethiopia, 2019 EDHS

| Explanatory variable | Null model | Model 2 AOR (95%CI) | Model 3 AOR (95%CI) | Model 4 AOR (95%CI) |
|---|---|---|---|---|
| **Age** | | | | |
| 15 | | 1 | | 1 |
| 16 | | 1.68 (0.70, 3.99) | | 1.77 (0.75, 4.18) |
| 17 | | 3.63 (1.64, 8.04) | | 3.54(1.60,7.81)** |
| 18 | | 8.38 (4.03, 17.4) | | 8.21(3.96,17.0)** |
| 19 | | 15.3 (7.02, 33.7) | | 15.0(6.84,32.9)** |
| **Religion** | | | | |
| Orthodox | | 1 | | 1 |
| Muslim | | 1.13 (0.69, 1.81) | | 0.89 (0.47, 1.68) |
| Protestant | | 2.14 (1.26, 3.64) | | 1.62 (0.85, 3.10) |
| Others | | 1.46 (0.35, 6.07) | | 1.08 (0.24, 4.83) |
| **Literacy** | | | | |
| Illiterate | | 1 | | 1 |
| Literate | | 0.65 (0.41, 1.02) | | 0.57 (0.35, 0.92)* |
| **Education level** | | | | |
| No education | | 1 | | 1 |
| Primary | | 0.77 (0.46, 1.29) | | 0.84 (0.48, 1.45) |
| Secondary | | 0.59 (0.28, 1.26) | | 0.71 (0.32, 1.54) |
| Higher | | 0.37 (0.11, 1.30) | | 0.39 (0.11, 1.46) |
| **Marital status** | | | | |
| Single | | 1 | | 1 |

| | | | | |
|---|---|---|---|---|
| Married | | 24.03(15.3,37.6) | | 22.8(14.1,37.0)** |
| **Contraceptive utilization** | | | | |
| Yes | | 1 | | 1 |
| No | | 0.97 (0.58, 1.63) | | 1.04 (0.59, 1.81) |
| **Wealth index** | | | | |
| Poor | | 1 | | 1 |
| Middle | | 0.97 (0.57, 1.67) | | 1.11 (0.63, 1.93) |
| Rich | | 0.49 (0.29, 0.80) | | 0.59 (0.32, 1.11) |
| **Media exposure** | | | | |
| No | | 1 | | 1 |
| Yes | | 1.25 (0.80, 1.93) | | 1.19 (0.74, 1.91) |
| **Age of HHH** | | 0.98 (0.97, 0.99) | | 0.98 (0.98, 0.99)* |
| **Residence** | | | | |
| Urban | | | 1 | 1 |
| Rural | | | 1.03 (0.68, 1.58) | 0.92 (0.50, 1.68) |
| **Region** | | | | |
| Tigray | | | 1 | 1 |
| Afar | | | 1.31 (0.68, 2.51) | 1.31 (0.44, 3.92) |
| Amhara | | | 0.50 (0.25, 1.01) | 0.45 (0.17, 1.18) |
| Oromia | | | 1.07 (0.59, 1.95) | 1.03 (0.39, 2.70) |
| Somali | | | 1.16 (0.59, 2.28) | 1.04 (0.34, 3.26) |
| Benishangul | | | 1.36 (0.72, 2.59) | 1.34 (0.48, 3.71) |
| SNNPR | | | 1.04 (0.55, 1.95) | 0.77 (0.28, 2.13) |
| Gambella | | | 2.04 (1.09, 3.81) | 3.27 (1.21, 8.86)* |
| Harari | | | 1.42 (0.70, 2.86) | 1.26 (0.39, 4.07) |
| Addis Ababa | | | 0.80 (0.29, 2.25) | 0.86 (0.23, 3.21) |
| Dire Dawa | | | 1.02 (0.52, 1.99) | 1.19 (0.39, 3.57) |
| **Community media exposure** | | | | |
| Low proportion | | | 1 | 1 |
| High proportion | | | 0.89 (0.65, 1.23) | 0.93 (0.50, 1.68) |

| | | | | |
|---|---|---|---|---|
| **Community education level** | | | | |
| Low proportion | | | 1 | 1 |
| High proportion | | | 0.81 (0.58, 1.12) | 0.81 (0.49, 1.32) |
| **Community wealth index** | | | | |
| Low proportion | | | 1 | 1 |
| High proportion | | | 0.77 (0.53, 1.12) | 0.83 (0.45, 1.54) |
| **Community contraceptive use** | | | | |
| Low proportion | | | 1 | 1 |
| High proportion | | | 1.25 (0.91, 1.71) | 1.00 (0.61, 1.63) |
| **Community literacy** | | | | |
| Low proportion | | | 1 | 1 |
| High proportion | | | 0.89 (0.63, 1.25) | 1.28 (0.76, 2.15) |
| **Community marital status** | | | | |
| Low proportion | | | 1 | 1 |
| High proportion | | | 4.84 (3.35, 7.00) | 1.34 (0.79, 2.25) |
| Intercept | 0.14 (0.11, 0.17) | 0.03(0.009, 0.08) | 0.07(0.03, 0.15) | 0.02(0.005, 0.12) |

*p value < 0.05; ** p value < 0.01; AOR, adjusted odds ratio; HHH, household head.

# Discussion

The spatial statistics showed that the spatial distribution of teenage pregnancy among adolescents aged 15 to 19 years had significant variation in Ethiopia. The hotspot areas of teenage pregnancy were identified in central and southern Afar, northern, central and western Gambela, northeastern and southern central Oromia and the eastern Somali region. A possible explanation might be that most of these areas are pastoralists and semipastoralists, characterized by seasonal mobility and lower utilization of contraceptives [44]. They also live in traditional settings and strongly adhere to traditional cultural values and beliefs, which leads to poor sexual and reproductive health outcomes [45].

According to the spatial regression analysis of the global model, the significant predictors of teenage pregnancy hotspots among adolescents aged 15 to 19 years were marital status and illiteracy. Local models were applied to the same predictors used in OLS to explore the local spatial variation in the relationship with the proportion of teenage pregnancy. As the MGWR model had the smallest AICc and RSS values and the highest adjusted $R^2$ value, it is a statistically preferable local model, which is in line with previous studies [46].

The MGWR analysis revealed a significant positive relationship between the proportion of being a married women and teenage pregnancy in central and southern Afar, most parts of Amhara, Tigray and Benishangul, eastern SNNPR, Gambela, Harari, Dire Dawa and eastern Oromia. This is because adolescents who are married are exposed to early sexual debut, which results in teenage pregnancy [23]. In most parts of Ethiopia, specifically rural areas, it is common for married girls to become pregnant soon after marriage to prove their fertility. Additionally, family planning utilizations are usually disapproved by the partner, in-laws and the community as a whole as a result of social norms, and little awareness and negative perception also play a role [47,48].

The proportion of being illiterate was significantly positively associated with teenage pregnancy throughout the country according to the MGWR model. This is because literacy increases the understanding of reproductive and sexual health through reading printed materials and contraceptive utilization [20]. Teenagers who are illiterate will have lower self-esteem and want to be accepted and validated by society through early childbearing [49].

According to the multivariable multilevel logistic regression, literacy, marital status, age of the girls, age of the household head and residence in the Gambela region were significantly associated with teenage pregnancy among adolescents aged 15 to 19 years. Literate teenagers have a lower risk of pregnancy during adolescence than do illiterate teenagers. This finding is in line with studies

conducted in Ethiopia [20], Zambia [50], Philadelphia, the USA [51] and Africa [52]. Giving an emphasis on female literacy will reduce teenage pregnancy. Teenagers who are married are more likely to experience teenage pregnancy than those who are single. This is supported by previously conducted similar studies in Ethiopia [20,23,25], Zambia [50], Uganda [53] and Nigeria [54]. As the frequency of sexual activity is greater among married teenagers, there is a greater likelihood of pregnancy in the absence of contraception than among those who are not married [55].

With respect to age, teenagers aged 17, 18 and 19 years had a greater risk of experiencing teenage pregnancy than did those aged 15 years. This finding is similar to those of studies conducted in Ethiopia [20] and Kenya [56]. This is because as age increases, the probability of engaging in sexual intercourse and marriage, which are proximal determinants of fertility, increases. Consequently, the chances of pregnancy and childbearing will also increase [57]. As the age of the household head increases, the chance of a teenage pregnancy decreases. This finding is consistent with a study from Rwanda [58]. This is because when household heads are older and more mature, they support teens under their household by giving them good parental support, including advising their teen girls on risky sex and fulfilling financial needs [59,60]. In terms of region, the odds of experiencing teenage pregnancy among adolescents aged 15 to 19 years who lived in the Gambela region were greater than those among adolescents in the Tigray region. This is attributable to the region’s high unmet need for family planning, greater use of polygamous practices, and early sexual initiation among adolescents, which exacerbates this condition [61].

The key strength of this study was the use of the most recent weighted nationally representative data with a relatively large sample size. To generalize findings at the national level by adjusting the data’s hierarchical nature, a multilevel model analysis was performed. The use of spatial and multiscale geographically weighted regression analysis assists in identifying factors that contribute

to spatial variation in teenage pregnancy. This research, however, has certain limitations that must be considered when interpreting the findings. Important variables such as age at first sex and marriage and demand for family planning were not included since the mini-EDHS of 2019. In addition, because of the cross-sectional nature of the EDHS data, the cause–effect relationship between the dependent and independent variables cannot be shown.

# Conclusion

There is significant spatial variation in teenage pregnancy among adolescents aged 15 to 19 years. The proportion of being illiterate and the proportion of being married were significantly positively associated with teenage pregnancy throughout the country according to the MGWR model. In the multivariable multilevel logistic regression, literacy, marital status, age of the girls, age of the household head and residence in the Gambela region were significantly associated with the outcome variable. To address this issue, stronger actions must be taken to implement the law, raising the minimum age for marriage to 18 years. Teenage marriage, which is the main cause of teenage pregnancy in Ethiopia, should be changed by addressing cultural norms that prioritize maintaining family status in the community.

Priority should also be given to enhancing levels of literacy. In addition to increasing girls' enrollment in formal schools, the government of Ethiopia must increase adult education programs for young women outside of school by incorporating reproductive health issues into the program. The population must also become aware of the consequences and adverse outcomes of teenage pregnancy. Generally, policies and implementations that focus on preventing early marriage, educating teenage girls, and creating awareness of reproductive health issues need to be strengthened.

## Abbreviations

AICc: corrected Akaike information criterion; AOR: adjusted odds ratio; CI: Confidence interval; CSA: central statistical agency; DHS: Demographic and Health Survey; EAs: Enumeration areas; EDHS: Ethiopian Demographic and Health Survey; GWR: Geographically weighted regression; HHH: Household Head; ICC: Intraclass correlation coefficient; LMICs: low- and middle-income countries; MOR: median odds ratio; MGWR: Multiscale Geographically Weighted Regression; PCV: Proportional change in variance; RSS: Residual sum of squares; OLS: Ordinary least squares; SSA: Sub-Saharan Africa; SNNPR: Southern Nations Nationalities and Peoples Region

## Consent for publication

Not applicable

## Availability of data and materials

The data are available can be accessed from: https://dhsprogram.com/data/dataset_admin/login_main.cfm?CFID=10818526&CFTOKEN=c131014a480fe56-4E0C6B7F-F551-E6B2-50. The data supporting the findings of this study can be obtained in anonymized form from the DHS website upon reasonable request.

## Competing interests

The authors declare that they have no competing interests.

## Funding

The authors received no funding.

## Authors' contributions

**Conceptualization**: Tsion Mulat Tebeje

**Investigations**: Tsion Mulat Tebeje, Mesfin Abebe, Ever Siyoum Shewarega, Gizaw Sisay, Beminate Lemma Seifu, Kusse Urmale Mare, Fantu Mamo Aragaw, Binyam Tariku Seboka

**Methodology**: Tsion Mulat Tebeje,

**Software**: Tsion Mulat Tebeje

**Writing of the original draft**: Tsion Mulat Tebeje

**Writing – review & editing**: Tsion Mulat Tebeje, Mesfin Abebe, Ever Siyoum Shewarega, Gizaw Sisay, Beminate Lemma Seifu, Kusse Urmale Mare, Fantu Mamo Aragaw, Binyam Tariku Seboka

## Acknowledgments

We would like to acknowledge the measure DHS program for providing the dataset to use the mini-EDHS 2019.

**References**

1. Kothari MT, Wang S, Head SK, Abderrahim N. Trends in adolescent reproductive and sexual behaviors [Internet]. Calverton, Maryland: ICF International; 2012. Available from: https://www.dhsprogram.com/pubs/pdf/CR29/CR29.pdf

2. Nove A, Matthews Z, Neal S, Camacho AV. Maternal mortality in adolescents compared with women of other ages: evidence from 144 countries. Lancet Glob Heal [Internet]. 2014 Mar 1;2(3):e155–64. Available from: https://doi.org/10.1016/S2214-109X(13)70179-7

3. Blanc AK, Winfrey W, Ross J. New Findings for Maternal Mortality Age Patterns: Aggregated Results for 38 Countries. PLoS One [Internet]. 2013 Apr 16;8(4):e59864.

Available from: https://doi.org/10.1371/journal.pone.0059864

4. World Health Organization. Trends in maternal mortality 2000 to 2020: estimates by WHO, UNICEF, UNFPA, World Bank Group and UNDESA/Population Division. Geneva; 2023.

5. Council NR, Population C on. Growing up global: The changing transitions to adulthood in developing countries. National Academies Press; 2005.

6. UNICEF. Progress for children: A report card on adolescents. New York UNICEF. 2012;(10).

7. Darroch JE, Woog V, Bankole A, Ashford LS. Adding it up: costs and benefits of meeting the contraceptive needs of adolescents. 2016.

8. Sully EA, Biddlecom A, Darroch JE, Riley T, Ashford LS, Lince-Deroche N, et al. Adding it up: investing in sexual and reproductive health 2019. 2020.

9. Shah IH, Åhman E. Unsafe abortion differentials in 2008 by age and developing country region: high burden among young women. Reprod Health Matters. 2012;20(39):169–73.

10. World Health Organization. SDG Target 3.7 Sexual and reproductive health [Internet]. Available from: https://www.who.int/data/gho/data/themes/topics/sdg-target-3_7-sexual-and-reproductive-health

11. Noori N, Proctor JL, Efevbera Y, Oron AP. The Effect of Adolescent Pregnancy on Child Mortality in 46 Low- and Middle-Income Countries. BMJ Glob Heal [Internet]. 2022 May 1;7(5):e007681. Available from: http://gh.bmj.com/content/7/5/e007681.abstract

12. Erulkar A, Ferede A, Ambelu W, Girma W, Amdemikael H, GebreMedhin B, et al.

Ethiopia young adult survey: A study in seven regions. 2010;

13. Central Statistical Authority/Ethiopia, ORC Macro. Ethiopia Demographic and Health Survey 2000 [Internet]. Addis Ababa, Ethiopia: Central Statistical Authority/Ethiopia and ORC Macro; 2001. Available from: http://dhsprogram.com/pubs/pdf/FR118/FR118.pdf

14. Central Statistical Agency/Ethiopia, ORC Marco. Ethiopia Demographic and Health Survey 2005 [Internet]. Addis Ababa, Ethiopia: Central Statistical Agency/Ethiopia and ORC Macro; 2006. Available from: http://dhsprogram.com/pubs/pdf/FR179/FR179.pdf

15. Central Statistical Agency/Ethiopia, ICF International. Ethiopia Demographic and Health Survey 2011 [Internet]. Addis Ababa, Ethiopia: Central Statistical Agency/Ethiopia and ICF International; 2012. Available from: http://dhsprogram.com/pubs/pdf/FR255/FR255.pdf

16. Central Statistical Agency - CSA/Ethiopia, ICF. Ethiopia Demographic and Health Survey 2016 [Internet]. Addis Ababa, Ethiopia: CSA and ICF; 2017. Available from: http://dhsprogram.com/pubs/pdf/FR328/FR328.pdf

17. Kaphagawani NC, Kalipeni E. Sociocultural factors contributing to teenage pregnancy in Zomba district, Malawi. Glob Public Health [Internet]. 2017 Jun 3;12(6):694–710. Available from: https://doi.org/10.1080/17441692.2016.1229354

18. World Health Organization. Adolescent pregnancy [Internet]. 2023. Available from: https://www.who.int/news-room/fact-sheets/detail/adolescent-pregnancy

19. Nuru Nabwire O. The association between the level of education and teenage pregnancy in Kenya A secondary analysis of Kenya Demographic and Health Survey 2014. 2023.

20. Bitew DA, Akalu Y, Belsti Y, Diress M, Gela YY, Belay DG, et al. Predictors of underage pregnancy among women aged 15–19 in highly prevalent regions of Ethiopia: a multilevel analysis based on EDHS, 2016. Sci Rep [Internet]. 2023;13(1):857. Available from: https://doi.org/10.1038/s41598-023-27805-y

21. Imamura M, Tucker J, Hannaford P, Da Silva MO, Astin M, Wyness L, et al. Factors associated with teenage pregnancy in the European Union countries: a systematic review. Eur J Public Health. 2007;17(6):630–6.

22. Ayele BG, Gebregzabher TG, Hailu TT, Assefa BA. Determinants of teenage pregnancy in Degua Tembien District, Tigray, Northern Ethiopia: A community-based case-control study. PLoS One [Internet]. 2018 Jul 25;13(7):e0200898. Available from: https://doi.org/10.1371/journal.pone.0200898

23. Kassa GM, Arowojolu AO, Odukogbe ATA, Yalew AW. Trends and determinants of teenage childbearing in Ethiopia: evidence from the 2000 to 2016 demographic and health surveys. Ital J Pediatr [Internet]. 2019;45(1):153. Available from: https://doi.org/10.1186/s13052-019-0745-4

24. Birhanu BE, Kebede DL, Kahsay AB, Belachew AB. Predictors of teenage pregnancy in Ethiopia: a multilevel analysis. BMC Public Health [Internet]. 2019;19(1):601. Available from: https://doi.org/10.1186/s12889-019-6845-7

25. Kefale B, Yalew M, Damtie Y, Adane B. A Multilevel Analysis of Factors Associated with Teenage Pregnancy in Ethiopia. Int J Womens Health. 2020;12:785–93.

26. Fikadu Y. Determinants of teenage pregnancy in Ethiopia: A Case–control study, 2019. Issues (St Louis Mo). 2019 Dec 13;17:112.

27. Tigabu S, Liyew AM, Geremew BM. Modeling spatial determinates of teenage pregnancy in Ethiopia; geographically weighted regression. BMC Womens Health [Internet]. 2021;21(1):254. Available from: https://doi.org/10.1186/s12905-021-01400-7

28. Chang Chien YM, Carver S, Comber A. Using geographically weighted models to explore how crowdsourced landscape perceptions relate to landscape physical characteristics. Landsc Urban Plan [Internet]. 2020;203:103904. Available from: https://www.sciencedirect.com/science/article/pii/S0169204620304667

29. Gao Y, Zhao J, Han L. Exploring the spatial heterogeneity of urban heat island effect and its relationship to block morphology with the geographically weighted regression model. Sustain Cities Soc [Internet]. 2022;76:103431. Available from: https://www.sciencedirect.com/science/article/pii/S2210670721007046

30. Fotheringham AS, Yu H, Wolf LJ, Oshan TM, Li Z. On the notion of 'bandwidth' in geographically weighted regression models of spatially varying processes. Int J Geogr Inf Sci [Internet]. 2022 Aug 3;36(8):1485–502. Available from: https://doi.org/10.1080/13658816.2022.2034829

31. Ethiopian Public Health Institute - EPHI, Federal Ministry of Health - FMoH, ICF. Ethiopia Mini Demographic and Health Survey 2019 [Internet]. Addis Ababa, Ethiopia: EPHI/FMoH/ICF; 2021. Available from: https://www.dhsprogram.com/pubs/pdf/FR363/FR363.pdf

32. World Population Review. Countries in Africa [Internet]. 2023. Available from: https://worldpopulationreview.com/country-rankings/countries-in-africa

33. Croft TN, Marshall AMJ, Allen CK, Arnold F, Assaf S, Balian S. Guide to DHS statistics.

Rockv ICF. 2018;645.

34. Merlo J, Chaix B, Ohlsson H, Beckman A, Johnell K, Hjerpe P, et al. A brief conceptual tutorial of multilevel analysis in social epidemiology: using measures of clustering in multilevel logistic regression to investigate contextual phenomena. J Epidemiol Community Health. 2006 Apr;60(4):290–7.

35. Merlo J, Yang M, Chaix B, Lynch J, Råstam L. A brief conceptual tutorial on multilevel analysis in social epidemiology: investigating contextual phenomena in different groups of people. J Epidemiol Community Health [Internet]. 2005 Sep 1;59(9):729 LP – 736. Available from: http://jech.bmj.com/content/59/9/729.abstract

36. Chen Y. New Approaches for Calculating Moran's Index of Spatial Autocorrelation. PLoS One [Internet]. 2013 Jul 12;8(7):e68336. Available from: https://doi.org/10.1371/journal.pone.0068336

37. Zulu LC, Kalipeni E, Johannes E. Analyzing spatial clustering and the spatiotemporal nature and trends of HIV/AIDS prevalence using GIS: the case of Malawi, 1994-2010. BMC Infect Dis [Internet]. 2014;14(1):285. Available from: https://doi.org/10.1186/1471-2334-14-285

38. Srinivasan S. Spatial Regression Models BT - Encyclopedia of GIS. In: Shekhar S, Xiong H, editors. Boston, MA: Springer US; 2008. p. 1102–5. Available from: https://doi.org/10.1007/978-0-387-35973-1_1294

39. Esri. How Exploratory Regression works [Internet]. Available from: https://pro.arcgis.com/en/pro-app/latest/tool-reference/spatial-statistics/how-exploratory-regression-works.htm

40. Tareke AA, Gashu KD, Endehabtu BF. Geographical weighted regression analysis of delayed antenatal care initiation and its factors among all reproductive-aged women in Ethiopia, 2016. Contracept Reprod Med [Internet]. 2022;7(1):23. Available from: https://doi.org/10.1186/s40834-022-00190-z

41. Charlton M, Fotheringham AS, Brunsdon C. Geographically weighted regression white paper. Kildare Natl Univ Irel Maynooth. 2009;1–14.

42. Fotheringham AS, Yang W, Kang W. Multiscale Geographically Weighted Regression (MGWR). Ann Am Assoc Geogr [Internet]. 2017 Nov 2;107(6):1247–65. Available from: https://doi.org/10.1080/24694452.2017.1352480

43. Li Z, Fotheringham AS, Oshan TM, Wolf LJ. Measuring Bandwidth Uncertainty in Multiscale Geographically Weighted Regression Using Akaike Weights. Ann Am Assoc Geogr [Internet]. 2020 Sep 2;110(5):1500–20. Available from: https://doi.org/10.1080/24694452.2019.1704680

44. Lakew Y, Reda AA, Tamene H, Benedict S, Deribe K. Geographical variation and factors influencing modern contraceptive use among married women in Ethiopia: evidence from a national population based survey. Reprod Health [Internet]. 2013;10(1):52. Available from: https://doi.org/10.1186/1742-4755-10-52

45. van der Kwaak A, Baltissen G, Plummer D, Ferris K, Nduba J. Understanding nomadic realities: case studies on sexual and reproductive health and rights in Eastern Africa. KIT Publishers; 2012.

46. Amegbor PM, Zhang Z, Dalgaard R, Sabel CE. Multilevel and spatial analyses of childhood malnutrition in Uganda: examining individual and contextual factors. Sci Rep

[Internet]. 2020;10(1):20019. Available from: https://doi.org/10.1038/s41598-020-76856-y

47. Ketema H, Erulkar A. Married Adolescents and Family Planning in Rural Ethiopia: Understanding Barriers and Opportunities. Afr J Reprod Health. 2018 Dec;22(4):26–34.

48. Chuta N. Neglected experiences: Fertility and childbearing among young people in Ethiopia. 2021;

49. Reading Partners. Early Reading Deficiencies Create Conditions that Enhance Teen Pregnancy Risk [Internet]. 2013. Available from: https://readingpartners.org/blog/early-reading-deficiencies-creating-conditions-that-enhance-teen-pregnancy/

50. Phiri M, Kasonde ME, Moyo N, Sikaluzwe M, Simona S. A multilevel analysis of trends and predictors associated with teenage pregnancy in Zambia (2001–2018). Reprod Health [Internet]. 2023;20(1):16. Available from: https://doi.org/10.1186/s12978-023-01567-2

51. Bennett IM, Frasso R, Bellamy SL, Wortham S, Gross KS. Pre-teen literacy and subsequent teenage childbearing in a US population. Contraception [Internet]. 2013 Apr 1;87(4):459–64. Available from: https://doi.org/10.1016/j.contraception.2012.08.020

52. Odejimi O, Bellingham-Young D. Teenage pregnancy in Africa: trends and determinants in the 21st century. J Heal Soc Care Improv 2016 Vol 1 issue 1 [Internet]. 2016;1(1):12–20. Available from: https://wlv.openrepository.com/bitstream/2436/620300/1/Teenage Pregnancy in Africa- Trend and Determinants in the 21st Century.pdf

53. Ochen AM, Chi PC, Lawoko S. Predictors of teenage pregnancy among girls aged 13–19 years in Uganda: a community based case-control study. BMC Pregnancy Childbirth

[Internet]. 2019;19(1):211. Available from: https://doi.org/10.1186/s12884-019-2347-y

54. Ayoade MA. Spatiotemporal Patterns of Teenage Pregnancy in Nigeria: Evidence from the 2008, 2013 and 2018 NDHS. Pap Appl Geogr [Internet]. 2021 Apr 3;7(2):161–82. Available from: https://doi.org/10.1080/23754931.2020.1852103

55. World Health Organization. Early marriages, adolescent and young pregnancies. Geneva WHO. 2012;1–4.

56. Were M. Determinants of teenage pregnancies: the case of Busia District in Kenya. Econ Hum Biol. 2007 Jul;5(2):322–39.

57. Alemayehu T, Haider J, Habte D. Determinants of adolescent fertility in Ethiopia. Ethiop J Heal Dev. 2010;24(1).

58. Uwizeye D, Muhayiteto R, Kantarama E, Wiehler S, Murangwa Y. Prevalence of teenage pregnancy and the associated contextual correlates in Rwanda. Heliyon. 2020 Oct;6(10):e05037.

59. Nwogwugwu NC. Socio-demographic determinants of adolescent fertility in Zambia. University of the Witwatersrand, Faculty of Humanities; 2013.

60. Domenico DM, Jones KH. Adolescent pregnancy in America: Causes and responses. J Vocat Spec Needs Educ. 2007;30(1):4–12.

61. Bol KN, Negera E, Gedefa AG. Pregnancy among adolescent girls in humanitarian settings: a case in refugee camp of Gambella regional state, community-based cross-sectional study, Southwest Ethiopia, 2021. BMJ Open [Internet]. 2022 Nov 1;12(11):e064732. Available from:

http://bmjopen.bmj.com/content/12/11/e064732.abstract

Figure

Click here to access/download;Figure;Fig1.tif.tif

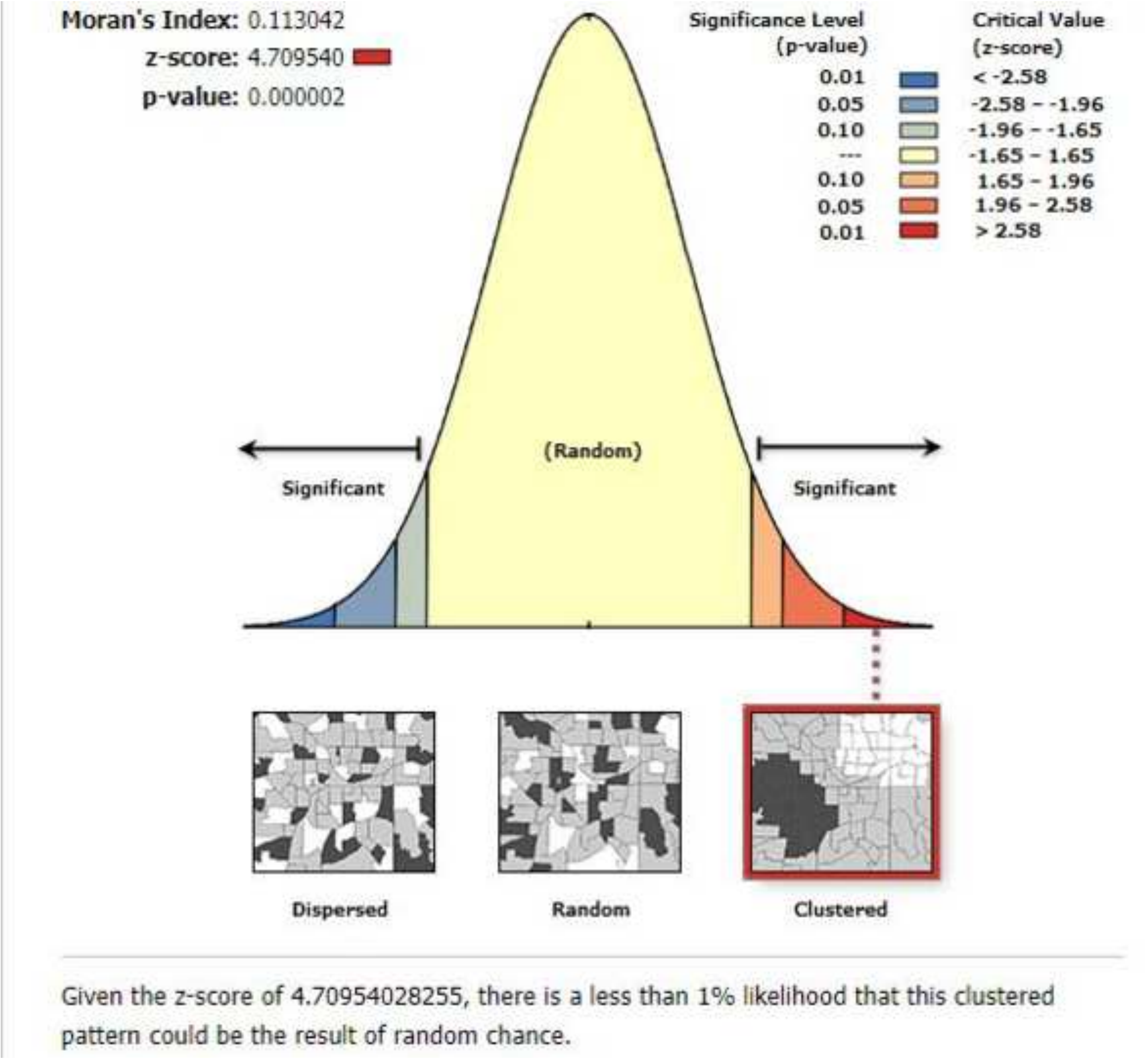

Figure

Click here to access/download;Figure;Fig2.tif.tif

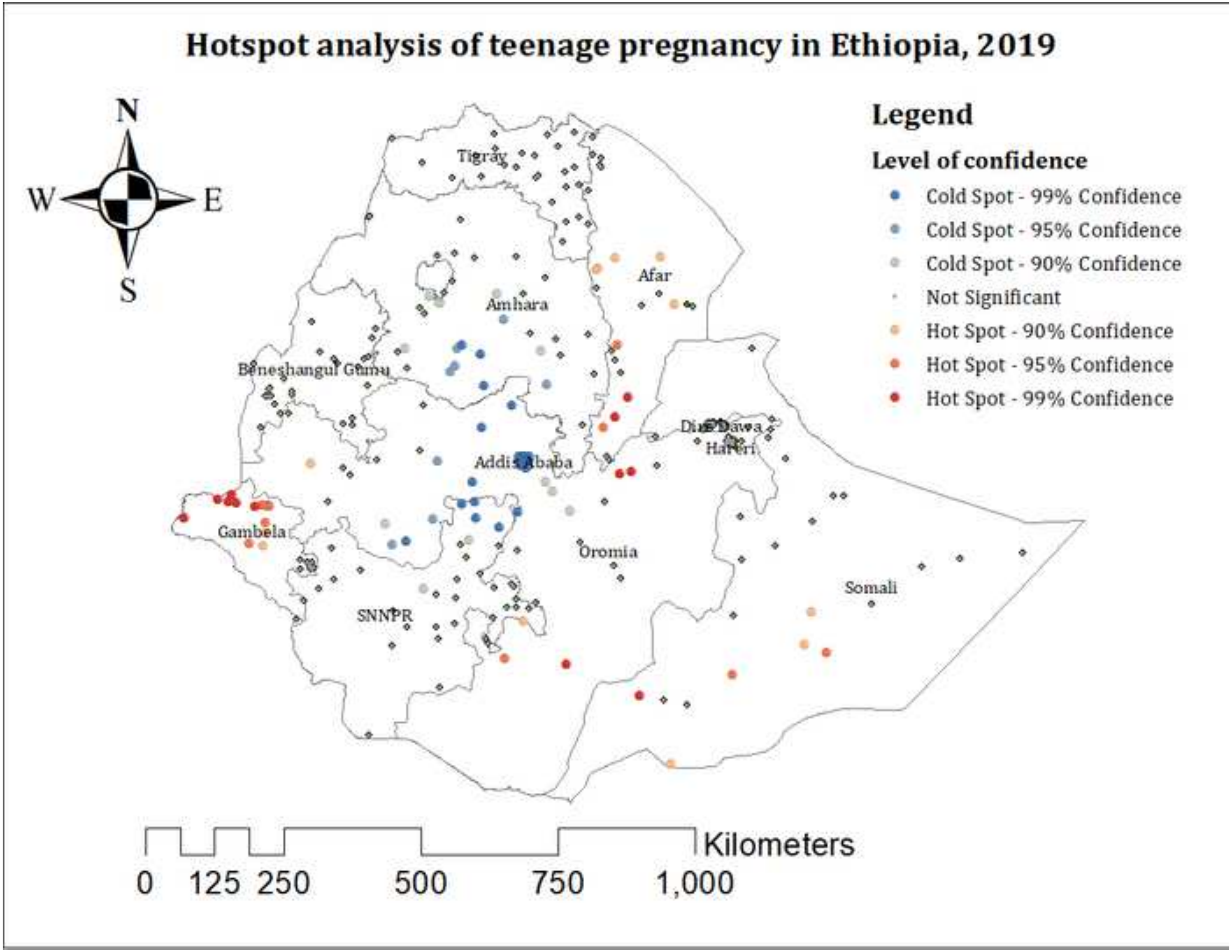

Figure

Click here to access/download;Figure;Fig3.tif.tif

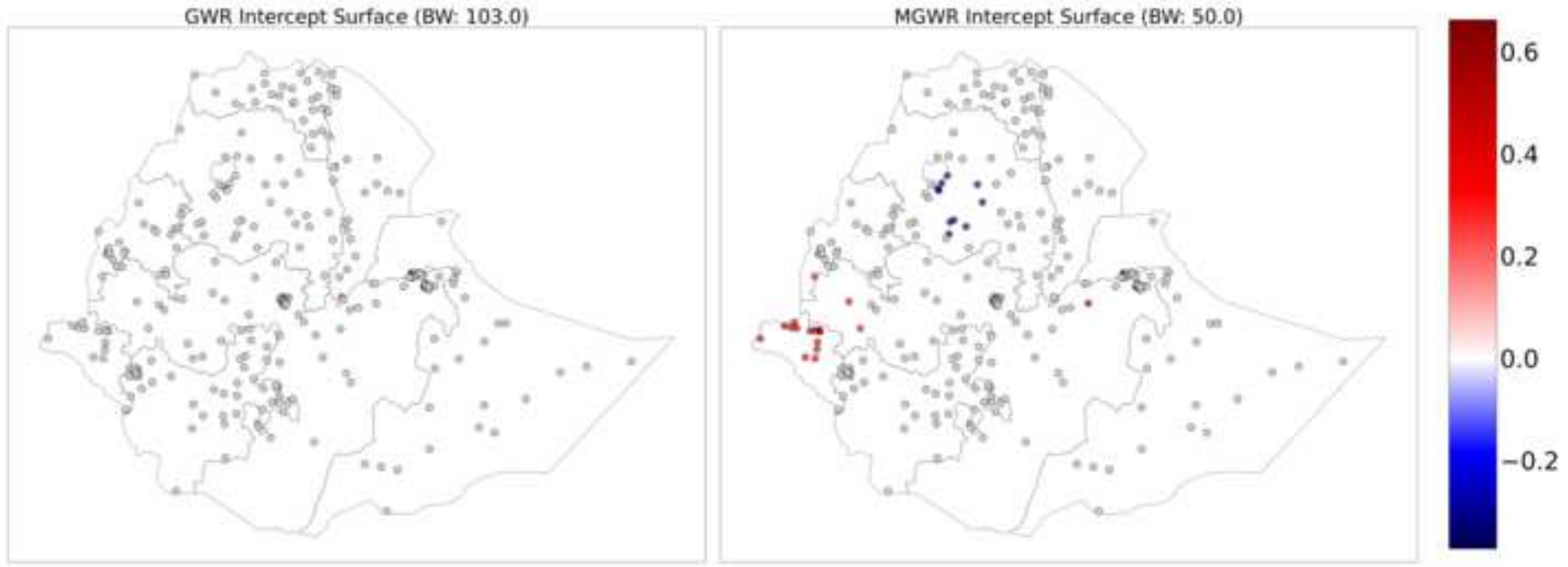

Figure

Click here to access/download;Figure;Fig4.tif.tif

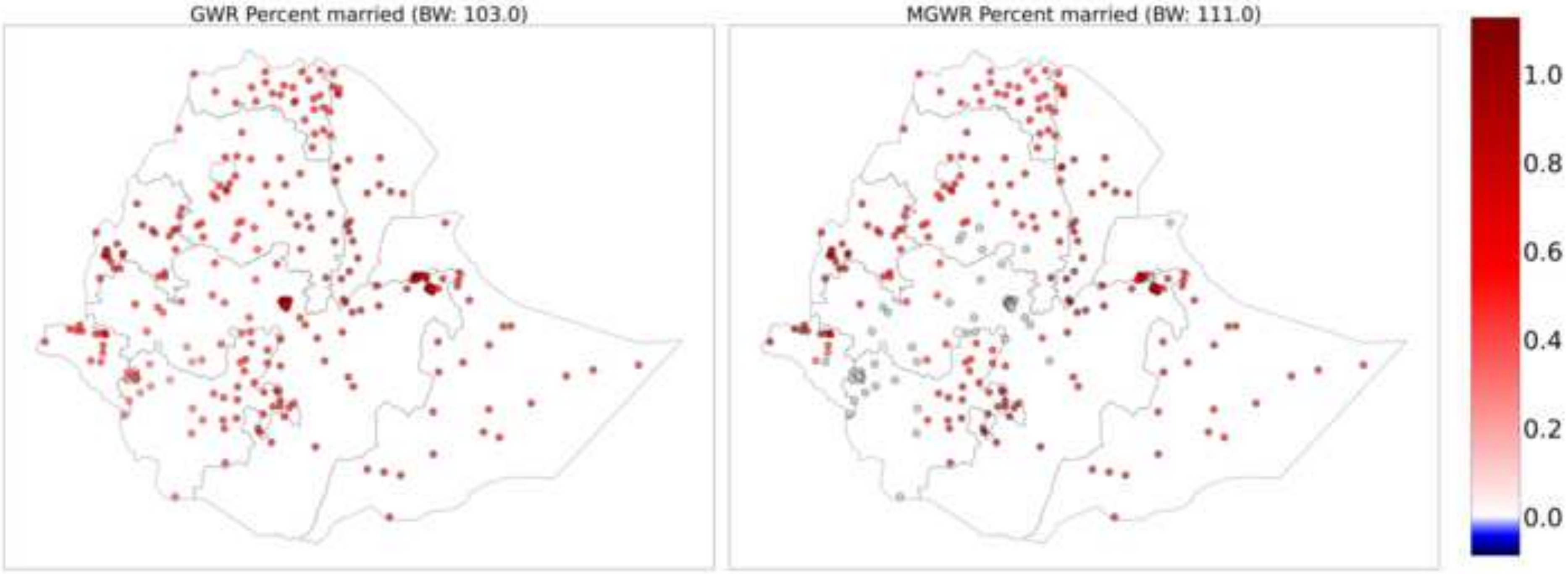

Figure

Click here to access/download;Figure;Fig5.tif.tif

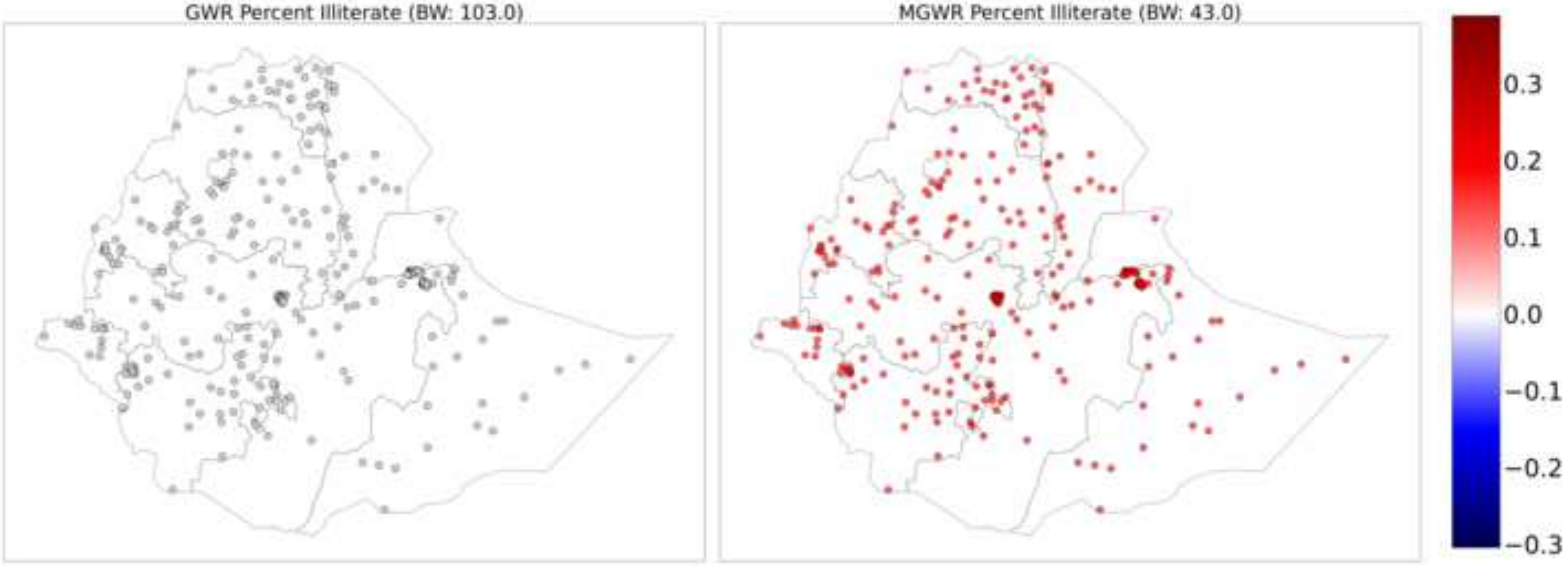

Click here to access/download
**Other**
AuthLetter_188507.pdf

# A multiscale geographically weighted regression analysis of teenage pregnancy and associated factors among adolescents aged 15 to 19 in Ethiopia using the 2019 mini-demographic and health survey

Tsion Mulat Tebeje[1*], Mesfin Abebe[2], Fantu Mamo Aragaw[3], Beminate Lemma Seifu[4], Kusse Urmale Mare[5], Ever Siyoum Shewarega[6], Gizaw Sisay[1], Binyam Tariku Seboka[1]

1. School of Public Health, College of Health Science and Medicine, Dilla University, Dilla, Ethiopia
2. Department of Midwifery, College of Health Science and Medicine, Dilla University, Dilla, Ethiopia
3. Department of Epidemiology and Biostatistics, Institute of Public Health, College of Medicine and Health Sciences, University of Gondar, Gondar, Ethiopia
4. Department of Public Health, College of Medicine and Health Sciences, Samara University, Afar, Ethiopia
5. Department of Nursing, College of Medicine and Health Sciences, Samara University, Afar, Ethiopia
6. Department of Reproductive Health, School of Public Health, College of Health Science and Medicine, Dilla University, Dilla, Ethiopia

*Corresponding author

Email: yemarina12@gmail.com (TMT)

# Abstract

**Background**: Teenage pregnancy remains one of the major reproductive health problems, especially in sub-Saharan African countries. It can lead to maternal and neonatal complications and social consequences. The proportion of teenage pregnancy differs across regions of Ethiopia. Thus, this study aimed to determine the spatial variation in determinants of teenage pregnancy among adolescents aged 15-19 years in Ethiopia using the 2019 Demographic and Health Survey (DHS).

**Methods**: This study included a total weighted sample of 2165 teenage girls aged 15 to 19 years. A mixed-effect binary logistic regression model was employed to consider the hierarchical nature of the DHS data using STATA version 17. Adjusted odds ratios with 95% confidence intervals are reported, and a p-value less than 0.05 was used to identify significant predictors. The spatial analysis was conducted with ArcGIS version 10.7 and Python 3. To identify factors associated with the hotspots of teenage pregnancy, a multiscale geographically weighted regression was performed. Spatial regression models were compared using adjusted R2, the corrected Akaike information criterion (AICc), and the residual sum of squares (RSS).

**Results**: The prevalence of teenage pregnancy among adolescents aged 15 to 19 years was 12.98% (95% CI: 11.6%, 14.5%). It was spatially clustered throughout the country with a significant Moran's I value. Significant hotspot areas were detected in central and southern Afar; northern, central, and western Gambela; northeastern and southern central Oromia; and the eastern Somali region. The MGWR analysis revealed that the significant predictors of spatial variations in teenage pregnancy were being illiterate and being married. According to the multivariable multilevel analysis, age 17 (AOR = 3.54; 95% CI: 1.60, 7.81), 18 (AOR = 8.21; 95% CI: 3.96, 17.0), 19

(AOR = 15.0; 95% CI: 6.84, 32.9), being literate (AOR = 0.57; 95% CI: 0.35, 0.92), being married (AOR = 22.8; 95% CI: 14.1, 37.0), age of household head (AOR = 0.98; 95% CI: 0.98, 0.99) and residence in the Gambela region (AOR = 3.27; 95% CI: 1.21, 8.86) were significantly associated with teenage pregnancy among adolescents aged 15 to 19.

**Conclusion**: Teenage pregnancy is a public health problem in Ethiopia. Policymakers should prioritize addressing early marriage and improving teenage literacy rates, with a focus on the Gambela region and other hotspot areas. It is crucial to implement policies aimed at transforming the traditional practice of early marriage and to take measures to enhance literacy levels and promote awareness about reproductive health. This will help ensure that young people have the opportunity to pursue education and make informed decisions about their reproductive health.

**Keywords**: Adolescents, Demographic and health survey, Ethiopia, Teenage pregnancy, MGWR

# Introduction

In developing countries, the highest number of teenage childbirths occur, as most girls marry early in life [1]. Approximately 20 countries in sub-Saharan Africa and Asia are responsible for 82% of adolescent maternal mortality [2]. ~~In comparison, girls~~ aged 15 to 19 years have a 28% greater risk of maternal mortality than women aged 20 to 24 years [3]. Pregnancy-related conditions are a significant cause of death among adolescent girls aged 15-19 years worldwide [4]. Girls aged 15 to 19 years make up 11% of all births worldwide [5]. Although there has been a decline in the rate of adolescent births, the total number of births has not decreased as much due to the growing adolescent population [6]. Each year, adolescents from low- and middle-income countries experience an estimated 21 million pregnancies, resulting in 12 million births [7]. Half of these pregnancies are unintended, and 55% of the unintended pregnancies often end in unsafe abortions

[8]. Annually, 3.2 million unsafe abortions take place among adolescents aged 15 to 19, accounting for 15% of the total global incidence [9].

Adolescents have a significant unmet need for sexual and reproductive health care. A total of 43% of girls aged 15 to 19 years have an unmet need for modern contraception, which is higher than that of women of reproductive age, who have a 24% unmet need [7]. Adolescent pregnancies lead to health consequences such as increased risks of eclampsia, puerperal endometritis, and systemic infections. There are also social consequences, including stigma, rejection, violence by partners or parents, and dropping out of school, which are commonly observed [10]. Additionally, adolescent pregnancy is related to lower child survival, which is attributable to biological and social risks [11].

Teenage pregnancy is a significant public health issue in Ethiopia, where there is limited knowledge and access to reproductive health services [12]. The country ranks third in terms of adolescent maternal deaths [2]. Data from the 2000, 2005, 2011, and 2016 Ethiopian Demographic and Health Survey (EDHS) show that teenage pregnancy rates in Ethiopia were 16%, 17%, 12%, and 13%, respectively [13–16]. When broken down by region, the highest percentage was in the Afar region (23%), and the lowest percentage was in the capital, Addis Ababa (3%) [16].

According to previous research, factors related to adolescent pregnancy in LMICs include lower education and socioeconomic status, child marriage, not utilizing contraception, child sexual abuse and early sexual activity, age, religious beliefs, limited knowledge of reproductive and sexual health, gender inequality, physical or sexual violence, lower family income, being in the 18-19 age group, maternal history of teenage pregnancy, and inadequate communication with parents about reproductive health issues [17–22].

Fertility and childbearing among young people are among the most neglected issues in Ethiopia. Some similar studies have been conducted in the country [23–27], but they were based on the 2016 EDHS. Four of the aforementioned studies [23–26] did not take into account the geographical variation in teenage pregnancy in Ethiopia. However, one of them [27] examined regional disparities and studied the factors that influence regional disparities in teenage pregnancy using geographically weighted regression (GWR). Our study used the most recent data from the mini-EDHS 2019 to provide updated information. Additionally, this study was conducted to address the limitation of the previous study, which used GWR to assess the relationship between teenage pregnancy and its determinants. GWR assumes that the relationship between the outcome and its predictors operates at the same spatial scale. This disregards the fact that different relationships may occur at various scales due to heterogeneity or nonstationarity in geospatial relationships. To address this methodological gap, we used Multiscale Geographically Weighted Regression (MGWR), which considers multiple scales and relaxes the assumption of GWR by determining parameter-specific bandwidths. This allows the relationships between independent and dependent variables to vary at different spatial scales [28–30]. The purpose of this study was to better understand the spatial context of the determinants of teenage pregnancy using an explicit multiscale approach. Therefore, our study aimed to explore the spatial determinants of teenage pregnancy among adolescents aged 15 to 19 years using the most recent EDHS.

# Materials and Methods

A community-based cross-sectional study was conducted in Ethiopia by using mini-EDHS 2019. After Nigeria, Ethiopia is the second most populous country in Africa, with a total population of almost 126.5 million people in 2023 and an area of 1.1 million square kilometers. Children under the age of 15, individuals aged 15 to 64, and those aged 65 or above represent 44%, 52%, and 4%

of the total population, respectively [31,32]. The country is subdivided into nine regions and two city administrations.

The 2019 mini-EDHS sample was stratified and selected in two stages. Each region was stratified into urban and rural areas, yielding 21 sampling strata. Samples of enumeration areas (EAs) were selected independently in each stratum in the two stages. The first stage involved a selection of 305 EAs, with 93 in urban areas and 212 in rural areas. In the second stage, a fixed number of 30 households per cluster were selected with an equal probability of systematic selection from the newly created household listing [31]. We utilized the individual data (IR) set from the mini-EDHS 2019 for the analysis, and we obtained longitude and latitude coordinates at the cluster or EA level. The Ethiopian administrative boundaries shapefile was obtained from the Central Statistical Agency (CSA).

The outcome variable is teenage pregnancy, defined as women aged 15-19 years who had a live birth, who were pregnant with their first child at the time of the interview, or who had begun childbearing [33]. It is dichotomized as 0 = no (those who did not experience teenage pregnancy) and 1 = yes (those who experienced teenage pregnancy). The percentage of women who had begun childbearing was calculated by dividing the number of women who either had a birth or who were pregnant at the time of the interview by all women aged 15-19 years [33]. The independent variables included were age, marital status, religion, sex of the household head, education level, wealth index, media exposure, and contraceptive utilization. The community-level variables were residence and region; we also created aggregate community-level variables, which were generated by aggregating individual-level factors at the cluster level, which were community education, community poverty, community contraceptive utilization, community media exposure, and community percentage of marriage.

## Data processing and analysis

### Factors associated with teenage pregnancy

Due to the hierarchical nature of the EDHS data, teenagers within the same cluster exhibit similar characteristics compared to those in another cluster. Therefore, a multilevel binary logistic regression model that accounts for the heterogeneity between clusters was constructed. To assess the clustering effect, the intraclass correlation coefficient (ICC) was estimated. The ICC showed a significant clustering effect (ICC = 20.9%). The median odds ratio (MOR), which is the median value of the odds ratio between the highest risk area and lowest risk area when randomly picking out two areas, is the increased risk that would exist when moving to a different area with a higher level of risk [34]. The proportional change in variance (PCV) is the variation in the dependent variable explained by all individual- and community-level factors [35].

Four models were constructed for the multilevel logistic regression analysis. The first model was a null model without explanatory variables to determine the degree of cluster variation in teenage pregnancy. The second model was fitted with individual-level variables, the third model was fitted with community-level variables, and the fourth model was fitted with both individual- and community-level variables at the same time. Variables with p values $< 0.25$ in the bivariable analysis were added to the multivariable model. The associations between the dependent and independent variables were assessed and are presented as adjusted odds ratios (AORs) with 95% confidence intervals (CIs) at p values $< 0.05$.

### Spatial analysis

A spatial analysis was conducted by using ArcGIS version 10.7 and Python 3. To assess whether the spatial distribution of teenage pregnancy in Ethiopia is dispersed, clustered, or randomly

distributed, global spatial autocorrelation (global Moran's I) was utilized. A Moran's I value < 0.05 indicated that the distribution of teenage pregnancy in Ethiopia was nonrandom [36]. Hotspot analysis was conducted to identify statistically significant clustering areas using Getis-ordGi* statistics, which resulted in z scores and p-values indicating where high and low values of teenage pregnancy clustered spatially [37].

To comprehend the association between the density of a certain event and other various sociodemographic and environmental aspects of the population, spatial regression models are important [38]. After the identification of the high-risk areas (hotspot areas) of teenage pregnancy, factors that contributed to the spatial clustering of teenage pregnancy in these areas were determined at the EA level by a global and local spatial regression model: ordinary least squares (OLS) and GWR and MGWR, respectively. The spatial nonstationarity of the proportion of teenage pregnancy was ascertained by using global spatial autocorrelation before fitting the OLS, GWR, and MGWR models.

First, OLS was fitted to choose variables that are appropriate for the spatial variation of teenage pregnancy. Before proceeding to the GWR model, we first checked the six assumptions of the OLS model using exploratory regression. Exploratory regression is a data mining tool that helps us determine which combinations of explanatory variables meet all necessary OLS diagnostics [39]. We checked for the expected relationship of the explanatory variables, the statistical significance of each explanatory variable, the randomness of residuals (which should be normally distributed with no spatial patterns), the statistical nonsignificance of the Jarque-Bera statistics, freedom from multicollinearity in the explanatory variables (VIF < 7.5), and the model performance (strength of the adjusted R-squared) [40]. The Koenker Breusch–Pagan (BP) test was significant, indicating nonstationarity or heterogeneity in the relationship between the dependent and independent

variables. As a result, we utilized local spatial regression models (GWR and MGWR), which account for variations in the relationships between variables across different spatial locations, for this study [41].

A geographically weighted regression model was used to determine the predictor variables for teenage pregnancy among adolescents aged 15 to 19 years. The proportion of the outcome variable and all predictors was calculated for each cluster. Classical GWR assumes that all of the processes being modeled operate at the same spatial scale. However, the MGWR reduces this assumption by allowing different processes to operate at different spatial scales by deriving an optimal bandwidth vector in which each element indicates the spatial scale at which a particular process takes place. Hence, MGWR is preferable because covariate-specific bandwidths are obtained rather than a single average bandwidth and provides valuable information on the scale at which different processes operate. Model calibration and bandwidth vector selection in MGWR were conducted using a back-fitting algorithm. Model fitness was compared among the global and local models using the Akaike information criterion (AIC), residual sum of squares (RSS), and adjusted R-squared values [42,43].

## Ethical approval and consent to participate

The data were obtained from the Demographic and Health Surveys (DHS) Program and can be freely accessed from the program website (www.dhsprogram.com). As the study was a secondary data analysis of publicly available data from the MEASURE DHS program, ethical approval and participant consent were not necessary for this particular study. We requested the use of the DHS Program, and permission was granted to download and use the data for this study. Approval for the use of the data was obtained from the Measure DHS program, and the dataset was downloaded from https://dhsprogram.com/data/available-datasets.cfm. The IRB-approved procedures for DHS

public-use datasets do not allow the identification of respondents, households, or sample communities. Geographic identifiers only go down to regional levels, and each EA has a primary sampling unit (PSU) number without labels. Surveys collect GIS coordinates for the entire enumeration area, preventing specific enumeration areas from being identified.

# Results

## Descriptive results

A total of 2165 teenage girls aged 15 to 19 years were included in the study. The median age of the respondents was 17 years. One-fifth (20.4%) of the participants were married, and only 9.5% of the girls utilized contraceptives. One-third of the girls were urban residents, while a quarter of them (25.1%) were illiterate. Regarding regional states, the majority of the participants were from the Oromia region (851, 39.3%), and the minority of the participants were from the Harari region (7, 0.3%). (Table 1)

Table 1: Descriptive characteristics of the study participants with a prevalence of teenage pregnancy in Ethiopia, 2019 EDHS

| Variables | | Weighted frequency (percentage) | Teenage pregnancy | |
|---|---|---|---|---|
| | | | Yes (%) | No (%) |
| Total | | 2165 (100) | 281 (12.98) | 1884 (87.02) |
| Age | 15 | 506 (23.4) | 6 (1.07) | 500 (98.9) |
| | 16 | 451 (20.8) | 21 (4.67) | 430 (95.3) |
| | 17 | 328 (15.2) | 37 (11.4) | 291 (88.6) |
| | 18 | 608 (28.1) | 139 (23.0) | 468 (77.0) |
| | 19 | 272 (12.6) | 77 (28.5) | 195 (71.5) |
| Religion | Orthodox | 928 (42.9) | 86 (9.2) | 842 (90.8) |

| | | | | |
|---|---|---|---|---|
| | Muslim | 589 (27.2) | 79 (13.5) | 510 (86.5) |
| | Protestant | 614 (28.4) | 114 (18.7) | 499 (81.3) |
| | Others | 34 (1.6) | 2 (4.2) | 33 (95.8) |
| Literacy | Illiterate | 543 (25.1) | 161 (29.7) | 382 (70.3) |
| | Literate | 1622 (74.9) | 120 (7.4) | 1502 (92.6) |
| Education level | No education | 229 (10.6) | 75 (32.7) | 154 (67.3) |
| | Primary | 1437 (66.4) | 177 (12.3) | 1259 (87.7) |
| | Secondary | 432 (19.9) | 19 (4.3) | 413 (95.7) |
| | Higher | 67 (3.1) | 10 (14.9) | 57 (85.1) |
| Marital status | Single | 1725 (79.7) | 50 (2.9) | 1675 (97.1) |
| | Married | 441 (20.4) | 231 (52.5) | 209 (47.5) |
| Contraceptive utilization | Yes | 206 (9.5) | 93 (45.3) | 113 (54.7) |
| | No | 1959 (90.5) | 188 (9.6) | 1772 (90.4) |
| Wealth index | Poor | 720 (33.2) | 124 (17.2) | 596 (82.8) |
| | Middle | 442 (20.4) | 71 (16.1) | 371 (83.9) |
| | Rich | 1004 (46.4) | 87 (8.6) | 917 (91.4) |
| Media exposure | No | 1361 (62.9) | 193 (14.2) | 1168 (85.8) |
| | Yes | 804 (37.1) | 88 (11.0) | 716 (89.0) |
| Sex of household head | Male | 1658 (76.6) | 240 (14.5) | 1418 (85.5) |
| | Female | 507 (23.4) | 41 (8.1) | 466 (91.9) |
| **Community-level variables** | | | | |
| Residence | Urban | 720 (33.2) | 71 (9.9) | 648 (90.1) |
| | Rural | 1445 (66.8) | 210 (14.5) | 1236 (85.5) |
| Region | Tigray | 138 (6.4) | 16 (11.6) | 122 (88.4) |
| | Afar | 16 (0.75) | 5 (28.3) | 11 (71.7) |
| | Amhara | 498 (23.0) | 35 (7.0) | 464 (93.0) |
| | Oromia | 851 (39.3) | 129 (15.2) | 722 (84.8) |
| | Somali | 111 (5.1) | 20 (17.6) | 91 (82.4) |
| | Benishangul | 25 (1.2) | 5 (17.8) | 20 (82.2) |
| | SNNPR | 402 (18.6) | 63 (15.8) | 339 (84.2) |

| | | | | |
|---|---|---|---|---|
| | Gambella | 9 (0.42) | 2 (27.4) | 7 (72.6) |
| | Harari | 6 (0.29) | 1 (14.3) | 5 (85.7) |
| | Addis Ababa | 93 (4.3) | 4 (3.8) | 89 (96.2) |
| | Dire Dawa | 15 (0.7) | 2 (12.9) | 13 (87.1) |
| Community media exposure | Low proportion | 1116 (51.5) | 141 (12.7) | 974 (87.3) |
| | High proportion | 1050 (48.5) | 140 (13.3) | 910 (86.7) |
| Community education level | Low proportion | 869 (40.1) | 174 (20.0) | 695 (80.0) |
| | High proportion | 1296 (59.9) | 107 (8.3) | 1189 (91.7) |
| Community wealth index | Low proportion | 991 (45.8) | 155 (15.6) | 836 (84.4) |
| | High proportion | 1174 (54.2) | 126 (10.7) | 1048 (89.3) |
| Community contraceptive use | Low proportion | 1142 (52.7) | 119 (10.4) | 1023 (89.6) |
| | High proportion | 1023 (47.3) | 163 (15.9) | 861 (84.1) |
| Community literacy | Low proportion | 1014 (46.8) | 203 (20.0) | 811 (80.0) |
| | High proportion | 1151 (53.2) | 78 (6.8) | 1073 (93.2) |
| Community marital status | Low proportion | 1083 (50.0) | 55 (5.1) | 1028 (94.9) |
| | High proportion | 1082 (50.0) | 226 (20.9) | 856 (79.1) |

### Prevalence of teenage pregnancy in Ethiopia

The prevalence of teenage pregnancy among adolescents aged 15 to 19 years was 12.98% (95% CI: 11.63%, 14.46%). The highest percentage of teenage pregnancy was observed in the Afar region (28.3%), followed by the Gambella region (27.4%). The lowest prevalence was observed in Addis Ababa (3.8%) (Table 1).

## Spatial analysis

Global spatial autocorrelation analysis revealed that the spatial distribution of teenage pregnancy in Ethiopia significantly varied across the country, with a global Moran’s index value of 0.04 and a p-value < 0.001. The Z score of 4.71 indicated that there was less than a 1% likelihood that the clustered pattern could be a result of random chance. (Fig 1)

**Fig 1: Global spatial autocorrelation analysis of teenage pregnancy among adolescents aged 15 to 19 years in Ethiopia, 2019**

Significant hotspot areas (areas with a high proportion of teenage pregnancy) of teenage pregnancy were observed in central and southern Afar; northern, central, and western Gambela; northeastern and southern central Oromia; and the eastern Somali region. Significant ~~cod~~ spot areas (areas with a low proportion of teenage pregnancy) were identified in central and southern Amhara, central Oromia, Addis Ababa, and northern SNNPR. (Fig 2)

**Fig 2: The Getis Ord Gi statistical analysis of hotspots of teenage pregnancy among adolescents aged 15 to 19 years in Ethiopia, 2019.** Shapefile source: (CSA, 2013; https://africaopendata.org/dataset/ethiopia-shapefiles); Map output: Own analysis using ArcGIS Software.

## Spatial regression analysis

To explore the assumptions of spatial regression and to estimate the coefficients of the selected variables on teenage pregnancy, OLS regression was conducted. The OLS model identified predictors of each hotspot of teenage pregnancy. The joint F-statistics and Wald statistics were significant, which shows that the model was statistically significant. The Koenker statistics was statistically significant, which is indicative of nonstationarity or heterogeneity of the relationship between the dependent and the independent variables across the study areas. Therefore, GWR was applied because it assumes that the relationship between the independent and dependent variables has spatial heterogeneity (as confirmed by Koenker statistics). No multicollinearity was observed between the selected explanatory variables. (Table 2)

Table 2: The ordinary least squares (OLS) analysis results for the 2019 EDHS

| Variable | Coefficient | Robust SE | Robust t-statistics | Robust probability | VIF |
|---|---|---|---|---|---|
| Intercept | 0.015 | 0.01 | 1.48 | 0.14 | --- |
| Proportion of poor wealth index | 0.016 | 0.03 | 0.47 | 0.64 | 3.30 |
| Proportion of married teenagers | 0.58 | 0.05 | 10.85 | 0.000000* | 1.24 |
| Proportion of Muslim teenagers | -0.026 | 0.017 | -1.54 | 0.13 | 1.21 |
| Proportion of illiterate teenagers | 0.098 | 0.047 | 2.09 | 0.036839* | 2.95 |
| Proportion of community poverty | 0.025 | 0.02 | 1.05 | 0.29 | 2.96 |
| Proportion of community illiteracy | -0.040 | 0.02 | -1.77 | 0.079 | 2.51 |
| **Ordinary least square regression diagnostics** | | | | | |
| Number of observations | 303 | Adjusted R-squared | | 0.547 | |
| Joint F-statistics | 61.7 | Prob(>F), (6,296) degree of freedom | | 0.000000* | |
| Joint Wald statistics | 258.7 | Prob (> chi-squared), (6) degree of freedom | | 0.000000* | |
| Koenker (BP) statistics | 49.9 | Prob (> chi-squared), (6) degree of freedom | | 0.000000* | |
| Jarque–Bera | 60.1 | Prob (> chi-squared), (2) degree of freedom | | 0.000000* | |

The local models, GWR and MGWR, were implemented to capture spatial heterogeneity. Both of the local models had better model fitness than the global model. The adjusted $R^2$ improved from 0.547 (OLS) to 0.634 (GWR) and 0.691 (MGWR). The adjusted $R^2$ indicates that the local models can explain more than % of the model variance. The residual sum of squares (RSS), which indicates unexplained variations, was very high in the OLS model (134.6) and was 93.7 (GWR) and 78.4 (MGWR). The local regression showed an AICc of 630.4, while the GWR reduced the AICc to 618.6, and the MGWR further decreased the AICc to 570.4. Relying on this information, it was confirmed that the local models improved the model performance. (Table 3)

Table 3: Comparison of the goodness-of-fit measures between the global and local models

| Model comparison parameter / Model | Adj-$R^2$ | RSS | AICc |
|---|---|---|---|
| OLS | 0.547 | 134.573 | 630.444 |
| GWR | 0.634 | 93.709 | 618.603 |
| MGWR | 0.691 | 78.424 | 570.350 |

The GWR model has a single bandwidth of 103; thus, 103 nearest neighbors are considered to inform the construction of parameter estimates at each local regression point. Using the 103 bandwidths for each variable, 63.4% of the observations were correctly classified. In the case of MGWR, the optimum bandwidth for each variable is computed. By allowing multiple bandwidths in MGWR, the model theoretically accounts for an optimal number of neighbors for each parameter estimate, thus allowing better predictions for the response variables. The GWR model has a universal bandwidth of 103. In the case of MGWR, the bandwidths for each parameter are 111 for poor wealth index, 43 for being married, 301 for being Muslim, 301 for being illiterate, 292 for community poverty and 161 for community illiteracy. (Table 4)

Table 4: Summary of the GWR and MGWR model results for the predictors of teenage pregnancy in Ethiopia in the 2019 EDHS

| Variable | Mean | STD | Min | Median | Max | Bandwidth |
|---|---|---|---|---|---|---|
| **GWR model** | | | | | | |
| Intercept | -0.034 | 0.093 | -0.231 | -0.017 | 0.284 | 103 |
| Proportion of poor wealth index | 0.056 | 0.160 | -0.249 | 0.051 | 0.399 | 103 |
| Proportion of married teenagers | 0.670 | 0.137 | 0.302 | 0.700 | 1.012 | 103 |
| Proportion of Muslim teenagers | -0.071 | 0.074 | -0.259 | -0.062 | 0.120 | 103 |
| Proportion of illiterate teenagers | 0.135 | 0.106 | -0.305 | 0.115 | 0.388 | 103 |

| | | | | | | |
|---|---|---|---|---|---|---|
| Proportion of community poverty | 0.037 | 0.178 | -0.328 | 0.077 | 0.348 | 103 |
| Proportion of community illiteracy | -0.060 | 0.149 | -0.330 | -0.043 | 0.381 | 103 |
| **MGWR model** | | | | | | |
| Intercept | -0.052 | 0.197 | -0.373 | -0.079 | 0.662 | 50 |
| Proportion of poor wealth index | -0.008 | 0.101 | -0.222 | -0.050 | 0.292 | 111 |
| Proportion of married teenagers | 0.640 | 0.250 | -0.087 | 0.678 | 1.128 | 43 |
| Proportion of Muslim teenagers | -0.067 | 0.014 | -0.094 | -0.065 | -0.050 | 301 |
| Proportion of illiterate teenagers | 0.187 | 0.007 | 0.175 | 0.190 | 0.200 | 301 |
| Proportion of community poverty | 0.061 | 0.019 | 0.025 | 0.058 | 0.096 | 292 |
| Proportion of community illiteracy | -0.051 | 0.054 | -0.141 | -0.065 | 0.064 | 161 |

The figures below show the mapped GWR and MGWR coefficient estimates for the intercept and the covariates. In Fig 3, the local intercept in the MGWR is interpreted as the value of the outcome that would be expected if every location had exactly the same average value of each covariate. Based on the mapping of the intercept coefficients, the local intercept in the MGWR model can be interpreted as showing the level of teenage pregnancy that would be expected in each region if each of the regions had an average population profile or held all covariates constant. There is a high rate of teenage pregnancy in Gambella and some parts of western and northeastern Oromia. In contrast, there was a lower rate of teenage pregnancy in central and western Amhara. The intercept in the GWR was not significantly different from zero, but there was an outlier with a positive relationship in southern Afar at the border with Oromia.

**Fig 3: GWR (left) and MGWR (right) parameter estimates for the intercept showing local patterns of spatial heterogeneity. The gray dots are not significantly different from zero.** Shapefile source: (CSA, 2013; https://africaopendata.org/dataset/ethiopia-shapefiles); Map output: Own analysis using Python Software.

The parameter estimates for the proportion of being married were positively associated with teenage pregnancy in Tigray, Amhara, Afar, Somali, eastern SNNPR, Benishangul, Gambela, Harari, Dire Dawa and eastern Oromia. The GWR model showed a positive relationship with the outcome variable across the study area with no spatial heterogeneity. (Fig 4)

**Fig 4: GWR (left) and MGWR (right) parameter estimates for the proportion of married women showing local patterns of spatial heterogeneity. The gray dots are not significantly different from zero.** Shapefile source: (CSA, 2013; https://africaopendata.org/dataset/ethiopia-shapefiles); Map output: Own analysis using Python Software.

The proportion of being illiterate does not show any spatial heterogeneity, as it has a positive association with teenage pregnancy across the country. In the GWR model, it was not significantly different from zero because it has no statistically nonzero parameter estimate. (Fig 5)

**Fig 5: GWR (left) and MGWR (right) parameter estimates for the proportion of illiterate women showing local patterns of spatial heterogeneity. The gray dots are not significantly different from zero.** Shapefile source: (CSA, 2013; https://africaopendata.org/dataset/ethiopia-shapefiles); Map output: Own analysis using Python Software.

## Multilevel analysis of factors associated with teenage pregnancy

Multilevel mixed-effect logistic regression analysis was conducted to identify individual- and community-level variables that were significantly associated with teenage pregnancy. The ICC in the null model was 0.209, which shows that 20.9% of the variation in teenage pregnancy is explained by community-level variables (between-cluster variability). According to the MOR in the null model (2.43), there was a variation in teenage pregnancy between the clusters. When a teenage girl is switched from a low-risk cluster to a high-risk cluster, she is 2.43 times more likely

to have a teenage pregnancy. According to the final model (model 4), the PCV showed that 72% of the total variability in teenage pregnancy was explained by both individual- and community-level factors. Model 4 was the best-fitting model because it attained the highest log likelihood and the lowest deviance. (Table 5)

Table 5: Parameters and model fit statistics for multilevel models

| Parameters | Null model | Model 2 | Model 3 | Model 4 |
|---|---|---|---|---|
| Community level variance (SE) | 0.87 | 0.46 | 0.015 | 0.24 |
| ICC | 0.21 | 0.12 | 0.0046 | 0.069 |
| MOR | 2.43 | 1.91 | 1.125 | 1.589 |
| PCV | Ref | 0.47 | 0.98 | 0.72 |
| **Model fitness** | | | | |
| Deviance | 1715.1 | 946.82 | 1536.88 | 920.48 |
| Log-likelihood | -857.55 | -473.41 | -768.44 | -460.24 |

Based on the findings of Model 4, the factors that were found to have a significant association with teenage pregnancy were the age of the girls, literacy status, marital status, age of the household head and residence in the Gambela region.

The odds of having teenage pregnancy among girls aged 17, 18 and 19 years were 3.5 times greater (AOR = 3.54; 95% CI: 1.60, 7.81), 8.2 times greater (AOR = 8.21; 95% CI: 3.96, 17.0) and 15 times greater (AOR = 15.0; 95% CI: 6.84, 32.9), respectively, than teenagers aged 15 years. Literate teenagers had a 43% (AOR = 0.57; 95% CI: 0.35, 0.92) lower risk of teenage pregnancy than did those who were illiterate. Compared with single teenagers, married teenagers had 22.8 (AOR = 22.8; 95% CI: 14.1, 37.0) times greater odds of having a teenage pregnancy. With a one-year increase in the age of the household head, the odds of experiencing teenage pregnancy

decreased by 2% (AOR = 0.98; 95% CI: 0.98, 0.99). Teenagers residing in the Gambela region had 3.27 (AOR = 3.27; 95% CI: 1.21, 8.86) times greater odds of having a teenage pregnancy than did those residing in the Tigray region. (Table 6)

Table 6: Multilevel analysis of factors associated with teenage pregnancy among adolescents aged 15 to 19 years in Ethiopia, 2019 EDHS

| Explanatory variable | Null model | Model 2 AOR (95%CI) | Model 3 AOR (95%CI) | Model 4 AOR (95%CI) |
|---|---|---|---|---|
| **Age** | | | | |
| 15 | | 1 | | 1 |
| 16 | | 1.68 (0.70, 3.99) | | 1.77 (0.75, 4.18) |
| 17 | | 3.63 (1.64, 8.04) | | 3.54(1.60,7.81)** |
| 18 | | 8.38 (4.03, 17.4) | | 8.21(3.96,17.0)** |
| 19 | | 15.3 (7.02, 33.7) | | 15.0(6.84,32.9)** |
| **Religion** | | | | |
| Orthodox | | 1 | | 1 |
| Muslim | | 1.13 (0.69, 1.81) | | 0.89 (0.47, 1.68) |
| Protestant | | 2.14 (1.26, 3.64) | | 1.62 (0.85, 3.10) |
| Others | | 1.46 (0.35, 6.07) | | 1.08 (0.24, 4.83) |
| **Literacy** | | | | |
| Illiterate | | 1 | | 1 |
| Literate | | 0.65 (0.41, 1.02) | | 0.57 (0.35, 0.92)* |
| **Education level** | | | | |
| No education | | 1 | | 1 |
| Primary | | 0.77 (0.46, 1.29) | | 0.84 (0.48, 1.45) |
| Secondary | | 0.59 (0.28, 1.26) | | 0.71 (0.32, 1.54) |
| Higher | | 0.37 (0.11, 1.30) | | 0.39 (0.11, 1.46) |
| **Marital status** | | | | |
| Single | | 1 | | 1 |

| | | | | |
|---|---|---|---|---|
| Married | | 24.03(15.3,37.6) | | 22.8(14.1,37.0)** |
| **Contraceptive utilization** | | | | |
| Yes | | 1 | | 1 |
| No | | 0.97 (0.58, 1.63) | | 1.04 (0.59, 1.81) |
| **Wealth index** | | | | |
| Poor | | 1 | | 1 |
| Middle | | 0.97 (0.57, 1.67) | | 1.11 (0.63, 1.93) |
| Rich | | 0.49 (0.29, 0.80) | | 0.59 (0.32, 1.11) |
| **Media exposure** | | | | |
| No | | 1 | | 1 |
| Yes | | 1.25 (0.80, 1.93) | | 1.19 (0.74, 1.91) |
| **Age of HHH** | | 0.98 (0.97, 0.99) | | 0.98 (0.98, 0.99)* |
| **Residence** | | | | |
| Urban | | | 1 | 1 |
| Rural | | | 1.03 (0.68, 1.58) | 0.92 (0.50, 1.68) |
| **Region** | | | | |
| Tigray | | | 1 | 1 |
| Afar | | | 1.31 (0.68, 2.51) | 1.31 (0.44, 3.92) |
| Amhara | | | 0.50 (0.25, 1.01) | 0.45 (0.17, 1.18) |
| Oromia | | | 1.07 (0.59, 1.95) | 1.03 (0.39, 2.70) |
| Somali | | | 1.16 (0.59, 2.28) | 1.04 (0.34, 3.26) |
| Benishangul | | | 1.36 (0.72, 2.59) | 1.34 (0.48, 3.71) |
| SNNPR | | | 1.04 (0.55, 1.95) | 0.77 (0.28, 2.13) |
| Gambella | | | 2.04 (1.09, 3.81) | 3.27 (1.21, 8.86)* |
| Harari | | | 1.42 (0.70, 2.86) | 1.26 (0.39, 4.07) |
| Addis Ababa | | | 0.80 (0.29, 2.25) | 0.86 (0.23, 3.21) |
| Dire Dawa | | | 1.02 (0.52, 1.99) | 1.19 (0.39, 3.57) |
| **Community media exposure** | | | | |
| Low proportion | | | 1 | 1 |
| High proportion | | | 0.89 (0.65, 1.23) | 0.93 (0.50, 1.68) |

| | | | | |
|---|---|---|---|---|
| **Community education level** | | | | |
| Low proportion | | | 1 | 1 |
| High proportion | | | 0.81 (0.58, 1.12) | 0.81 (0.49, 1.32) |
| **Community wealth index** | | | | |
| Low proportion | | | 1 | 1 |
| High proportion | | | 0.77 (0.53, 1.12) | 0.83 (0.45, 1.54) |
| **Community contraceptive use** | | | | |
| Low proportion | | | 1 | 1 |
| High proportion | | | 1.25 (0.91, 1.71) | 1.00 (0.61, 1.63) |
| **Community literacy** | | | | |
| Low proportion | | | 1 | 1 |
| High proportion | | | 0.89 (0.63, 1.25) | 1.28 (0.76, 2.15) |
| **Community marital status** | | | | |
| Low proportion | | | 1 | 1 |
| High proportion | | | 4.84 (3.35, 7.00) | 1.34 (0.79, 2.25) |
| Intercept | 0.14 (0.11, 0.17) | 0.03(0.009, 0.08) | 0.07(0.03, 0.15) | 0.02(0.005, 0.12) |

*p value < 0.05; ** p value < 0.01; AOR, adjusted odds ratio; HHH, household head.

# Discussion

The spatial statistics showed that the spatial distribution of teenage pregnancy among adolescents aged 15 to 19 years had significant variation in Ethiopia. The hotspot areas of teenage pregnancy were identified in central and southern Afar, northern, central and western Gambela, northeastern and southern central Oromia and the eastern Somali region. A possible explanation might be that most of these areas are pastoralists and semipastoralists, characterized by seasonal mobility and lower utilization of contraceptives [44]. They also live in traditional settings and strongly adhere to traditional cultural values and beliefs, which leads to poor sexual and reproductive health outcomes [45].

According to the spatial regression analysis of the global model, the significant predictors of teenage pregnancy hotspots among adolescents aged 15 to 19 years were marital status and illiteracy. Local models were applied to the same predictors used in OLS to explore the local spatial variation in the relationship with the proportion of teenage pregnancy. As the MGWR model had the smallest AICc and RSS values and the highest adjusted $R^2$ value, it is a statistically preferable local model, which is in line with previous studies [46].

The MGWR analysis revealed a significant positive relationship between the proportion of being a married women and teenage pregnancy in central and southern Afar, most parts of Amhara, Tigray and Benishangul, eastern SNNPR, Gambela, Harari, Dire Dawa and eastern Oromia. This is because adolescents who are married are exposed to early sexual debut, which results in teenage pregnancy [23]. In most parts of Ethiopia, specifically rural areas, it is common for married girls to become pregnant soon after marriage to prove their fertility. Additionally, family planning utilizations are usually disapproved by the partner, in-laws and the community as a whole as a result of social norms, and little awareness and negative perception also play a role [47,48].

The proportion of being illiterate was significantly positively associated with teenage pregnancy throughout the country according to the MGWR model. This is because literacy increases the understanding of reproductive and sexual health through reading printed materials and contraceptive utilization [20]. Teenagers who are illiterate will have lower self-esteem and want to be accepted and validated by society through early childbearing [49].

According to the multivariable multilevel logistic regression, literacy, marital status, age of the girls, age of the household head and residence in the Gambela region were significantly associated with teenage pregnancy among adolescents aged 15 to 19 years. Literate teenagers have a lower risk of pregnancy during adolescence than do illiterate teenagers. This finding is in line with studies

conducted in Ethiopia [20], Zambia [50], Philadelphia, the USA [51] and Africa [52]. Giving an emphasis on female literacy will reduce teenage pregnancy. Teenagers who are married are more likely to experience teenage pregnancy than those who are single. This is supported by previously conducted similar studies in Ethiopia [20,23,25], Zambia [50], Uganda [53] and Nigeria [54]. As the frequency of sexual activity is greater among married teenagers, there is a greater likelihood of pregnancy in the absence of contraception than among those who are not married [55].

With respect to age, teenagers aged 17, 18 and 19 years had a greater risk of experiencing teenage pregnancy than did those aged 15 years. This finding is similar to those of studies conducted in Ethiopia [20] and Kenya [56]. This is because as age increases, the probability of engaging in sexual intercourse and marriage, which are proximal determinants of fertility, i[illegible]eases. Consequently, the chances of pregnancy and childbearing will also increase [57]. As the age of the household head increases, the chance of a teenage pregnancy decreases. This finding is consistent with a study from Rwanda [58]. This is because when household heads are older and more mature, they support teens under their household by giving them good parental support, including advising their teen girls on risky sex and fulfilling financial needs [59,60]. In terms of region, the odds of experiencing teenage pregnancy among adolescents aged 15 to 19 years who lived in the Gambela region were greater than those among adolescents in the Tigray region. This is attributable to the region's high unmet need for family planning, greater use of polygamous practices, and early sexual initiation among adolescents, which exacerbates this condition [61].

The key strength of this study was the use of the most recent weighted nationally representative data with a relatively large sample size. To generalize findings at the national level by adjusting the data's hierarchical nature, a multilevel model analysis was performed. The use of spatial and multiscale geographically weighted regression analysis assists in identifying factors that contribute

to spatial variation in teenage pregnancy. This research, however, has certain limitations that must be considered when interpreting the findings. Important variables such as age at first sex and marriage and demand for family planning were not included since the mini-EDHS of 2019. In addition, because of the cross-sectional nature of the EDHS data, the cause–effect relationship between the dependent and independent variables cannot be shown.

## Conclusion

There is significant spatial variation in teenage pregnancy among adolescents aged 15 to 19 years. The proportion of being illiterate and the proportion of being married were significantly positively associated with teenage pregnancy throughout the country according to the MGWR model. In the multivariable multilevel logistic regression, literacy, marital status, age of the girls, age of the household head and residence in the Gambela region were significantly associated with the outcome variable. To address this issue, stronger actions must be taken to implement the law, raising the minimum age for marriage to 18 years. Teenage marriage, which is the main cause of teenage pregnancy in Ethiopia, should be changed by addressing cultural norms that prioritize maintaining family status in the community.

Priority should also be given to enhancing levels of literacy. In addition to increasing girls' enrollment in formal schools, the government of Ethiopia must increase adult education programs for young women outside of school by incorporating reproductive health issues into the program. The population must also become aware of the consequences and adverse outcomes of teenage pregnancy. Generally, policies and implementations that focus on preventing early marriage, educating teenage girls, and creating awareness of reproductive health issues need to be strengthened.

## Abbreviations

AICc: corrected Akaike information criterion; AOR: adjusted odds ratio; CI: Confidence interval; CSA: central statistical agency; DHS: Demographic and Health Survey; EAs: Enumeration areas; EDHS: Ethiopian Demographic and Health Survey; GWR: Geographically weighted regression; HHH: Household Head; ICC: Intraclass correlation coefficient; LMICs: low- and middle-income countries; MOR: median odds ratio; MGWR: Multiscale Geographically Weighted Regression; PCV: Proportional change in variance; RSS: Residual sum of squares; OLS: Ordinary least squares; SSA: Sub-Saharan Africa; SNNPR: Southern Nations Nationalities and Peoples Region

## Consent for publication

Not applicable

## Availability of data and materials

The data are available can be accessed from: https://dhsprogram.com/data/dataset_admin/login_main.cfm?CFID=10818526&CFTOKEN=c131014a480fe56-4E0C6B7F-F551-E6B2-50. The data supporting the findings of this study can be obtained in anonymized form from the DHS website upon reasonable request.

## Competing interests

The authors declare that they have no competing interests.

## Funding

The authors received no funding.

## Authors' contributions

**Conceptualization**: Tsion Mulat Tebeje

**Investigations**: Tsion Mulat Tebeje, Mesfin Abebe, Ever Siyoum Shewarega, Gizaw Sisay, Beminate Lemma Seifu, Kusse Urmale Mare, Fantu Mamo Aragaw, Binyam Tariku Seboka

**Methodology**: Tsion Mulat Tebeje,

**Software**: Tsion Mulat Tebeje

**Writing of the original draft**: Tsion Mulat Tebeje

**Writing – review & editing**: Tsion Mulat Tebeje, Mesfin Abebe, Ever Siyoum Shewarega, Gizaw Sisay, Beminate Lemma Seifu, Kusse Urmale Mare, Fantu Mamo Aragaw, Binyam Tariku Seboka

## Acknowledgments

We would like to acknowledge the measure DHS program for providing the dataset to use the mini-EDHS 2019.

**References**

1. Kothari MT, Wang S, Head SK, Abderrahim N. Trends in adolescent reproductive and sexual behaviors [Internet]. Calverton, Maryland: ICF International; 2012. Available from: https://www.dhsprogram.com/pubs/pdf/CR29/CR29.pdf

2. Nove A, Matthews Z, Neal S, Camacho AV. Maternal mortality in adolescents compared with women of other ages: evidence from 144 countries. Lancet Glob Heal [Internet]. 2014 Mar 1;2(3):e155–64. Available from: https://doi.org/10.1016/S2214-109X(13)70179-7

3. Blanc AK, Winfrey W, Ross J. New Findings for Maternal Mortality Age Patterns: Aggregated Results for 38 Countries. PLoS One [Internet]. 2013 Apr 16;8(4):e59864. Available from: https://doi.org/10.1371/journal.pone.0059864

4. World Health Organization. Trends in maternal mortality 2000 to 2020: estimates by WHO, UNICEF, UNFPA, World Bank Group and UNDESA/Population Division. Geneva; 2023.

5. Council NR, Population C on. Growing up global: The changing transitions to adulthood in developing countries. National Academies Press; 2005.

6. UNICEF. Progress for children: A report card on adolescents. New York UNICEF. 2012;(10).

7. Darroch JE, Woog V, Bankole A, Ashford LS. Adding it up: costs and benefits of meeting the contraceptive needs of adolescents. 2016.

8. Sully EA, Biddlecom A, Darroch JE, Riley T, Ashford LS, Lince-Deroche N, et al. Adding it up: investing in sexual and reproductive health 2019. 2020.

9. Shah IH, Åhman E. Unsafe abortion differentials in 2008 by age and developing country region: high burden among young women. Reprod Health Matters. 2012;20(39):169–73.

10. World Health Organization. SDG Target 3.7 Sexual and reproductive health [Internet]. Available from: https://www.who.int/data/gho/data/themes/topics/sdg-target-3_7-sexual-and-reproductive-health

11. Noori N, Proctor JL, Efevbera Y, Oron AP. The Effect of Adolescent Pregnancy on Child Mortality in 46 Low- and Middle-Income Countries. BMJ Glob Heal [Internet]. 2022 May

1;7(5):e007681. Available from: http://gh.bmj.com/content/7/5/e007681.abstract

12. Erulkar A, Ferede A, Ambelu W, Girma W, Amdemikael H, GebreMedhin B, et al. Ethiopia young adult survey: A study in seven regions. 2010;

13. Central Statistical Authority/Ethiopia, ORC Macro. Ethiopia Demographic and Health Survey 2000 [Internet]. Addis Ababa, Ethiopia: Central Statistical Authority/Ethiopia and ORC Macro; 2001. Available from: http://dhsprogram.com/pubs/pdf/FR118/FR118.pdf

14. Central Statistical Agency/Ethiopia, ORC Marco. Ethiopia Demographic and Health Survey 2005 [Internet]. Addis Ababa, Ethiopia: Central Statistical Agency/Ethiopia and ORC Macro; 2006. Available from: http://dhsprogram.com/pubs/pdf/FR179/FR179.pdf

15. Central Statistical Agency/Ethiopia, ICF International. Ethiopia Demographic and Health Survey 2011 [Internet]. Addis Ababa, Ethiopia: Central Statistical Agency/Ethiopia and ICF International; 2012. Available from: http://dhsprogram.com/pubs/pdf/FR255/FR255.pdf

16. Central Statistical Agency - CSA/Ethiopia, ICF. Ethiopia Demographic and Health Survey 2016 [Internet]. Addis Ababa, Ethiopia: CSA and ICF; 2017. Available from: http://dhsprogram.com/pubs/pdf/FR328/FR328.pdf

17. Kaphagawani NC, Kalipeni E. Sociocultural factors contributing to teenage pregnancy in Zomba district, Malawi. Glob Public Health [Internet]. 2017 Jun 3;12(6):694–710. Available from: https://doi.org/10.1080/17441692.2016.1229354

18. World Health Organization. Adolescent pregnancy [Internet]. 2023. Available from: https://www.who.int/news-room/fact-sheets/detail/adolescent-pregnancy

19. Nuru Nabwire O. The association between the level of education and teenage pregnancy in Kenya A secondary analysis of Kenya Demographic and Health Survey 2014. 2023.

20. Bitew DA, Akalu Y, Belsti Y, Diress M, Gela YY, Belay DG, et al. Predictors of underage pregnancy among women aged 15–19 in highly prevalent regions of Ethiopia: a multilevel analysis based on EDHS, 2016. Sci Rep [Internet]. 2023;13(1):857. Available from: https://doi.org/10.1038/s41598-023-27805-y

21. Imamura M, Tucker J, Hannaford P, Da Silva MO, Astin M, Wyness L, et al. Factors associated with teenage pregnancy in the European Union countries: a systematic review. Eur J Public Health. 2007;17(6):630–6.

22. Ayele BG, Gebregzabher TG, Hailu TT, Assefa BA. Determinants of teenage pregnancy in Degua Tembien District, Tigray, Northern Ethiopia: A community-based case-control study. PLoS One [Internet]. 2018 Jul 25;13(7):e0200898. Available from: https://doi.org/10.1371/journal.pone.0200898

23. Kassa GM, Arowojolu AO, Odukogbe ATA, Yalew AW. Trends and determinants of teenage childbearing in Ethiopia: evidence from the 2000 to 2016 demographic and health surveys. Ital J Pediatr [Internet]. 2019;45(1):153. Available from: https://doi.org/10.1186/s13052-019-0745-4

24. Birhanu BE, Kebede DL, Kahsay AB, Belachew AB. Predictors of teenage pregnancy in Ethiopia: a multilevel analysis. BMC Public Health [Internet]. 2019;19(1):601. Available from: https://doi.org/10.1186/s12889-019-6845-7

25. Kefale B, Yalew M, Damtie Y, Adane B. A Multilevel Analysis of Factors Associated with Teenage Pregnancy in Ethiopia. Int J Womens Health. 2020;12:785–93.

26. Fikadu Y. Determinants of teenage pregnancy in Ethiopia: A Case–control study, 2019. Issues (St Louis Mo). 2019 Dec 13;17:112.

27. Tigabu S, Liyew AM, Geremew BM. Modeling spatial determinates of teenage pregnancy in Ethiopia; geographically weighted regression. BMC Womens Health [Internet]. 2021;21(1):254. Available from: https://doi.org/10.1186/s12905-021-01400-7

28. Chang Chien YM, Carver S, Comber A. Using geographically weighted models to explore how crowdsourced landscape perceptions relate to landscape physical characteristics. Landsc Urban Plan [Internet]. 2020;203:103904. Available from: https://www.sciencedirect.com/science/article/pii/S0169204620304667

29. Gao Y, Zhao J, Han L. Exploring the spatial heterogeneity of urban heat island effect and its relationship to block morphology with the geographically weighted regression model. Sustain Cities Soc [Internet]. 2022;76:103431. Available from: https://www.sciencedirect.com/science/article/pii/S2210670721007046

30. Fotheringham AS, Yu H, Wolf LJ, Oshan TM, Li Z. On the notion of 'bandwidth' in geographically weighted regression models of spatially varying processes. Int J Geogr Inf Sci [Internet]. 2022 Aug 3;36(8):1485–502. Available from: https://doi.org/10.1080/13658816.2022.2034829

31. Ethiopian Public Health Institute - EPHI, Federal Ministry of Health - FMoH, ICF. Ethiopia Mini Demographic and Health Survey 2019 [Internet]. Addis Ababa, Ethiopia: EPHI/FMoH/ICF; 2021. Available from: https://www.dhsprogram.com/pubs/pdf/FR363/FR363.pdf

32. World Population Review. Countries in Africa [Internet]. 2023. Available from:

https://worldpopulationreview.com/country-rankings/countries-in-africa

33. Croft TN, Marshall AMJ, Allen CK, Arnold F, Assaf S, Balian S. Guide to DHS statistics. Rockv ICF. 2018;645.

34. Merlo J, Chaix B, Ohlsson H, Beckman A, Johnell K, Hjerpe P, et al. A brief conceptual tutorial of multilevel analysis in social epidemiology: using measures of clustering in multilevel logistic regression to investigate contextual phenomena. J Epidemiol Community Health. 2006 Apr;60(4):290–7.

35. Merlo J, Yang M, Chaix B, Lynch J, Råstam L. A brief conceptual tutorial on multilevel analysis in social epidemiology: investigating contextual phenomena in different groups of people. J Epidemiol Community Health [Internet]. 2005 Sep 1;59(9):729 LP – 736. Available from: http://jech.bmj.com/content/59/9/729.abstract

36. Chen Y. New Approaches for Calculating Moran's Index of Spatial Autocorrelation. PLoS One [Internet]. 2013 Jul 12;8(7):e68336. Available from: https://doi.org/10.1371/journal.pone.0068336

37. Zulu LC, Kalipeni E, Johannes E. Analyzing spatial clustering and the spatiotemporal nature and trends of HIV/AIDS prevalence using GIS: the case of Malawi, 1994-2010. BMC Infect Dis [Internet]. 2014;14(1):285. Available from: https://doi.org/10.1186/1471-2334-14-285

38. Srinivasan S. Spatial Regression Models BT - Encyclopedia of GIS. In: Shekhar S, Xiong H, editors. Boston, MA: Springer US; 2008. p. 1102–5. Available from: https://doi.org/10.1007/978-0-387-35973-1_1294

39. Esri. How Exploratory Regression works [Internet]. Available from: https://pro.arcgis.com/en/pro-app/latest/tool-reference/spatial-statistics/how-exploratory-regression-works.htm

40. Tareke AA, Gashu KD, Endehabtu BF. Geographical weighted regression analysis of delayed antenatal care initiation and its factors among all reproductive-aged women in Ethiopia, 2016. Contracept Reprod Med [Internet]. 2022;7(1):23. Available from: https://doi.org/10.1186/s40834-022-00190-z

41. Charlton M, Fotheringham AS, Brunsdon C. Geographically weighted regression white paper. Kildare Natl Univ Irel Maynooth. 2009;1–14.

42. Fotheringham AS, Yang W, Kang W. Multiscale Geographically Weighted Regression (MGWR). Ann Am Assoc Geogr [Internet]. 2017 Nov 2;107(6):1247–65. Available from: https://doi.org/10.1080/24694452.2017.1352480

43. Li Z, Fotheringham AS, Oshan TM, Wolf LJ. Measuring Bandwidth Uncertainty in Multiscale Geographically Weighted Regression Using Akaike Weights. Ann Am Assoc Geogr [Internet]. 2020 Sep 2;110(5):1500–20. Available from: https://doi.org/10.1080/24694452.2019.1704680

44. Lakew Y, Reda AA, Tamene H, Benedict S, Deribe K. Geographical variation and factors influencing modern contraceptive use among married women in Ethiopia: evidence from a national population based survey. Reprod Health [Internet]. 2013;10(1):52. Available from: https://doi.org/10.1186/1742-4755-10-52

45. van der Kwaak A, Baltissen G, Plummer D, Ferris K, Nduba J. Understanding nomadic realities: case studies on sexual and reproductive health and rights in Eastern Africa. KIT

Publishers; 2012.

46. Amegbor PM, Zhang Z, Dalgaard R, Sabel CE. Multilevel and spatial analyses of childhood malnutrition in Uganda: examining individual and contextual factors. Sci Rep [Internet]. 2020;10(1):20019. Available from: https://doi.org/10.1038/s41598-020-76856-y

47. Ketema H, Erulkar A. Married Adolescents and Family Planning in Rural Ethiopia: Understanding Barriers and Opportunities. Afr J Reprod Health. 2018 Dec;22(4):26–34.

48. Chuta N. Neglected experiences: Fertility and childbearing among young people in Ethiopia. 2021;

49. Reading Partners. Early Reading Deficiencies Create Conditions that Enhance Teen Pregnancy Risk [Internet]. 2013. Available from: https://readingpartners.org/blog/early-reading-deficiencies-creating-conditions-that-enhance-teen-pregnancy/

50. Phiri M, Kasonde ME, Moyo N, Sikaluzwe M, Simona S. A multilevel analysis of trends and predictors associated with teenage pregnancy in Zambia (2001–2018). Reprod Health [Internet]. 2023;20(1):16. Available from: https://doi.org/10.1186/s12978-023-01567-2

51. Bennett IM, Frasso R, Bellamy SL, Wortham S, Gross KS. Pre-teen literacy and subsequent teenage childbearing in a US population. Contraception [Internet]. 2013 Apr 1;87(4):459–64. Available from: https://doi.org/10.1016/j.contraception.2012.08.020

52. Odejimi O, Bellingham-Young D. Teenage pregnancy in Africa: trends and determinants in the 21st century. J Heal Soc Care Improv 2016 Vol 1 issue 1 [Internet]. 2016;1(1):12–20. Available from: https://wlv.openrepository.com/bitstream/2436/620300/1/Teenage

Pregnancy in Africa- Trend and Determinants in the 21st Century.pdf

53. Ochen AM, Chi PC, Lawoko S. Predictors of teenage pregnancy among girls aged 13–19 years in Uganda: a community based case-control study. BMC Pregnancy Childbirth [Internet]. 2019;19(1):211. Available from: https://doi.org/10.1186/s12884-019-2347-y

54. Ayoade MA. Spatiotemporal Patterns of Teenage Pregnancy in Nigeria: Evidence from the 2008, 2013 and 2018 NDHS. Pap Appl Geogr [Internet]. 2021 Apr 3;7(2):161–82. Available from: https://doi.org/10.1080/23754931.2020.1852103

55. World Health Organization. Early marriages, adolescent and young pregnancies. Geneva WHO. 2012;1–4.

56. Were M. Determinants of teenage pregnancies: the case of Busia District in Kenya. Econ Hum Biol. 2007 Jul;5(2):322–39.

57. Alemayehu T, Haider J, Habte D. Determinants of adolescent fertility in Ethiopia. Ethiop J Heal Dev. 2010;24(1).

58. Uwizeye D, Muhayiteto R, Kantarama E, Wiehler S, Murangwa Y. Prevalence of teenage pregnancy and the associated contextual correlates in Rwanda. Heliyon. 2020 Oct;6(10):e05037.

59. Nwogwugwu NC. Socio-demographic determinants of adolescent fertility in Zambia. University of the Witwatersrand, Faculty of Humanities; 2013.

60. Domenico DM, Jones KH. Adolescent pregnancy in America: Causes and responses. J Vocat Spec Needs Educ. 2007;30(1):4–12.

61. Bol KN, Negera E, Gedefa AG. Pregnancy among adolescent girls in humanitarian

settings: a case in refugee camp of Gambella regional state, community-based cross-sectional study, Southwest Ethiopia, 2021. BMJ Open [Internet]. 2022 Nov 1;12(11):e064732. Available from: http://bmjopen.bmj.com/content/12/11/e064732.abstract

**Subject: Responses to academic editor's comments**

Dear Dr. Alfredo Luis Fort,

Thank you for taking the time to consider our manuscript titled **"**A multiscale geographically weighted regression analysis of teenage pregnancy and associated factors among adolescents aged 15 to 19 in Ethiopia using the 2019 mini-demographic and health survey**"** for the Plos One Journal original research article. We appreciate the time and effort you have dedicated to providing valuable feedback on our manuscript.

We have taken the comments and concerns into account and made every effort to address them. We agree with all the comments and have incorporated the corresponding revisions into the revised manuscript. We believe that our manuscript has been significantly improved as a result of these revisions, and we hope that our revised manuscript is acceptable for publication in the PLOS One journal.
We would like to thank you once again for your consideration of our work and for inviting us to submit the revised manuscript. We look forward to hearing from you. Our detailed, point-by-point responses to the comments are given below.

Best regards,

Tsion Mulat Tebeje

School of Public Health, Dilla University, Dilla, Ethiopia.

Email: yemarina12@gmail.com (corresponding author)

**Response to journal's requirements**

1. Please ensure that your manuscript meets PLOS ONE's style requirements, including those for file naming.

**Response**: We prepared the manuscript according to the journal's requirements and double-checked its compliance before submitting our revised manuscript.

2. Consider depositing your raw data in a repository to ensure your work is read, appreciated and cited by the largest possible audience. You'll also earn an Accessible Data icon on your published paper if you deposit your data in any participating repository (https://plos.org/open-science/open-data/#accessible-data).

**Response**: Thank you for your suggestion. We have included it in the revised manuscript and highlighted it in the 'Data Availability' section, specifically on lines 417-418, page 25.

3. Please provide additional details regarding participant consent. In the ethics statement in the Methods and online submission information, please ensure that you have specified what type you obtained (for instance, written or verbal, and if verbal, how it was documented and witnessed). If your study included minors, state whether you obtained consent from parents or guardians. If the need for consent was waived by the ethics committee, please include this information.

**Response**: This study involved a secondary data analysis using publicly available data from the MEASURE DHS program and ethical approval and participant consent was not required. This is highlighted in the ethical approval and consent to participate section (lines 194-196, page 9). The data files do not contain any names of individuals or household addresses. We obtained approval from the DHS program to use the dataset and have attached the proof of granted permission as an "other" file with our submission.

4. Your ethics statement should only appear in the Methods section of your manuscript. If your ethics statement is written in any section besides the Methods, please move it to the Methods section and delete it from any other section. Please ensure that your ethics statement is included in your manuscript, as the ethics statement entered into the online submission form will not be published alongside your manuscript.

**Response**: Thank you for the suggestion. We have moved the ethics statement to the methods section in the revised manuscript.

5. We note that Figures 2,3,4 and 5 in your submission contain [map/satellite] images which may be copyrighted. All PLOS content is published under the Creative Commons Attribution License (CC BY 4.0), which means that the manuscript, images, and Supporting Information

files will be freely available online, and any third party is permitted to access, download, copy, distribute, and use these materials in any way, even commercially, with proper attribution. For these reasons, we cannot publish previously copyrighted maps or satellite images created using proprietary data, such as Google software (Google Maps, Street View, and Earth).
We require you to either (1) present written permission from the copyright holder to publish these figures specifically under the CC BY 4.0 license, or (2) remove the figures from your submission:
You may seek permission from the original copyright holder of Figures 2,3,4 and 5 to publish the content specifically under the CC BY 4.0 license.

**Response**: We appreciate your concern regarding the ethical issues. However, the figures (2, 3, 4, and 5) mentioned in our manuscript are not copyrighted; rather, they are the results of spatial analysis conducted using ArcGIS and Python software. The DHS geographic information system, which contains shapefiles with coordinates (latitude and longitude), were obtained from the DHS office by explaining the objective of the study through online requests. To obtain these figures, we imported relevant data extracted from the 2019 Ethiopian mini Demographic and Health Survey. We have attached the permission letter obtained from MEASURE DHS to use the EDHS dataset and geographical location as an "other" file labeled 'AuthLetter_188507'. The Ethiopian administrative boundaries shapefile was obtained from the Ethiopian Central Statistical Agency (CSA). The shape file used to construct the figures can be accessed publicly at https://africaopendata.org/dataset/ethiopia-shapefiles. We incorporated and highlighted this information in captions of figures 2, 3, 4, and 5.

As a result, the maps presented in our study are not copyrighted. Instead, they are the findings of our spatial analysis, which we performed using ArcGIS and Python on shapefiles and projected CSV files of coordinates. This is the exact process that we followed when conducting our study. Therefore, we can confirm that the figures presented in our study represent our spatial analysis results and are not copyrighted.

**Edits requested on the submission**

1. We note your current data availability statement: "The data used in this research are publicly available from the measure DHS program via an online request at http://www.dhsprogram.com" As the URL presented in your Data Availability Statement and cover letter links to the DHS Program general website, please provide any direct URL(s), accession numbers, instructions, and additional information (DOI, data set title, etc.) required for researchers to access the specific data underlying the results presented in your study.

**Response**: Thank you for the suggestion. We have edited the data availability statement by providing the direct URL which links to the login or register for datasets. By registering and logging in, anyone can directly access the dataset.

2. Please clarify if the data used for the maps are copyrighted/what copyright information or restrictions are attached to it. If the data is copyrighted then please send the below permission form to the owners of the data.

**Response**: Thank you for asking for clarification. The maps included in our study are not copyrighted from somewhere. They were the output of our work created using ArcGIS version 10.7 and Python 3 software based on the maps on the Ethiopian shapefile obtained from the Ethiopian Central Statistical Agency (CSA), which is publicly available on Open-Africa https://africaopendata.org/dataset/ethiopia-shapefiles, and the GPS data obtained from the measure DHS program after submitting a request along with the study's rationale. Therefore, we assure you that the maps presented in this paper are the result of our work, utilizing appropriate analytical methods and procedures. Furthermore, we have acknowledged the source of the data, and we, as authors, have previously published studies containing similar maps.

**Response to editor's comments**

1. I have seen a similar study like this, with the same problems. The topic and method plus results are of extreme importance. However, we cannot invite Reviewers because they will probably reject the manuscript because, like me, they will find lots of errors, misspellings, and inadequate descriptions. Please take the manuscript to someone who can write proper English, and use my first suggestions (in attached file) to continue improving the wording and descriptions for the rest of the file.

**Response**: Thank you for giving us your valuable time and for sharing your valuable input. We fully agree with your suggestion. We have thoroughly reviewed existing similar studies and have appropriately cited them. In the final paragraph of the introduction section, we elucidated how our study differs. The revised manuscript has been meticulously edited and proofread to rectify quality issues, such as misspellings, grammatical errors, and unclear sentences.
