# Supplementary material for: A multiscale geographically weighted regression analysis of teenage pregnancy and associated factors among adolescents aged 15 to 19 in Ethiopia using the 2019 mini-demographic and health survey
Source: PLoS One. 2024 Sep 6;19(9):e0310025. doi: 10.1371/journal.pone.0310025 (PMC11379268; doi:10.1371/journal.pone.0310025)
Supplement: Attachment — Submitted filename: Response to Reviewers.docx [file pone.0310025.s002.docx]

**Subject: Responses to academic editor's comments**

Dear Dr. Alfredo Luis Fort,

Thank you for taking the time to consider our manuscript titled **“**A multiscale geographically weighted regression analysis of teenage pregnancy and associated factors among adolescents aged 15 to 19 in Ethiopia using the 2019 mini-demographic and health survey**”** for the Plos One Journal original research article. We appreciate the time and effort you have dedicated to providing valuable feedback on our manuscript.

We have taken the comments and concerns into account and made every effort to address them. We agree with all the comments and have incorporated the corresponding revisions into the revised manuscript. We believe that our manuscript has been significantly improved as a result of these revisions, and we hope that our revised manuscript is acceptable for publication in the PLOS One journal.

We would like to thank you once again for your consideration of our work and for inviting us to submit the revised manuscript. We look forward to hearing from you. Our detailed, point-by-point responses to the comments are given below.

Best regards,

Tsion Mulat Tebeje

School of Public Health, Dilla University, Dilla, Ethiopia.

Email: [yemarina12@gmail.com](mailto:yemarina12@gmail.com) (corresponding author)

**Response to journal’s requirements**

1. Please ensure that your manuscript meets PLOS ONE's style requirements, including those for file naming.

**Response**: We prepared the manuscript according to the journal's requirements and double-checked its compliance before submitting our revised manuscript.

2. Consider depositing your raw data in a repository to ensure your work is read, appreciated and cited by the largest possible audience. You’ll also earn an Accessible Data icon on your published paper if you deposit your data in any participating repository (<https://plos.org/open-science/open-data/#accessible-data>).

**Response**: Thank you for your suggestion. We have included it in the revised manuscript and highlighted it in the 'Data Availability' section, specifically on lines 417-418, page 25.

3. Please provide additional details regarding participant consent. In the ethics statement in the Methods and online submission information, please ensure that you have specified what type you obtained (for instance, written or verbal, and if verbal, how it was documented and witnessed). If your study included minors, state whether you obtained consent from parents or guardians. If the need for consent was waived by the ethics committee, please include this information.

**Response**: This study involved a secondary data analysis using publicly available data from the MEASURE DHS program and ethical approval and participant consent was not required. This is highlighted in the ethical approval and consent to participate section (lines 194-196, page 9). The data files do not contain any names of individuals or household addresses. We obtained approval from the DHS program to use the dataset and have attached the proof of granted permission as an “other” file with our submission.

4. Your ethics statement should only appear in the Methods section of your manuscript. If your ethics statement is written in any section besides the Methods, please move it to the Methods section and delete it from any other section. Please ensure that your ethics statement is included in your manuscript, as the ethics statement entered into the online submission form will not be published alongside your manuscript.

**Response**: Thank you for the suggestion. We have moved the ethics statement to the methods section in the revised manuscript.

5. We note that Figures 2,3,4 and 5 in your submission contain [map/satellite] images which may be copyrighted. All PLOS content is published under the Creative Commons Attribution License (CC BY 4.0), which means that the manuscript, images, and Supporting Information files will be freely available online, and any third party is permitted to access, download, copy, distribute, and use these materials in any way, even commercially, with proper attribution. For these reasons, we cannot publish previously copyrighted maps or satellite images created using proprietary data, such as Google software (Google Maps, Street View, and Earth).

We require you to either (1) present written permission from the copyright holder to publish these figures specifically under the CC BY 4.0 license, or (2) remove the figures from your submission:

You may seek permission from the original copyright holder of Figures 2,3,4 and 5 to publish the content specifically under the CC BY 4.0 license.

**Response**: We appreciate your concern regarding the ethical issues. However, the figures (2, 3, 4, and 5) mentioned in our manuscript are not copyrighted; rather, they are the results of spatial analysis conducted using ArcGIS and Python software. The DHS geographic information system, which contains shapefiles with coordinates (latitude and longitude), were obtained from the DHS office by explaining the objective of the study through online requests. To obtain these figures, we imported relevant data extracted from the 2019 Ethiopian mini Demographic and Health Survey. We have attached the permission letter obtained from MEASURE DHS to use the EDHS dataset and geographical location as an “other” file labeled 'AuthLetter_188507'. The Ethiopian administrative boundaries shapefile was obtained from the Ethiopian Central Statistical Agency (CSA). The shape file used to construct the figures can be accessed publicly at <https://africaopendata.org/dataset/ethiopia-shapefiles>. We incorporated and highlighted this information in captions of figures 2, 3, 4, and 5.

As a result, the maps presented in our study are not copyrighted. Instead, they are the findings of our spatial analysis, which we performed using ArcGIS and Python on shapefiles and projected CSV files of coordinates. This is the exact process that we followed when conducting our study. Therefore, we can confirm that the figures presented in our study represent our spatial analysis results and are not copyrighted.

**Edits requested on the submission**

1. We note your current data availability statement:
   "The data used in this research are publicly available from the measure DHS program
   via an online request at [http://www.dhsprogram.com](http://www.dhsprogram.com/)"
   As the URL presented in your Data Availability Statement and cover letter links to the DHS Program general website, please provide any direct URL(s), accession numbers, instructions, and additional information (DOI, data set title, etc.) required for researchers to access the specific data underlying the results presented in your study.

**Response**: Thank you for the suggestion. We have edited the data availability statement by providing the direct URL which links to the login or register for datasets. By registering and logging in, anyone can directly access the dataset.

2. Please clarify if the data used for the maps are copyrighted/what copyright information or restrictions are attached to it. If the data is copyrighted then please send the below permission form to the owners of the data.

**Response**: Thank you for asking for clarification. The maps included in our study are not copyrighted from somewhere. They were the output of our work created using ArcGIS version 10.7 and Python 3 software based on the maps on the Ethiopian shapefile obtained from the Ethiopian Central Statistical Agency (CSA), which is publicly available on Open-Africa <https://africaopendata.org/dataset/ethiopia-shapefiles>, and the GPS data obtained from the measure DHS program after submitting a request along with the study's rationale. Therefore, we assure you that the maps presented in this paper are the result of our work, utilizing appropriate analytical methods and procedures. Furthermore, we have acknowledged the source of the data, and we, as authors, have previously published studies containing similar maps.

**Response to editor’s comments**

1. I have seen a similar study like this, with the same problems. The topic and method plus results are of extreme importance. However, we cannot invite Reviewers because they will probably reject the manuscript because, like me, they will find lots of errors, misspellings, and inadequate descriptions. Please take the manuscript to someone who can write proper English, and use my first suggestions (in attached file) to continue improving the wording and descriptions for the rest of the file.

**Response**: Thank you for giving us your valuable time and for sharing your valuable input. We fully agree with your suggestion. We have thoroughly reviewed existing similar studies and have appropriately cited them. In the final paragraph of the introduction section, we elucidated how our study differs. The revised manuscript has been meticulously edited and proofread to rectify quality issues, such as misspellings, grammatical errors, and unclear sentences.
